# Supplementary material for: Cost-effectiveness of single-layer versus double-layer uterine closure during caesarean section on postmenstrual spotting: economic evaluation alongside a randomised controlled trial
Source: BMJ Open. 2021 Jul 2;11(7):e044340. doi: 10.1136/bmjopen-2020-044340 (PMC8256741; doi:10.1136/bmjopen-2020-044340)
Supplement: Supplementary data [file bmjopen-2020-044340supp001.pdf]

Supplementary appendix

Appendix S1. List of collaborators.

**Hospital**	**Site principal investigator**
Amphia hospital, Breda	Dimitri NM Papatsonis
Amsterdam UMC, Univ of Amsterdam, Amsterdam	Eva Pajkrt
Amsterdam UMC, VU University, Amsterdam	Wouter JK Hehenkamp
Bernhoven hospital, Uden	Angèle LM Oei
Birth Centre Wilhelmina Children Hospital/University Medical Centre Utrecht, Utrecht	Mireille N Bekker
Canisius-Wilhelmina hospital, Nijmegen	Daniela H Schippers
Catharina hospital, Eindhoven	Huib AAM van Vliet
Deventer ziekenhuis, Deventer	Lucet van der Voet
Diakonessenhuis, Utrecht	Nico WE Schuitemaker
Dijklander hospital – location Hoorn	Majoie Hemelaar
Flevo hospital, Almere	WM (Marchien) van Baal
Gelre hospital – location Apeldoorn	Anjoke JM Huisjes
Gelre hospital – location Zutphen	Wouter J Meijer
Groene Hart hospital, Gouda	CAH (Ineke) Janssen
Haaglanden Medical Centre – Westeinde hospital, Den Haag	Wietske Hermes
Haga hospital, Den Haag	AH (Hanneke) Feitsma
Isala clinics, Zwolle	Hugo WF van Eijndhoven
Jeroen Bosch hospital, 's-Hertogenbosch	Robbert JP Rijnders
Leiden University Medical Centre, Leiden	Marieke Sueters
Maastricht University Medical Centre, Research school 'GROW', Maastricht	HCJ (Liesbeth) Scheepers
Máxima Medical Centre, Veldhoven	Judith OEH van Laar
Meander Medical Centre, Amersfoort	Elisabeth MA Boormans
OLVG-oost, Amsterdam	Paul JM van Kesteren
OLVG-west, Amsterdam	Celine M Radder
Radboud University Nijmegen Medical Centre, Nijmegen	Esther Hink
Reinier de Graaf hospital, Delft	Kitty Kapiteijn
Rijnstate hospital, Arnhem	Karin de Boer
Röpcke-Zweers hospital, Hardenberg	Mesrure Kaplan
Sint Antonius Hospital, Nieuwegein	Erik van Beek
Sint Franciscus Hospital, Rotterdam	LHM (Marloes) de Vleeschouwer
Tergooi hospital, Blaricum	Harry Visser
Zuyderland Medical Centre, Heerlen	Josje Langenveld

Supplementary tables

Table S1: Costs of the intervention per participant

Resources	Units	Unit price, €	Costs, €
Additional suture material	One piece	6.14	6.14
Additional operation time, academic hospital (3.9 minutes)	Hour	1379.27	89.65
Total costs per participant in academic hospital			95.79
Additional operation time, non-academic hospital (3.9 minutes)	Hour	1000	65.00
Total costs per participant in non-academic hospital			71.14

Table S2: Probability of the intervention being cost-effective at different willingness-to-pay thresholds for spotting days

	Main analyses – spotting days	
WTP €/ unit of effect gained	***Societal Perspective***	***Healthcare perspective***
0	0.30	0.55
31.6 (1 working hour)	0.30	0.56
126.4 (4 working hours)	0.31	0.58
252.8 (8 working hours)	0.31	0.59
	**Main analyses - QALYs**	
0	0.30	0.55
10000	0.27	0.41
20000	0.25	0.32
30000	0.24	0.27
40000	0.22	0.24
50000	0.21	0.22

WTP=willingness-to-pay; QALY=quality-adjusted life-years

Table S3: Description of follow-up complete cases and missing cases by effect and cost outcomes without excluding cases of amenorrhoea

	Complete cases			Missing cases (%)		
	Intervention	***Control***	***Total***	***Intervention***	***Control***	***Total***
Spotting days	936	940	1876	212 (23)	204 (22)	416 (22)
QALY	845	851	1696	303 (36)	293 (34)	596 (35)
Primary care costs	913	924	1837	235 (26)	220 (24)	455 (25)
Secondary care costs	838	833	1671	310 (37)	311 (37)	621 (37)
Medication costs	834	825	1659	314 (38)	319 (39)	633 (38)
Total healthcare costs	830	823	1653	318 (38)	321 (39)	639 (39)
Informal care costs	913	924	1837	235 (26)	220 (24)	455 (25)
Absenteeism costs at work	852	849	1701	296 (35)	295 (35)	591 (35)
Absenteeism costs at unpaid work	905	921	1826	243 (27)	223 (24)	466 (25)
Presenteeism costs	680	690	1370	468 (69)	454 (66)	922 (67)
Total lost productivity costs	677	689	1366	471 (69)	455 (66)	926 (68)
Total societal costs	664	671	1335	484 (73)	473 (70)	957 (72)
