# Supplementary material for: Cost-effectiveness of single-layer versus double-layer uterine closure during caesarean section on postmenstrual spotting: economic evaluation alongside a randomised controlled trial
Source: BMJ Open. 2021 Jul 2;11(7):e044340. doi: 10.1136/bmjopen-2020-044340 (PMC8256741; doi:10.1136/bmjopen-2020-044340)
Supplement: Author's manuscript [file bmjopen-2020-044340.draft_revisions.pdf]

BMJ Open is committed to open peer review. As part of this commitment we make the peer review history of every article we publish publicly available.

When an article is published we post the peer reviewers' comments and the authors' responses online. We also post the versions of the paper that were used during peer review. These are the versions that the peer review comments apply to.

The versions of the paper that follow are the versions that were submitted during the peer review process. They are not the versions of record or the final published versions. They should not be cited or distributed as the published version of this manuscript.

BMJ Open is an open access journal and the full, final, typeset and author-corrected version of record of the manuscript is available on our site with no access controls, subscription charges or pay-per-view fees (http://bmjopen.bmj.com).

If you have any questions on BMJ Open's open peer review process please email info.bmjopen@bmj.com

BMJ Open

Cost-effectiveness of single- versus double-layer uterine closure during caesarean section on postmenstrual spotting: economic evaluation alongside a randomised controlled trial

Journal:	*BMJ Open*
Manuscript ID	bmjopen-2020-044340
Article Type:	Original research
Date Submitted by the Author:	31-Aug-2020

Complete List of Authors:	Stegwee, Sanne; Amsterdam UMC Locatie VUmc, Obstetrics and Gynaecology<br>Ben, Ângela; Vrije Universiteit Amsterdam, Health Sciences<br>El Alili, Mohamed ; Vrije Universiteit Amsterdam<br>van der Voet, Lucet; Deventer Ziekenhuis, Obstetrics and Gynaecology<br>de Groot, Christianne; Amsterdam UMC Locatie VUmc, Obstetrics and Gynaecology<br>Bosmans, J; Vrije Universiteit Amsterdam, Health Sciences<br>Huirne, Judith; Amsterdam UMC Locatie AMC, Obstetrics and Gynaecology

Keywords:	OBSTETRICS, Maternal medicine < OBSTETRICS, Ultrasonography < OBSTETRICS, HEALTH ECONOMICS

SCHOLARONE™
Manuscripts

I, the Submitting Author has the right to grant and does grant on behalf of all authors of the Work (as defined in the below author licence), an exclusive licence and/or a non-exclusive licence for contributions from authors who are: i) UK Crown employees; ii) where BMJ has agreed a CC-BY licence shall apply, and/or iii) in accordance with the terms applicable for US Federal Government officers or employees acting as part of their official duties; on a worldwide, perpetual, irrevocable, royalty-free basis to BMJ Publishing Group Ltd ("BMJ") its licensees and where the relevant Journal is co-owned by BMJ to the co-owners of the Journal, to publish the Work in this journal and any other BMJ products and to exploit all rights, as set out in our licence.

The Submitting Author accepts and understands that any supply made under these terms is made by BMJ to the Submitting Author unless you are acting as an employee on behalf of your employer or a postgraduate student of an affiliated institution which is paying any applicable article publishing charge ("APC") for Open Access articles. Where the Submitting Author wishes to make the Work available on an Open Access basis (and intends to pay the relevant APC), the terms of reuse of such Open Access shall be governed by a Creative Commons licence – details of these licences and which Creative Commons licence will apply to this Work are set out in our licence referred to above.

Other than as permitted in any relevant BMJ Author's Self Archiving Policies, I confirm this Work has not been accepted for publication elsewhere, is not being considered for publication elsewhere and does not duplicate material already published. I confirm all authors consent to publication of this Work and authorise the granting of this licence.

For peer review only

Cost-effectiveness of single- versus double-layer uterine closure during caesarean section on postmenstrual spotting: economic evaluation alongside a randomised controlled trial

Sanne I Stegwee[1]*, Ângela Jornada Ben[2], Mohamed El Alili[2], Lucet F van der Voet[3], Christianne JM de Groot[1], Judith E Bosmans[2], Judith AF Huirne[1]*, for the 2Close study group†

[1] Department of Obstetrics and Gynaecology, Amsterdam UMC, Vrije Universiteit, Amsterdam Reproduction & Development, Amsterdam, Netherlands

[2] Department of Health Sciences, Amsterdam Public Health, Faculty of Science, VU University, Amsterdam, The Netherlands

[3] Department of Obstetrics and Gynaecology, Deventer Hospital, Deventer, Netherlands

*Corresponding author during submission: SI Stegwee, MD, Amsterdam UMC - VU University, Department of Obstetrics and Gynaecology, De Boelelaan 1117, 1081 HV Amsterdam, The Netherlands. T: +31 20 4444807, F: +31 20 444 83 87, s.stegwee@amsterdamumc.nl.

*Corresponding author after publication: Prof JAF Huirne, Amsterdam UMC - VU University, Department of Obstetrics and Gynaecology, De Boelelaan 1117, 1081 HV Amsterdam, The Netherlands. T: +31 20 4444444, F: +31 20 444 83 87, j.huirne@amsterdamumc.nl.

†A full list of collaborators is provided in the appendix

For peer review only - http://bmjopen.bmj.com/site/about/guidelines.xhtml

Abstract

Objective: To evaluate the cost-effectiveness of double-layer compared to single-layer uterine closure after a first caesarean section (CS) from a societal and healthcare perspective.

Design: Economic evaluation alongside a multicentre, double-blind, randomised controlled trial

Setting: 32 hospitals in the Netherlands, 2016-18.

Participants: 2292 women ≥ 18 years undergoing a first CS were randomly assigned (1:1). Exclusion criteria were: inability for counselling, previous uterine surgery, known menstrual disorder, placenta increta or percreta, pregnant with three or more fetuses. 1144 women were assigned to single-layer and 1148 to double-layer closure.

Interventions: Single-layer unlocked uterine closure and double-layer unlocked uterine closure with the second layer imbricating the first.

Main outcome measures: Spotting days, quality-adjusted life-years (QALYs), and societal costs at nine months of follow-up. Missing data were imputed using multiple imputation.

Results: No significant differences were found between single- versus double-layer closure in mean spotting days (1.42 and 1.39 days; mean difference [md] -0.032, 95% CI, -0.342; 0.279), QALYs (0.663 and 0.659; md -0.004, 95% CI, -0.013; 0.005), total healthcare costs (€742 and €733; md €-9, 95% CI, -250; 144), and total societal costs (€5408 and €5711; md €303, 95% CI, -413; 1035). The probability of the intervention being cost-effective at ceiling ratios of 0, 10,000 and 20,000 €/QALY gained was 0.23, 0.21 and 0.19, respectively, (societal perspective), and 0.53, 0.40 and 0.32, respectively, (healthcare perspective).

Conclusion: Double-layer uterine closure is not cost-effective compared to single-layer uterine closure from both perspectives. If this is confirmed by our long-term reproductive follow-up, we suggest to adjust uterine closure technique guidelines.

Trial registration: Netherlands Trial Register (NTR5480/NL5380; trial finished).

Key words: caesarean section; uterine closure technique; postmenstrual spotting; cost-effectiveness analysis

For peer review only - http://bmjopen.bmj.com/site/about/guidelines.xhtml

Article summary

Strengths and limitations of this study

This study has been performed alongside large multicentre RCT, which is considered the best vehicle for economic evaluations

We prospectively collected data regarding costs and effect, and we used patient level information

All relevant costs for decision making were included in the analysis, to conduct an analysis form a societal perspective

Possible recall bias due to retrospective self-reported questionnaire over 3- and 6-month period

Generalizability of the results to other populations may be limited, as other healthcare practices and payment systems may exist

For peer review only

Introduction

Caesarean section (CS) rates rise globally and is the mode of delivery for approximately one in five live births globally.[1 2] As a consequence, a rise in morbidity related to CS is observed as well.[3] Severe morbidity associated with subsequent pregnancy includes placenta accrete disorders and uterine rupture. However, less severe but more prevalent gynaecological morbidity related to a CS have recently gained more interest as well. Chronic maternal morbidity after CS includes dysmenorrhoea and abnormal uterine bleeding, which are both associated with a sonographically visible indentation at the site of the previous uterine incision. This indentation is called a niche and is seen in approximately 60% of women after CS.[4 5] Of them, 30% develops abnormal uterine bleeding and more specifically, postmenstrual spotting.[6 7] This is brownish discharge at the end of the menstruation or blood loss in between two menstruations that is limiting women in daily life.[6] Over the last years, an increase in the development of medical treatments and surgical procedures to treat or remove the niche is observed, primarily aiming to reduce spotting.[8]

CS is the most common major surgical intervention.[9] However, there is no international guideline on the most optimal way to close the uterine incision while the specific closure technique may influence healing of the uterine wound. A specific issue on which no consensus exists is whether to use single- or double-layer closure of the uterine layers.[10 11] When comparing these techniques, no differences were found at short-term except for longer operation time after double-layer closure.[12 13] Nevertheless, previous studies also suggested that double-layer closure may result in better uterine scar healing and lower prevalence of large niches thereby possibly leading to lower medical costs than single-layer closure.[12 14] There is, however, a lack of studies on uterine closure techniques and their impact on maternal health outcomes related to gynaecological symptoms.[13] The impact of different uterine closure techniques on healthcare utilization, informal care, and lost productivity costs has never been investigated previously. As decision makers increasingly demand evidence of cost-effectiveness of health care interventions, conducting economic analysis alongside clinical trials is desirable because it allows the prospective collection of cost and effect data and the use of patient

For peer review only - http://bmjopen.bmj.com/site/about/guidelines.xhtml

level information for drawing inferences about additional costs and benefits of interventions.[15] In addition, regulatory and reimbursement agencies of many countries consider evidence of economic value along with clinical effectiveness.[15]

Therefore, the aim of this study was to perform a cost-effectiveness analysis of double-layer compared to single-layer uterine closure after a first CS from both a societal and healthcare perspective. We hypothesised that double-layer closure would reduce postmenstrual spotting and total societal and healthcare costs compared to single-layer closure as a result of less morbidity, despite slightly higher intervention costs of double-layer closure.

For peer review only

For peer review only - http://bmjopen.bmj.com/site/about/guidelines.xhtml

Methods

Study design

An economic evaluation was performed alongside a multicentre randomised controlled superiority trial comparing double-layer closure and single-layer uterine closure after a first CS. The study protocol has been published elsewhere.[16] The study was approved by the Institutional Review Board (IRB) of Amsterdam UMC – location VU University medical centre (registration number 2015.462) and by the boards of all participating hospitals. No substantial changes were made to the protocol after commencement of the trial.[16] This trial-based economic evaluation is reported according to the Consolidated Health Economic Evaluating Reporting Standards (CHEERS) statement.[17]

Target population

All women who underwent a first CS, planned or unplanned, at one of the participating hospitals were asked to participate in the study. Inclusion criteria were sufficient command of the Dutch or English language, age 18 years or older and written informed consent. Exclusion criteria were: inadequate possibility for counselling (e.g., indication for emergency CS without being informed about the study previously, women in severe pain without adequate therapy), previous major uterine surgery (e.g., laparoscopic or laparotomic fibroid resection, septum resection), women with known causes of menstrual disorders (e.g., cervical dysplasia, communicating hydrosalpinx, uterine anomaly or endocrine disorders disturbing ovulation), placenta increta or percreta during the current pregnancy, or three or more foetuses during the current pregnancy. After informed consent had been signed and a CS was indicated, participants were randomly allocated to receive single-layer (control) or double-layer (intervention) closure of the uterine incision in a 1:1 ratio. Due to the nature of the treatment, surgeons performing the CS were not masked to the allocated method. Participants and sonographers were blinded to the allocation, researchers and statisticians were not. Detailed information about study design and randomization can be found in the study protocol.[16]

For peer review only - http://bmjopen.bmj.com/site/about/guidelines.xhtml

Choice of health outcomes

For this trial-based economic evaluation, two main health outcomes were used: postmenstrual spotting (referred to as spotting days in this paper) and Quality-Adjusted Life-Years (QALYs) at nine months after CS. Spotting days was chosen because it has been strongly related to a niche[6 7] (i.e., an indentation at the site of the caesarean scar with a depth of at least 2 mm[18]), which may be influenced by uterine closure technique.[5] QALY is routinely used as a summary outcome measure of health in economic evaluations, because it incorporates the impact of interventions on both the quantity and quality of life,[19] and allows decision makers to compare the effectiveness and cost-effectiveness of a range of interventions for different health conditions.[20]

Study perspective and time-horizon

This trial-based economic evaluation was performed from a societal and a healthcare perspective over a time-horizon of nine months. Therefore, discounting of costs and effects was not necessary. When a healthcare perspective is adopted, only the intervention costs and costs related to healthcare utilization are included in the analysis.[21] For the societal perspective, costs related to informal care and productivity losses are included in addition to intervention and healthcare utilization costs.[21]

Setting and location

In total, 32 hospitals in the Netherlands, both academic (n=6) and non-academic (n=26), collaborating within the Dutch Consortium for Healthcare Evaluation and Research in Obstetrics and Gynaecology (NVOG Consortium 2.0, www.zorgevaluatienederland.nl), participated in this study.[16] In the Netherlands, a CS is only performed in a hospital setting. In most cases, without maternal or neonatal complications, women will be discharged from the hospital after a CS after two or three days. All costs regarding the CS and admission days are standard care and are paid by an individual's health insurance. Maternity leave of at least ten weeks is regulated through the Employee Insurance Agency. Paternity

For peer review only - http://bmjopen.bmj.com/site/about/guidelines.xhtml

leave is limited to one week. The first eight days after delivery, a maternity nurse visits the family at least three hours a day.

Control and intervention condition

The control group underwent single-layer closure of the uterus using unlocked continuous running multifilament sutures, which is the usual care provided by hospitals in the Netherlands.[16] In the intervention group, double-layer closure of the uterine incision was performed using unlocked multifilament continuous running sutures for both layers and the endometrial layer was included in the first layer. The second layer was a continuous running suture that imbricated the first. A mandatory online instruction video was shown to all surgeons in participating hospitals prior to participation for the intervention group. The exact procedures in both study arms regarding uterine closure are described in the study protocol.[16]

At baseline, data was collected on sociodemographic characteristics for all participants.[16]

Outcomes

<u>Health outcomes</u>

Spotting days was the primary outcome of the trial, and was defined as number of days with brownish discharge for more than two days at the end of the menstruation, with a total duration (menstruation and spotting) of more than seven days, or intermenstrual blood loss that started after the end of the menstruation.[6] Spotting days were self-reported by participants through a digital questionnaire at nine months after CS, including a calendar on which women could record daily blood loss during one month. Women who reported that they had no blood loss were classified as amenorrhoeic.

Health-related quality of life was measured using the EQ-5D-5L at baseline, and at three and nine months after CS.[22] The EQ-5D-5L has five dimensions of quality of life (mobility, self-care, usual activities, pain/discomfort, and anxiety/depression) using five response levels (i.e., no problems, slight problems, moderate problems, severe problems, or extreme problems) describing 234 health states.[22]

For peer review only - http://bmjopen.bmj.com/site/about/guidelines.xhtml

The participants' health states obtained from EQ-5D-5L responses were converted into utility values using the Dutch tariff.[23] The utility values were used to calculate QALYs by means of the area under the curve method (i.e., the duration of a health state is multiplied by the utility related to that health state).

Cost outcomes

Intervention costs

The average costs of performing a CS reported by the participating hospitals was €5360. The intervention incurred additional suture material and additional operation time (3.9 minutes on average). The costs of additional resources were obtained from the academic and non-academic hospitals using a bottom-up approach. On average, the intervention resulted in additional costs of €95.79 per participant in academic hospitals and €71.14 per participant in non-academic hospitals (Supplementary Table S1).

Healthcare utilization and informal care costs

A specifically adapted version of the iMTA Medical Cost Questionnaire (iMCQ)[24] was used to measure healthcare utilization and care provided by family and/or friends (i.e., informal care) using 3-month and 6-month recall periods at 3 and 9 months of follow-up, respectively. The iMCQ is a standardized generic instrument for measuring medical costs including questions related to healthcare utilization and informal care.[24] Healthcare utilization was valued using prices from the Dutch costing guideline.[25] Healthcare ultilization costs included primary care costs (e.g., costs of visits to general practitioners, health professionals, and complementary healthcare providers), secondary care costs (e.g., costs of ambulatory hospital visits, visits to other healthcare organizations and admissions to the hospital), and medication costs. Secondary care costs were recorded after discharge from the hospital. The average costs of performing the CS included the operation and hospital stay until discharge and were,

For peer review only - http://bmjopen.bmj.com/site/about/guidelines.xhtml

therefore, not included in the secondary care costs to avoid double-counting. There were no differences in days of hospital stay between the groups. (ref effect paper)

The informal care costs were based on the amount of time the participant needed help in performing household tasks or received care from family and/or friends, because of health problems. Dutch standard prices were used for informal care costs.[25] Medication use was valued using data from the Dutch Healthcare Institute (www.medicijnkosten.nl).[26]

Lost productivity costs

The iMTA Productivity Cost Questionnaire (iPCQ)[27] was used to measure self-reported sickness absenteeism from paid and unpaid work, and presenteeism using 3-month and 6-month recall periods at 3 and 9 months of follow-up, respectively. The iPCQ is a standardized generic questionnaire to measure productivity costs and it is applicable to national and international studies.[27] The friction cost approach (FCA) was used to calculate sickness absenteeism costs from paid work.[28] The FCA assumes that sickness absenteeism costs are limited to the period needed to replace an absent, sick worker (the friction period), which has been estimated to be 12 weeks (85 days) in the Netherlands.[25] Gender-specific estimates of the mean wages of the Dutch population were used to calculate sickness absenteeism costs from paid work.[25]

To measure sickness absenteeism from unpaid work, the participants were asked whether they had difficulty in performing unpaid work activities due to sickness (e.g., household tasks, childcare, voluntary work), and if that was the case, for how many hours.[27] Costs related to sickness absenteeism from unpaid work were valued using a shadow price for legally employing a domestic assistant.[27] To measure participants' level of presenteeism, participants rated how efficiently they worked while suffering from health complaints on a scale from 0 (I was unable to do anything) to 10 (I was able to do as much as usual). The resulting efficiency score was used to calculate presenteeism costs: Presenteeism costs = number of days working with complaints * [1 - (efficiency score / 10)] * number of working hours per day * gender-specific mean wage rates.[29]

For peer review only - http://bmjopen.bmj.com/site/about/guidelines.xhtml

Statistical analysis

Analyses were performed according to the intention-to-treat principle using StataSE 16® (StataCorp LP, CollegeStation, TX, US). Multiple Imputation by Chained Equations (MICE), stratified by group allocation, was used to impute missing data. Variables associated with missingness and outcomes as well as potential confounders were included in the imputation model such as age, BMI, level of education, parity, previous miscarriage, gestational age at CS, hypertension, and diabetes. Predictive Mean Matching was used in the imputation procedure to account for the skewed distribution of the costs.[30] The number of imputations was increased until there was a loss of efficiency of ≤5%, resulting in twenty imputed datasets.[30 31] The twenty datasets were analyzed separately and estimates were pooled using Rubin's rules.[32] After multiple imputation, amenorrhoeic women were excluded from the analyses as a priori decided, because the outcome spotting days could not be evaluated in these women.[16]

Differences in costs and effects between treatment groups at nine months follow-up were estimated using seemingly unrelated regression analyses, which accounts for the correlation between costs and effects.[33] The intraclass correlation coefficient (ICC, i.e., the variation around the subjects belonging to the same hospital cluster divided by the total variance between hospitals)[34] was small (ICC=0.004). This means that hardly any of the variance in the outcome measure was accounted for by clustering at the level of the hospital. In addition to the small ICC, patients were randomised at the individual level and not at the hospital level. Therefore, multilevel analysis was not necessary. Incremental Cost-Effectiveness Ratios (ICERs) were calculated by dividing the difference in costs (i.e., total societal costs and total healthcare costs) between groups by the difference in effects.

Bias-corrected accelerated bootstrapping with 5,000 replications was used to estimate the joint uncertainty surrounding differences in costs and effects. Bootstrapped cost-effect pairs were plotted on cost-effectiveness planes (CE-planes).[35] Cost-effectiveness acceptability curves (CEACs) were estimated that show the probability of double-layer closure being cost-effective compared to single-

For peer review only - http://bmjopen.bmj.com/site/about/guidelines.xhtml

layer closure for a range of willingness-to-pay (WTP) thresholds (i.e., the maximum amount of money society is willing to pay for a unit of effect gained).[36] For spotting days, we used a maximum WTP threshold of €253 per one day reduction. This threshold was based on the value of eight hours of paid work given the average productivity costs per working hour for women in the Netherlands (i.e., €31.6 per hour). For QALYs, we used a WTP threshold of €20,000/QALY gained recommended by the Dutch Health Care Institute[25] and €23,420/QALY (equivalent of £20,000/QALY) recommended by the National Institute for Health and Clinical Excellence (NICE).[37]

Sensitivity analysis

Three sensitivity analyses (SA) were performed to assess the robustness of the results. SA1 consisted of a CEA including all women randomised (i.e., without excluding amenorrhoeic women) from both a societal and healthcare perspective for the QALY outcome. SA2 consisted of a complete case analysis from both a societal and healthcare perspective including only women without amenorrhea for both spotting days and QALYs. Finally, we performed per-protocol analyses for both outcomes from both a societal and healthcare perspective (SA3).

Patient and public involvement

The Dutch gynaecologic patients' association agreed upon the design of the study and the grant proposal for funding. They were not involved in outcome measures or recruitment, and they were not asked to give advice in the interpretation of the results. We will disseminate the study results to all participants, and to the public through popular science articles.

For peer review only - http://bmjopen.bmj.com/site/about/guidelines.xhtml

Results

Participants

In total, 2292 women undergoing a first CS were included. Of them, 1144 participants were randomised to single-layer and 1148 participants to double-layer closure of the uterine incision. In total, 672 women reported amenorrhoea (n=331 in control and n=341 in intervention group), resulting in 1620 women reported having menstrual blood loss over nine months of follow-up and were included in the main analyses (n=813 in control and n=807 in intervention group) [Figure 1]. Of all women included in the main analysis (n=1620), 95% had completed follow-up data for spotting days (n=1544, 774 in control group and 770 in intervention group). Within the total group (n=2292), complete follow-up data was available for 74% of QALYs (n=1696, 851 in control group and 845 in intervention group), for 72% of total healthcare costs (n=1653, 823 in control group and 830 in intervention group), and for 58% of total societal costs (n=1335, 671 in control group and 664 in intervention group)[Figure 1]. At baseline, no meaningful differences were found between both groups (Table 1). Participants with complete follow-up were more likely to be nulliparous and to have a higher education level compared to participants without complete follow-up.

Effectiveness

There were no statistically significant differences between groups in spotting days (mean difference -0.032, 95% CI, -0.342, 0.279) and QALYs (mean difference -0.004, 95% CI, -0.013; 0.005) at nine months follow-up (Table 2).

Costs

The main contributors to total societal costs in both groups were lost productivity costs (€4588 in control group and €4857 in intervention group) and healthcare costs (€742 in control group and €733 in intervention group, Table 2). Costs associated with absenteeism from unpaid work due to sickness

For peer review only - http://bmjopen.bmj.com/site/about/guidelines.xhtml

(i.e., costs related to getting help to perform household tasks, childcare, voluntary work) was the largest contributor to lost productivity costs in both groups (€3503 in control group and €3798 in intervention group). Secondary care costs was the largest contributor to total healthcare costs in both groups (€397 in control, group and €320 in intervention group). During follow-up, there were no statistically significant differences between groups in total healthcare costs (€-9, 95% CI, -247; 143) and total societal costs (€303, 95% CI, -413; 1035, Table 2). Presenteeism costs were statistically significantly lower in the intervention group compared to the control group (€-78, 95% CI, -156; -3, Table 2).

Cost-effectiveness analysis

From a societal perspective, most bootstrapped cost-effect pairs (45%) were in the North-East quadrant of the CE-plane for spotting days (Table 3, Figure 2 [1A]). The probability that double-layer is cost-effective compared to single-layer closure was 0.23 at a WTP of €0/spotting day less and 0.24 at €253/spotting day less (Figure 2 [2A]). For QALYs, most of the bootstrapped cost-effect pairs (65%) was in the North-West quadrant of the CE-plane (Table 3, Figure 2 [1B]). The probability of double-layer closure being cost-effective at both the Dutch WTP threshold of €20,000/QALY gained, and the UK WTP threshold of €23,420/QALY gained, was 0.19 from a societal perspective (Figure 2 [2B], supplementary table D).

From a healthcare perspective, bootstrapped cost-effect pairs were equally distributed among the Eastern and Western quadrants of the CE-plane for spotting days (Table 3, Figure 2 [1C]). This shows that uncertainty around costs and effects is large. The CEA curve presented in figure 2 [2C] shows that if the WTP for one spotting day less is €0, the probability of double-layer closure being cost-effective in comparison with single-layer closure was 0.53. This probability increases to 0.56 if the WTP is €253/spotting day less (Supplementary table S3). For QALYs, from a healthcare perspective, most of bootstrapped cost-effect pairs (42%) were in the North-West quadrant of the CE-plane (Table 3, Figure 2 [1D]). The probability that double-layer closure is cost-effective compared to single-layer closure at

For peer review only - http://bmjopen.bmj.com/site/about/guidelines.xhtml

the Dutch and UK WTP threshold (€20,000 and €23,420/QALY gained, respectively) is 0.32 and 0.30, respectively (Table 3, Figure 2 [1D]).

The results of the sensitivity analyses were similar to those of the main analysis (Table 3).

For peer review only

For peer review only - http://bmjopen.bmj.com/site/about/guidelines.xhtml

Discussion

The results of this trial-based economic evaluation showed that double-layer uterine closure after a first CS did not significantly decrease spotting days nor improve QALYs compared to single-layer at nine months follow-up. In addition, total healthcare costs and societal costs related to double-layer closure did not significantly differ from single-layer closure. Low probabilities of double-layer closure being cost-effective in comparison with single-layer closure were found for all relevant willingness-to-pay thresholds. Therefore, double-layer closure was not considered cost-effective compared to single-layer closure after a first CS from a societal and a healthcare perspective.

For peer review only

Comparison with previous studies

The results of this economic evaluation are not in line with our hypothesis, which was based on previously conducted observational studies[6 7 38] and meta-analyses.[13] These showed that single-layer closure resulted in thinner residual myometrium and a higher proportion of large niches than double-layer closure. These sonographic findings, or surrogates, were suggested to lead to more postmenstrual spotting and, therefore, higher related costs. Although double-layer closure resulted in increased CS costs, these costs were neutralised by higher secondary care and presenteeism costs in the single-layer closure group, resulting in no overall difference in total health care costs or total societal costs. It is unclear how to explain the significantly lower presenteeism costs in the double-layer closure group, especially considering the similar estimates for all other cost categories. Since we did not correct for multiple testing, this statistically significant finding may be the result of chance.

To the best of our knowledge, this is the first study evaluating the cost-effectiveness of double-layer closure in comparison with single-layer closure after a CS. The largest study (n=15,935) on this topic mentioned possible cost savings but no cost-effectiveness analysis was performed.[39] The second largest study (n=3033) comparing single- versus double-layer closure in a factorial RCT hypothesised on a possible reduction in costs in their study protocol, since a CS is conducted so frequently that *'any difference in morbidity is likely to have significant cost and community effects'*. The authors found a

difference in operative time, though they did not discuss costs in the 2010 publication, and it is unlikely that a cost-effectiveness analysis is going to be performed.[40]

Based on the current and previous studies[41 42], we recommend to leave the choice of uterine closure technique with the preference of the surgeon. Previous studies reported only short-term maternal outcomes in the first few weeks after CS.[40-42] We confirmed these findings using a follow-up of nine months after CS. In addition, we showed that there is no difference in costs between the two types of closure. Our three-years follow-up results will show whether double-layer is superior compared to single-layer closure with regard to long-term outcomes. These outcomes include fertility outcomes, pregnancy complications and mode of delivery, as well as safety outcomes such as uterine dehiscence or rupture and related neonatal and maternal morbidity. In addition, a cost-effectiveness analysis for long-term outcomes will be performed as well. When superiority cannot be shown on the long-term either, guidelines should recommend to leave the uterine closure technique regarding single- versus double-layer up to the preference of the performing surgeon.

Strengths and limitations

This study was performed alongside a large multicenter randomised controlled superiority trial, which is considered the best vehicle for economic evaluations because it allows the prospective collection of cost and effect data and the use of patient level information for drawing inferences about additional costs and benefits of interventions.[15] Additionally, the CEA was conducted from a societal perspective meaning that all relevant costs for decision making (i.e., intervention, healthcare utilization, informal care and lost productivity costs) were included in the analysis.[20]

However, one of the limitations of this study was that the cost questionnaires included retrospective self-reported questions over a 3-month and 6-month period, which may have caused recall bias. Nevertheless, we assume that this bias is equally distributed across the two groups and does, therefore, does not impact the difference between groups. Although there is no gold standard for

For peer review only

measuring lost productivity costs, we used a standardised instrument[27], which is considered best practice currently.[43] Another limitation is that generalizability of the results to healthcare systems in other countries may be limited, as they may adopt different usual practices and have different payment systems.[44]

Future research

It is important to realise that we have only evaluated the aspect of single-versus double-layer closure of the uterine incision in our trial. A CS consists of multiple steps and other aspects of the surgical technique used to perform a CS may also affect clinical outcomes and costs, and should therefore be subject to future research. Examples of uterine incision and repair are the level of hysterotomy (above or below the plica vesicouterina)[45] and inclusion or exclusion of the endometrium in the uterine suture.

Conclusions and policy implications

In conclusion, double-layer uterine closure is not cost-effective compared to single-layer uterine closure from both a societal and healthcare perspective. Thus, from a cost-effectiveness point of view, there is no reason to advocate double-layer over single-layer uterine closure. Long-term follow-up will show whether guidelines should be adapted based on obstetric and reproductive outcomes of double-layer closure compared to single-layer closure.

For peer review only - http://bmjopen.bmj.com/site/about/guidelines.xhtml

Acknowledgments

This study was performed with funding from ZonMw: The Netherlands Organisation for Health Research and Development (project number 843002605). We would like to thank all participants of the 2Close study. Additionally, we would like to thank the Departments of Obstetrics and Gynaecology of all participating hospitals, with special thanks to all research nurses and research midwives for their contribution in data collection.

The 2Close study group: we thank all collaborators from the participating hospitals for their effort, and a full list of collaborators is provided in the supplementary appendix.

Footnotes

Author Contributions: SIS collected data, interpreted data and drafted the report. AJB and MEA analysed and interpreted data and drafted the report. LFvdV designed the study, collected data and revised the first draft of the report critically. CJMdG designed the study and revised the first draft of the report critically. JEB designed the study, interpreted data and participated in drafting and revising the report. JAFH designed the study, was principle investigator, interpreted the data and participated in drafting and revising the report. All authors approved the final version of the manuscript.

Funding: This study was performed with funding from ZonMw: The Netherlands Organisation for Health Research and Development (project number 843002605). The funder of the study approved the study protocol. The funder had no role in study design, data collection, data analysis, data interpretation, or writing of the manuscript. The corresponding author had full access to all the data in the study and had final responsibility for the decision to submit for publication.

Competing interests: JAFH received grants from ZonMw, during the conduct of the study; and reports grants from Samsung, grants from PlantTec Medical GmbH, and received a fee from Olympus, all

For peer review only

For peer review only - http://bmjopen.bmj.com/site/about/guidelines.xhtml

outside the submitted work. CJMdG received a grant from ZonMw outside the submitted work. The other authors declare no competing interests.

Ethical approval: The study was approved by the Institutional Review Board (IRB) of Amsterdam UMC – location VU University medical centre in December 2015 (registration number 2015.462) and by the boards of all participating hospitals before start of inclusion. No substantial changes were made to the protocol after commencement of the trial. All participants provided written informed consent before taking part in the study.

For peer review only

Data sharing: De-identified individual participant data collected during the 2Close trial will be shared at one year after publication of the long-term results on request (j.huirne@amsterdamumc.nl). Approval of a proposal will be necessary before data will be shared. To gain access, requesters will need to sign an agreement form and confirm that data will be used for the purpose for which access was granted.

The corresponding and lead author (JAFH) affirms that the manuscript is an honest, accurate, and transparent account of the study being reported; no important aspects of the study have been omitted; any discrepancies from the study as originally planned in the registered study protocol have been explained. The corresponding author (JAFH) had the final responsibility for the decision to submit for publication.

This is an open access article distributed in accordance with the Creative Commons Attribution Non Commercial (CC BY-NC 4.0) license, which permits others to distribute, remix, adapt, build upon this work non-commercially, and license their derivative works on different terms, provided the original work is properly cited and the use is non-commercial. See: http://creativecommons.org/licenses/by-nc/4.0.

References

1. Boerma T, Ronsmans C, Melesse DY, et al. Global epidemiology of use of and disparities in caesarean sections. *Lancet* 2018;392(10155):1341-48. doi: 10.1016/s0140-6736(18)31928-7 [published Online First: 2018/10/17]
2. Betran AP, Ye J, Moller AB, et al. The Increasing Trend in Caesarean Section Rates: Global, Regional and National Estimates: 1990-2014. *PLoS One* 2016;11(2):e0148343. doi: 10.1371/journal.pone.0148343 [published Online First: 2016/02/06]
3. Marshall NE, Fu R, Guise JM. Impact of multiple cesarean deliveries on maternal morbidity: a systematic review. *Am J Obstet Gynecol* 2011;205(3):262.e1-8. doi: 10.1016/j.ajog.2011.06.035 [published Online First: 2011/11/11]
4. Roberge S, Boutin A, Chaillet N, et al. Systematic review of cesarean scar assessment in the nonpregnant state: imaging techniques and uterine scar defect. *Am J Perinatol* 2012;29(6):465-71. doi: 10.1055/s-0032-1304829 [published Online First: 2012/03/09]
5. Bij de Vaate AJ, van der Voet LF, Naji O, et al. Prevalence, potential risk factors for development and symptoms related to the presence of uterine niches following Cesarean section: systematic review. *Ultrasound Obstet Gynecol* 2014;43(4):372-82. doi: 10.1002/uog.13199 [published Online First: 2013/09/03]
6. Bij de Vaate AJ, Brolmann HA, van der Voet LF, et al. Ultrasound evaluation of the Cesarean scar: relation between a niche and postmenstrual spotting. *Ultrasound Obstet Gynecol* 2011;37(1):93-9. doi: 10.1002/uog.8864 [published Online First: 2010/10/30]
7. van der Voet LF, Bij de Vaate AM, Veersema S, et al. Long-term complications of caesarean section. The niche in the scar: a prospective cohort study on niche prevalence and its relation to abnormal uterine bleeding. *BJOG* 2014;121(2):236-44. doi: 10.1111/1471-0528.12542 [published Online First: 2014/01/01]
8. van der Voet LF, Vervoort AJ, Veersema S, et al. Minimally invasive therapy for gynaecological symptoms related to a niche in the caesarean scar: a systematic review. *BJOG* 2014;121(2):145-56. doi: 10.1111/1471-0528.12537 [published Online First: 2014/01/01]
9. Eurostat. Surgical operations and procedures statistics - statistics explained. . 03 Oct 2019. https://ec.europa.eu/eurostat/statisticsexplained/ (accessed 18 Nov 2019).
10. Caughey AB, Wood SL, Macones GA, et al. Guidelines for intraoperative care in cesarean delivery: Enhanced Recovery After Surgery Society Recommendations (Part 2). *Am J Obstet Gynecol* 2018;219(6):533-44. doi: 10.1016/j.ajog.2018.08.006 [published Online First: 2018/08/18]
11. NICE. Caesarean section - clinical guideline. . 01 Nov 2011. https://www.nice.org.uk/guidance/cg132 (accessed 19 Nov 2019).
12. Roberge S, Demers S, Berghella V, et al. Impact of single- vs double-layer closure on adverse outcomes and uterine scar defect: a systematic review and metaanalysis. *Am J Obstet Gynecol* 2014;211(5):453-60. doi: 10.1016/j.ajog.2014.06.014 [published Online First: 2014/06/10]
13. Stegwee SI, Jordans I, van der Voet LF, et al. Uterine caesarean closure techniques affect ultrasound findings and maternal outcomes: a systematic review and meta-analysis. *BJOG* 2018;125(9):1097-108. doi: 10.1111/1471-0528.15048 [published Online First: 2017/12/08]
14. Sevket O, Ates S, Molla T, et al. Hydrosonographic assessment of the effects of 2 different suturing techniques on healing of the uterine scar after cesarean delivery. *Int J Gynaecol Obstet* 2014;125(3):219-22. doi: 10.1016/j.ijgo.2013.11.013 [published Online First: 2014/04/01]
15. Ramsey SD, Willke RJ, Glick H, et al. Cost-effectiveness analysis alongside clinical trials II-An ISPOR Good Research Practices Task Force report. *Value Health* 2015;18(2):161-72. doi: 10.1016/j.jval.2015.02.001 [published Online First: 2015/03/17]
16. Stegwee SI, Jordans IPM, van der Voet LF, et al. Single- versus double-layer closure of the caesarean (uterine) scar in the prevention of gynaecological symptoms in relation to niche

For peer review only - http://bmjopen.bmj.com/site/about/guidelines.xhtml

development - the 2Close study: a multicentre randomised controlled trial. *BMC Pregnancy Childbirth* 2019;19(1):85. doi: 10.1186/s12884-019-2221-y [published Online First: 2019/03/06]

17. Husereau D, Drummond M, Petrou S, et al. Consolidated Health Economic Evaluation Reporting Standards (CHEERS) statement. *BMJ* 2013;346:f1049. doi: 10.1136/bmj.f1049 [published Online First: 2013/03/27]
18. Jordans IPM, de Leeuw RA, Stegwee SI, et al. Sonographic examination of uterine niche in non-pregnant women: a modified Delphi procedure. *Ultrasound Obstet Gynecol* 2019;53(1):107-15. doi: 10.1002/uog.19049 [published Online First: 2018/03/15]
19. Whitehead SJ, Ali S. Health outcomes in economic evaluation: the QALY and utilities. *Br Med Bull* 2010;96:5-21. doi: 10.1093/bmb/ldq033 [published Online First: 2010/11/03]
20. van Dongen JM, van Wier MF, Tompa E, et al. Trial-based economic evaluations in occupational health: principles, methods, and recommendations. *J Occup Environ Med* 2014;56(6):563-72. doi: 10.1097/jom.0000000000000165 [published Online First: 2014/05/24]
21. Gray A, Clarke PM, Wolstenholme JL, et al. Applied Methods of Cost-effectiveness Analysis in Healthcare: Oxford University Press. http://econpapers.repec.org/bookchap/oxpobooks/9780199227280.htm (accessed Oct 28, 2019), 2010.
22. Herdman M, Gudex C, Lloyd A, et al. Development and preliminary testing of the new five-level version of EQ-5D (EQ-5D-5L). *Qual Life Res* 2011;20(10):1727-36. doi: 10.1007/s11136-011-9903-x [published Online First: 2011/04/12]
23. Versteegh MM, Vermeulen KM, Evers SMAA, et al. Dutch Tariff for the Five-Level Version of EQ-5D. *Value Health* 2016;19(4):343-52. doi: 10.1016/j.jval.2016.01.003 [published Online First: 2016/06/22]
24. Bouwmans C, Hakkaart-van Roijen L, Koopmanschap M, et al. Manual iMTA medical cost questionnaire (iMCQ). *Rotterdam: iMTA, Erasmus Universiteit Rotterdam* 2013
25. Hakkaart - van Roijen L, van der Linden N, Bouwmans CAM, et al. Costing manual: Methodology of costing research and reference prices for economic evaluations in healthcare. In: Volksgezondheid WeS, ed. Nederland: Zorginstituut Nederland, 2016.
26. VWS. Medicijnkosten - Zorginstituut Nederland - Ministerie van Volksgezondheid, Welzijn en Sport, 2018.
27. Bouwmans C, Krol M, Severens H, et al. The iMTA Productivity Cost Questionnaire: A Standardized Instrument for Measuring and Valuing Health-Related Productivity Losses. *Value Health* 2015;18(6):753-8. doi: 10.1016/j.jval.2015.05.009 [published Online First: 2015/09/28]
28. Koopmanschap MA, Rutten FF, van Ineveld BM, et al. The friction cost method for measuring indirect costs of disease. *J Health Econ* 1995;14(2):171-89. [published Online First: 1995/05/08]
29. Krol M, Brouwer W. How to estimate productivity costs in economic evaluations. *Pharmacoeconomics* 2014;32(4):335-44. doi: 10.1007/s40273-014-0132-3 [published Online First: 2014/02/08]
30. White IR, Royston P, Wood AM. Multiple imputation using chained equations: Issues and guidance for practice. *Stat Med* 2011;30(4):377-99. doi: 10.1002/sim.4067 [published Online First: 2011/01/13]
31. Madley-Dowd P, Hughes R, Tilling K, et al. The proportion of missing data should not be used to guide decisions on multiple imputation. *J Clin Epidemiol* 2019;110:63-73. doi: 10.1016/j.jclinepi.2019.02.016 [published Online First: 2019/03/18]
32. Rubin DB. Multiple Imputation for Nonresponse in Surveys: John Wiley & Sons 2004.
33. Willan AR, Briggs AH, Hoch JS. Regression methods for covariate adjustment and subgroup analysis for non-censored cost-effectiveness data. *Health Econ* 2004;13(5):461-75. doi: 10.1002/hec.843 [published Online First: 2004/05/06]

For peer review only

For peer review only - http://bmjopen.bmj.com/site/about/guidelines.xhtml

34. Twisk JWR. Applied Longitudinal Data Analysis for Epidemiology: A Practical Guide: Cambridge University Press 2013.
35. Black WC. The CE plane: a graphic representation of cost-effectiveness. *Med Decis Making* 1990;10(3):212-4. doi: 10.1177/0272989x9001000308 [published Online First: 1990/07/01]
36. Fenwick E, O'Brien BJ, Briggs A. Cost-effectiveness acceptability curves--facts, fallacies and frequently asked questions. *Health Econ* 2004;13(5):405-15. doi: 10.1002/hec.903 [published Online First: 2004/05/06]
37. McCabe C, Claxton K, Culyer AJ. The NICE cost-effectiveness threshold: what it is and what that means. *Pharmacoeconomics* 2008;26(9):733-44. doi: 10.2165/00019053-200826090-00004 [published Online First: 2008/09/05]
38. Hayakawa H, Itakura A, Mitsui T, et al. Methods for myometrium closure and other factors impacting effects on cesarean section scars of the uterine segment detected by the ultrasonography. *Acta Obstet Gynecol Scand* 2006;85(4):429-34. doi: 10.1080/00016340500430436 [published Online First: 2006/04/14]
39. Abalos E, Addo V, Brocklehurst P, et al. Caesarean section surgical techniques: 3 year follow-up of the CORONIS fractional, factorial, unmasked, randomised controlled trial. *Lancet* 2016;388(10039):62-72. doi: 10.1016/s0140-6736(16)00204-x [published Online First: 2016/05/09]
40. The CAESAR study collaborative group. Caesarean section surgical techniques: a randomised factorial trial (CAESAR). *BJOG* 2010;117(11):1366-76. doi: 10.1111/j.1471-0528.2010.02686.x [published Online First: 2010/09/16]
41. Dodd JM, Anderson ER, Gates S, et al. Surgical techniques for uterine incision and uterine closure at the time of caesarean section. *Cochrane Database Syst Rev* 2014(7):CD004732. doi: 10.1002/14651858.CD004732.pub3 [published Online First: 2014/07/23]
42. Abalos E, Addo V, Brocklehurst P, et al. Caesarean section surgical techniques (CORONIS): a fractional, factorial, unmasked, randomised controlled trial. *Lancet* 2013;382(9888):234-48. doi: 10.1016/s0140-6736(13)60441-9 [published Online First: 2013/06/01]
43. Versteegh M, Knies S, Brouwer W. From Good to Better: New Dutch Guidelines for Economic Evaluations in Healthcare. *Pharmacoeconomics* 2016;34(11):1071-74. doi: 10.1007/s40273-016-0431-y [published Online First: 2016/10/22]
44. Drummond M, Barbieri M, Cook J, et al. Transferability of economic evaluations across jurisdictions: ISPOR Good Research Practices Task Force report. *Value Health* 2009;12(4):409-18. doi: 10.1111/j.1524-4733.2008.00489.x [published Online First: 2009/11/11]
45. Vikhareva O, Rickle GS, Lavesson T, et al. Hysterotomy level at Cesarean section and occurrence of large scar defects: a randomized single-blind trial. *Ultrasound Obstet Gynecol* 2019;53(4):438-42. doi: 10.1002/uog.20184 [published Online First: 2018/11/30]

For peer review only - http://bmjopen.bmj.com/site/about/guidelines.xhtml

Figures

Figure 1: Trial profile

*Logistical reasons, computer randomisation issues, passing through the allocated method to operating gynaecologist, or participant not traceable after randomisation.

Figure 2: Cost-effectiveness planes and cost acceptability curves from a societal and healthcare perspective

[1] Cost-effectiveness planes (CE-planes) showing the incremental cost-effectiveness ratio point estimate (red dot) and the distribution of the 5,000 replications of the bootstrapped cost-effective pairs (blue dots). [2] Cost-effectiveness acceptability curves (CEACs) indicating the probability of cost-effectiveness (y-axis) for different willingness-to-pay (WTP) thresholds per unit of effect gained (x-axis).
[1A] CE-plane for spotting days and societal costs showing that 45% of the bootstrapped cost-effect pairs were in the North-East quadrant, where the intervention was more costly and more effective compared to control. [2A] CEAC for spotting days and societal costs indicating a probability of the intervention being cost-effective around 0.2 for different WTP thresholds.
[1B] CE-plane for QALYs and societal costs showing that 65% of the bootstrapped cost-effect pairs were in the North-West, where the intervention was more costly and less effective compared to control. [2B] CEACs for QALY and societal costs indicating a probability of the intervention being cost-effective around 0.2 for different WTP thresholds.
[1C] CE-plane for spotting days and healthcare costs showing that bootstrapped cost-effect pairs were equally distributed among the Eastern and Western quadrants. This means a large uncertainty around costs and effects. [2C] CEAC for spotting days and healthcare costs indicating a probability of the intervention being cost-effective around 0.5 for different WTP thresholds.
[1D] CE-plane for QALYs and healthcare costs showing that 42% of the bootstrapped cost-effect pairs were in the North-West, where the intervention was more costly and less effective compared to control. [2D] CEACs for QALY and healthcare costs indicating a probability of the intervention being cost-effective ranging from 0.5 at zero to WTP to around 0.2 at a WTP of €50,000/QALY gained

For peer review only - http://bmjopen.bmj.com/site/about/guidelines.xhtml

614 # Tables

615 **Table 1: Baseline characteristics of women assigned to the intervention or control group**

	Single-layer (N=1144)	Double-layer (N=1148)
Age, years	32.0 (4.7), n=1013	32.1 (4.6), n=1015
Level of education		
Low	68 (6.7), n=1013	70 (6.9), n=1015
Middle	349 (34.4), n=1013	329 (32.4), n=1015
High	591 (58.3), n=1013	606 (59.7), n=1015
Nulliparous women	764 (75.4), n=1013	764 (75.3), n=1014
BMI kg/m^2	26.4 (4.6), n=1013	26.1 (4.8), n=1014
Smoking habit	61 (6.1), n=1013	48 (4.7), n=1015
Hypertension	191 (18.9), n=1009	174 (17.2), n=1011
Diabetes mellitus	113 (11.2), n=1011	90 (8.9), n=1011
Gestational age	38.5 (2.5), n=1011	38.5 (2.5), n=1015
Previous miscarriage	335 (33.1), n=1013	312 (30.8), n=1014
Previous ectopic pregnancy	13 (1.3), n=1013	18 (1.8), n=1015

616 Data are mean (SD), or n (%). N is equal to the total number of patients in the group, n= number of
617 participants with completed data. BMI, body mass index.

For peer review only

For peer review only - http://bmjopen.bmj.com/site/about/guidelines.xhtml

Table 2: Multiply imputed mean effects and costs by group and mean difference at nine months follow-up

	Single-layer (n=1144)	Double-layer (n=1148)	Mean difference* (95% CI)
**Effects**			
Spotting days	1.42 (0.10)	1.39 (0.10)	-0.032 (-0.342 to 0.279)
QALYs gained	0.663 (0.003)	0.659 (0.003)	-0.004 (-0.013 to 0.005)
**Costs**			
Intervention costs†	0	76 (0.31)	76 (75 to 76)
Primary care costs	257 (14)	250 (15)	-7 (-48 to 34)
Secondary care costs	397 (101)	320 (38)	-77 (-267 to 39)
Medication costs	87 (13)	87 (24)	0 (-88 to 69)
Total healthcare costs‡	742 (104)	733 (51)	-9 (-247 to 143)
Informal care costs	78 (15)	121 (25)	43 (-9 to 121)
Absenteeism costs at paid work	624 (38)	677 (40)	53 (-53 to 161)
Absenteeism costs at unpaid work	3503 (191)	3798 (231)	295 (-293 to 910)
Presenteeism costs	460 (27)	382 (23)	-78 (-156 to -3)
Total lost productivity costs	4588 (210)	4857 (245)	269 (-372 to 930)
**Total societal costs§**	5408 (276)	5711 (304)	303 (-413 to 1035)

Data are mean (SE). CI=confidence interval, QALY=quality-adjusted life-years. Multiple imputation model consisted of age, education level, parity, body mass index, smoking habit, hypertensive disorder, diabetic status, gestational complications, gestational age, previous miscarriage or ectopic pregnancies and self-reported menstrual blood loss. *Cost and effect differences at nine months follow-up were estimated using seemingly unrelated regression analyses.[33] †Additional intervention costs to perform double-layer, excluding the average costs for performing a caesarean section (€5360,-). ‡The sum of intervention, primary care, secondary care and medication costs. §The sum of total healthcare costs, informal care costs, and lost productivity costs.

For peer review only

For peer review only - http://bmjopen.bmj.com/site/about/guidelines.xhtml

Table 3: Results of the cost-effectiveness analysis

Effect outcome*	Cost difference, € (95% CI)	Effect difference (95% CI)	ICER €/ effect gained	Distribution of the cost-effectiveness plane			
				North-East	South-East	South-West	North-West
**Main analysis – Societal perspective**							
Spotting days	303 (-413 to 1035)	0.032 (-0.279 to 0.342)	9560	45%	13%	10%	32%
QALY	303 (-413 to 1035)	-0.004 (-0.013 to 0.005)	-75100	12%	7%	16%	65%
**Main analysis – Healthcare perspective**							
Spotting days	-9 (-250 to 144)	0.032 (-0.279 to 0.342)	-285	30%	28%	20%	22%
QALY	-9 (-250 to 144)	-0.004 (-0.013 to 0.005)	2235	9%	10%	39%	42%
**SA1 – including all women randomised - Societal perspective**							
QALY	137 (-716 to 861)	-0.005 (-0.013 to 0.003)	-28981	6%	7%	30%	57%
**SA1 – including all women randomised - Healthcare perspective**							
QALY	-182 (-1020 to 89)	-0.005 (-0.013 to 0.003)	38447	3%	10%	65%	22%
**SA2 – complete case analysis – Societal perspective**							
Spotting days	380 (-451 to 1273)	0.149 (-0.452 to 0.138)	2554	74%	14%	2%	10%
QALY	397 (-427 to 1275)	-0.006 (-0.017 to 0.003)	-64515	22%	4%	12%	62%
**SA2 - complete case analysis – Healthcare perspective**							
Spotting days	-9 (-251 to 174)	0.186 (-0.486 to 0.103)	-46	54%	37%	4%	5%
QALY	1 (-255 to 177)	-0.007 (-0.016 to 0.002)	-80	23%	4%	34%	39%
**SA3 – per protocol analysis - Societal perspective**							
Spotting days	102 (-611 to 837)	0.023 (-0.287 to 0.334)	4398	34%	22%	18%	26%
QALY	102 (-611 to 837)	-0.003 (-0.012 to 0.006)	-31609	11%	13%	27%	49%
**SA3 – per protocol analysis – Healthcare perspective**							
Spotting days	-28 (-261 to 124)	0.023 (-0.287 to 0.334)	-1230	24%	32%	25%	19%
QALY	-28 (-261 to 124)	-0.003 (-0.012 to 0.006)	8838	9%	15%	42%	34%

Data are mean (95% CI). *The effect outcome "Spotting days" was multiplied by -1 in the cost-effectiveness analysis to keep the CE-plane interpretable. CI=confidence interval, ICER=incremental cost-effectiveness ratio, QALY=quality-adjusted life-year, SA=sensitivity analysis.
SA1: CEA from a societal and healthcare perspective for QALY, including all women randomised in the study after multiple imputation (i.e., without excluding amenorrhoeic women, n=2292)[Supplementary Table S2].
SA2: CEA from a societal perspective using complete cases for spotting days and total societal costs (total=1075, control n=554, intervention n=521) including only women without amenorrhea (n=1620).
SA2: CEA from a societal perspective using complete cases for QALY total societal costs (total=1057, control n=541, intervention n=516) including only women without amenorrhea (n=1620).
SA2: CEA from a healthcare perspective using complete cases for spotting days total healthcare costs (total=1315, control n=662, intervention n=653) including only women without amenorrhea (n=1620).
SA2: CEA from a healthcare perspective using complete cases for QALY and total healthcare costs (total=1310, control n=657, intervention n=653) including only women without amenorrhea (n=1620).
SA3: per-protocol analysis for spotting days and QALY from a societal perspective (total=1620, control n=828, intervention n=792) including only women without amenorrhea (n=1620).
SA3: per-protocol analysis for spotting days and QALY from a healthcare perspective (total=1620, control n=828, intervention n=792) including only women without amenorrhea (n=1620).

For peer review only - http://bmjopen.bmj.com/site/about/guidelines.xhtml

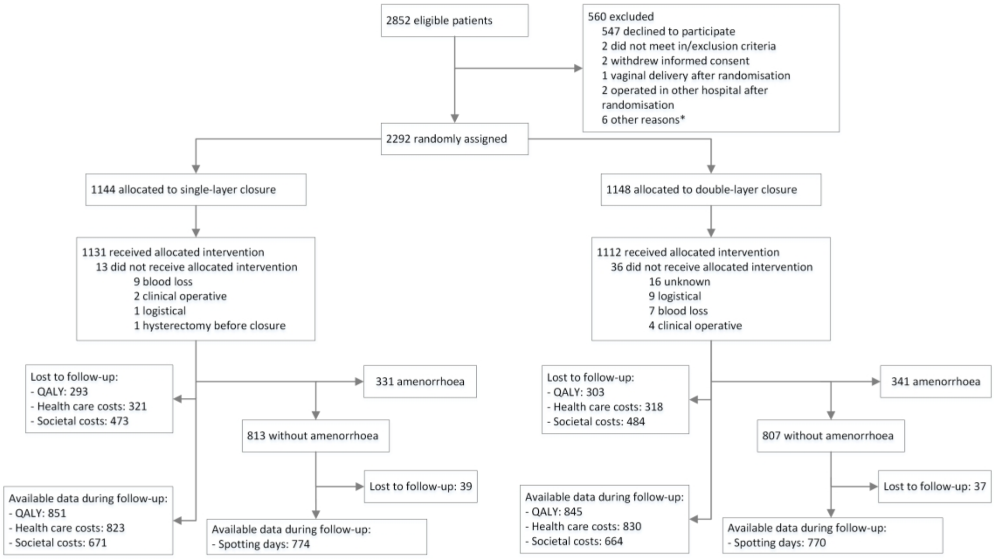

Figure 1: Trial profile

*Logistical reasons, computer randomisation issues, passing through the allocated method to operating gynaecologist, or participant not traceable after randomisation.

287x161mm (300 x 300 DPI)

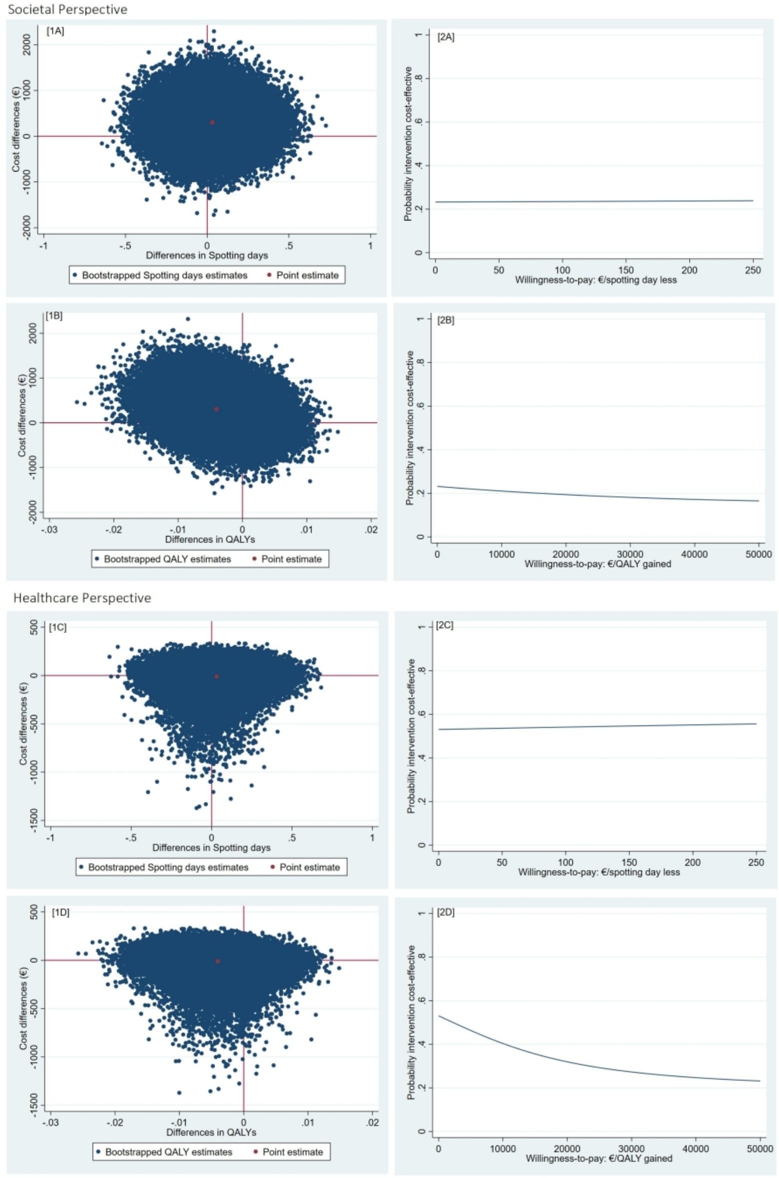

Figure 2: Cost-effectiveness planes and cost acceptability curves from a societal and healthcare perspective

[1] Cost-effectiveness planes (CE-planes) showing the incremental cost-effectiveness ratio point estimate (red dot) and the distribution of the 5,000 replications of the bootstrapped cost-effective pairs (blue dots). [2] Cost-effectiveness acceptability curves (CEACs) indicating the probability of cost-effectiveness (y-axis) for different willingness-to-pay (WTP) thresholds per unit of effect gained (x-axis).
[1A] CE-plane for spotting days and societal costs showing that 45% of the bootstrapped cost-effect pairs were in the North-East quadrant, where the intervention was more costly and more effective compared to control. [2A] CEAC for spotting days and societal costs indicating a probability of the intervention being cost-effective around 0.2 for different WTP thresholds.
[1B] CE-plane for QALYs and societal costs showing that 65% of the bootstrapped cost-effect pairs were in the North-West, where the intervention was more costly and less effective compared to control. [2B] CEACs for QALY and societal costs indicating a probability of the intervention being cost-effective around 0.2 for different WTP thresholds.

[1C] CE-plane for spotting days and healthcare costs showing that bootstrapped cost-effect pairs were equally distributed among the Eastern and Western quadrants. This means a large uncertainty around costs and effects. [2C] CEAC for spotting days and healthcare costs indicating a probability of the intervention being cost-effective around 0.5 for different WTP thresholds.
[1D] CE-plane for QALYs and healthcare costs showing that 42% of the bootstrapped cost-effect pairs were in the North-West, where the intervention was more costly and less effective compared to control. [2D] CEACs for QALY and healthcare costs indicating a probability of the intervention being cost-effective ranging from 0.5 at zero to WTP to around 0.2 at a WTP of €50,000/QALY gained

525x801mm (120 x 120 DPI)

Stegwee et al. BMC Pregnancy and Childbirth (2019) 19:85
https://doi.org/10.1186/s12884-019-2221-y

BMC Pregnancy and Childbirth

STUDY PROTOCOL Open Access

Single- versus double-layer closure of the caesarean (uterine) scar in the prevention of gynaecological symptoms in relation to niche development – the 2Close study: a multicentre randomised controlled trial

S. I. Stegwee[1†], I. P. M. Jordans[1†], L. F. van der Voet[2], M. Y. Bongers[3,19], C. J. M. de Groot[1], C. B. Lambalk[1], R. A. de Leeuw[1], W. J. K. Hehenkamp[1], P. M. van de Ven[4], J. E. Bosmans[5], E. Pajkrt[6], E. A. Bakkum[7], C. M. Radder[8], M. Hemelaar[9], W. M. van Baal[10], H. Visser[11], J. O. E. H. van Laar[3], H. A. A. M. van Vliet[12], R. J. P. Rijnders[13], M. Sueters[14], C. A. H. Janssen[15], W. Hermes[16], A. H. Feitsma[17], K. Kapiteijn[18], H. C. J. Scheepers[19], J. Langenveld[20], K. de Boer[21], S. F. P. J. Coppus[22], D. H. Schippers[23], A. L. M. Oei[24], M. Kaplan[25], D. N. M. Papatsonis[26], L. H. M. de Vleeschouwer[27], E. van Beek[28], M. N. Bekker[29], A. J. M. Huisjes[30], W. J. Meijer[31], K. L. Deurloo[32], E. M. A. Boormans[33], H. W. F. van Eijndhoven[34] and J. A. F. Huirne[1*]

Abstract

Background: Double-layer compared to single-layer closure of the uterus after a caesarean section (CS) leads to a thicker myometrial layer at the site of the CS scar, also called residual myometrium thickness (RMT). It possibly decreases the development of a niche, which is an interruption of the myometrium at the site of the uterine scar. Thin RMT and a niche are associated with gynaecological symptoms, obstetric complications in a subsequent pregnancy and delivery and possibly with subfertility.

(Continued on next page)

* Correspondence: j.huirne@vumc.nl
†S. I. Stegwee and I. P. M. Jordans contributed equally to this work.
[1]Department of Obstetrics and Gynaecology, Research institutes 'Amsterdam Cardiovascular Sciences' and 'Amsterdam Reproduction and Development', Amsterdam UMC, Vrije Universiteit Amsterdam, De Boelelaan 1117, 1081 HV Amsterdam, Netherlands
Full list of author information is available at the end of the article

© The Author(s). 2019 **Open Access** This article is distributed under the terms of the Creative Commons Attribution 4.0 International License (http://creativecommons.org/licenses/by/4.0/), which permits unrestricted use, distribution, and reproduction in any medium, provided you give appropriate credit to the original author(s) and the source, provide a link to the Creative Commons license, and indicate if changes were made. The Creative Commons Public Domain Dedication waiver (http://creativecommons.org/publicdomain/zero/1.0/) applies to the data made available in this article, unless otherwise stated.

(Continued from previous page)

Methods: Women undergoing a first CS regardless of the gestational age will be asked to participate in this multicentre, double blinded randomised controlled trial (RCT). They will be randomised to single-layer closure or double-layer closure of the uterine incision. Single-layer closure (control group) is performed with a continuous running, unlocked suture, with or without endometrial saving technique. Double-layer closure (intervention group) is performed with the first layer in a continuous unlocked suture including the endometrial layer and the second layer is also continuous unlocked and imbricates the first. The primary outcome is the reported number of days with postmenstrual spotting during one menstrual cycle nine months after CS. Secondary outcomes include surgical data, ultrasound evaluation at three months, menstrual pattern, dysmenorrhea, quality of life, and sexual function at nine months. Structured transvaginal ultrasound (TVUS) evaluation is performed to assess the uterine scar and if necessary saline infusion sonohysterography (SIS) or gel instillation sonohysterography (GIS) will be added to the examination. Women and ultrasound examiners will be blinded for allocation. Reproductive outcomes at three years follow-up including fertility, mode of delivery and complications in subsequent deliveries will be studied as well. Analyses will be performed by intention to treat. 2290 women have to be randomised to show a reduction of 15% in the mean number of spotting days. Additionally, a cost-effectiveness analysis will be performed from a societal perspective.

Discussion: This RCT will provide insight in the outcomes of single- compared to double-layer closure technique after CS, including postmenstrual spotting and subfertility in relation to niche development measured by ultrasound.

Trial registration: Dutch Trial Register (NTR5480). Registered 29 October 2015.

Keywords: Caesarean section, Closure techniques, Long-term outcomes, Postmenstrual spotting, Niche, Quality of life, Fertility, Reproductive outcomes,

For peer review only

Background

Caesarean section (CS) rates have increased from 14.5 to 27.2% in the last two decades in the Western world. [1] In 2016, 26.664 CSs were performed in the Netherlands, being 16.0% of the total number of deliveries. [2] The increasing CS rate has stimulated an interest in the potential long-term morbidity of a CS scar, such as uterine rupture or malplacentation. [3–7] Other less severe, but more prevalent long-term symptoms are gynaecological symptoms and subfertility.

Only recently, gynaecological symptoms such as painful menstruations and postmenstrual spotting have been associated with CSs. [8–10] These symptoms are considered to be related to a niche, defined as *"an indentation at the site of the caesarean scar with a depth of at least 2 mm"*, visible on transvaginal ultrasound (TVUS). [11] Two cohort studies reported a strong association between postmenstrual spotting and a niche: odds ratio (OR) 3.1; 95% confidence interval (CI) 1.5–6.3 [8] and OR 5.5; 95% CI 1.1–26.5. [10] In these studies, a niche was observed in 50 to 60% of the women after a CS, using transvaginal ultrasound. [8, 10] Spotting was correlated to niche volume and inversely correlated to the residual myometrium thickness (RMT). [8, 10]

In addition to the gynaecological symptoms, a niche may influence fertility. A recent meta-analysis reported that a CS on average reduced the probability of subsequent pregnancy with 9% (relative risk (RR) 0.91; 95% CI 0.87–0.95) in comparison to a vaginal delivery. [12] None of the included studies in this meta-analysis evaluated the relation between subsequent fertility and the presence of a niche. One of the hypotheses is that intra-uterine fluid or cervical mucus or blood accumulation in the niche are expected to hamper the penetration of sperm cells or impair embryo implantation. [13] Long-term follow-up will facilitate the evaluation of the association between uterine closure, niche development, accumulation of intra-uterine fluid and subfertility.

In the last years, various therapies have been developed and implemented to treat niche related symptoms such as menstrual disorders. [14–18] Effectiveness of both hysteroscopic [19] and laparoscopic niche resection [15] have recently been published. Because both niche related symptoms and applied therapies lead to increases in medical consultations and costs, it seems to be more efficient to prevent niche development in the first place. Uterine closure technique of the CS scar has been proposed as an independent factor for niche development. [9] However, large randomised trials evaluating the effect of uterine closuring techniques on postmenstrual spotting or other gynaecological or reproductive outcomes in relation to niche development and thin residual myometrium are lacking, as well as cost-effectiveness evaluations.

In order to shorten surgery time and in the absence of significant differences in short-term outcomes [20, 21], most Dutch gynaecologists (92%) have replaced double-layer by single-layer closure after a CS, using

multifilament continuous unlocked sutures. Given the higher risk on myometrium loss and thus development of a thinner residual myometrium after single-layer closure [5, 22], we hypothesise that this method introduces a higher risk on postmenstrual spotting and possibly subfertility after a CS and that it can be prevented by applying double-layer unlocked closure.

Double-layer unlocked closure is considered safe, without a clinically relevant higher risk on short-term outcomes. [5, 23, 24]. Moreover, it results in a thicker residual myometrium, especially when unlocked sutures are applied. [5, 22, 24] Dysmenorrhea was reported more frequently after single-layer closure, but this was only studied in two RCTs and not always related to ultrasound findings such as myometrial thickness or niche presence. [24] Prevalence of uterine rupture seems to be similar after single- versus double-layer closure [5, 22, 24], but has neither been related to ultrasound findings and since it has a very low incidence, statistically significant differences are difficult to find. Since long-term outcomes such as gynaecological symptoms, fertility outcomes and results of subsequent pregnancies are studied infrequently, additional evidence is needed before a preference for either technique can be indicated.

Objective

Our primary objective is to determine the effectiveness of unlocked double-layer uterine closure compared to unlocked single-layer uterine closure in the prevention of niche related gynaecological symptoms nine months after a first CS. Secondary objectives are to assess niche prevalence measured by ultrasound at three months follow-up and to study both reproductive outcomes related to a subsequent pregnancy and gynaecological symptoms at three years follow-up. Additionally we aim to study the cost-effectiveness alongside the trial.

Methods/design

Design

This multicentre randomised controlled superiority trial will be performed in the Netherlands, in hospitals that collaborate within the Dutch Consortium for Healthcare Evaluation and Research in Obstetrics and Gynaecology (NVOG Consortium 2.0, www.zorgevaluatienederland.nl). Centres that participate are district, teaching or university hospitals in the Netherlands. A list of study sites is available in Additional file 1.

Participants and eligibility criteria

All women who undergo a first CS, planned or unplanned, will be asked to participate in the study. Other inclusion criteria are: sufficient command of the Dutch or English language, age ≥ 18 years and written informed consent. To prevent confounding effects on niche development during the study, we will exclude women with a previous CS. Other exclusion criteria are: inadequate possibility for counselling (e.g. indication for emergency CS without being informed about the study previously, women in severe pain without adequate therapy), previous major uterine surgery (e.g. laparoscopic or laparotomic fibroid resection, septum resection), women with known causes of menstrual disorders (e.g. cervical dysplasia, communicating hydrosalpinx, uterine anomaly or endocrine disorders disturbing ovulation), placenta increta or percreta during the current pregnancy or ≥ three foetuses during the current pregnancy.

Recruitment and randomisation

Eligible women will be asked by a gynaecologist, resident, clinical midwife or research nurse to participate in the trial when they undergo a planned CS. Eligible women who are planned to undergo a vaginal delivery will also be informed about this study during pregnancy in case they need an unplanned CS. Furthermore, women during induced labour and women receiving adequate therapy for pain during labour, will be asked to participate in case a CS is needed during labour for any indication.

When the decision of a CS is made and all selection criteria are met, women will be randomly allocated to single-layer (control group) or double-layer (intervention group) closure (1:1) (see Fig. 1). Randomisation will be performed using a web-based application ALEA 2.2 which displays a computer-generated random number, managed by the Clinical Research Unit of the Amsterdam UMC - location AMC. We will use a permuted block-design, stratified for recruiting centres and for planned or unplanned CS. All women that decline to participate will be registered anonymously in order to record the number and reason for refusal. Subjects who withdraw from this study will not be replaced.

Gynaecologists, residents, clinical midwives or research nurses enrol participants and assign them to the intervention. The CS will be performed by either a gynaecologist, a resident supervised by a gynaecologist or by a resident that is authorised to perform CSs without supervision. Participants and sonographers will be blinded for the closure technique. If operative reintervention after CS is needed and the gynaecologist that performs the reintervention needs to know the closure technique that the participant was assigned to, unblinding is possible through the logistic trial coordinator. We expect this situation to occur very infrequently.

Intervention (double-layer closure)

In both study arms, women will undergo a CS following a standard way with respect to mode of uterotomy,

For peer review only - http://bmjopen.bmj.com/site/about/guidelines.xhtml

correct approximations of the cutting edges and non-closure of the peritoneum. In the intervention arm, double-layer closure of the uterus will be performed using unlocked multifilament continuous running sutures for both layers and the endometrial layer will be included in the first layer (see Fig. 2). The second layer is a continuous running suture that imbricates the first layer. Since this is not the standard method for uterine closure in the Netherlands, a short online instruction film will be shown to all participating centres and surgeons prior to participation (see Additional file 2). Surgical outcomes will be registered after the procedure in the electronic case report form (eCRF).

Control group (single-layer closure)

The control group will receive usual closure technique of the uterus: a single-layer closure using unlocked continuous running multifilament sutures. The currently available evidence is inconclusive with respect to endometrial saving technique or not. Therefore, we decided that in our study surgeons are free to choose to close either full thickness (including the endometrium) or split

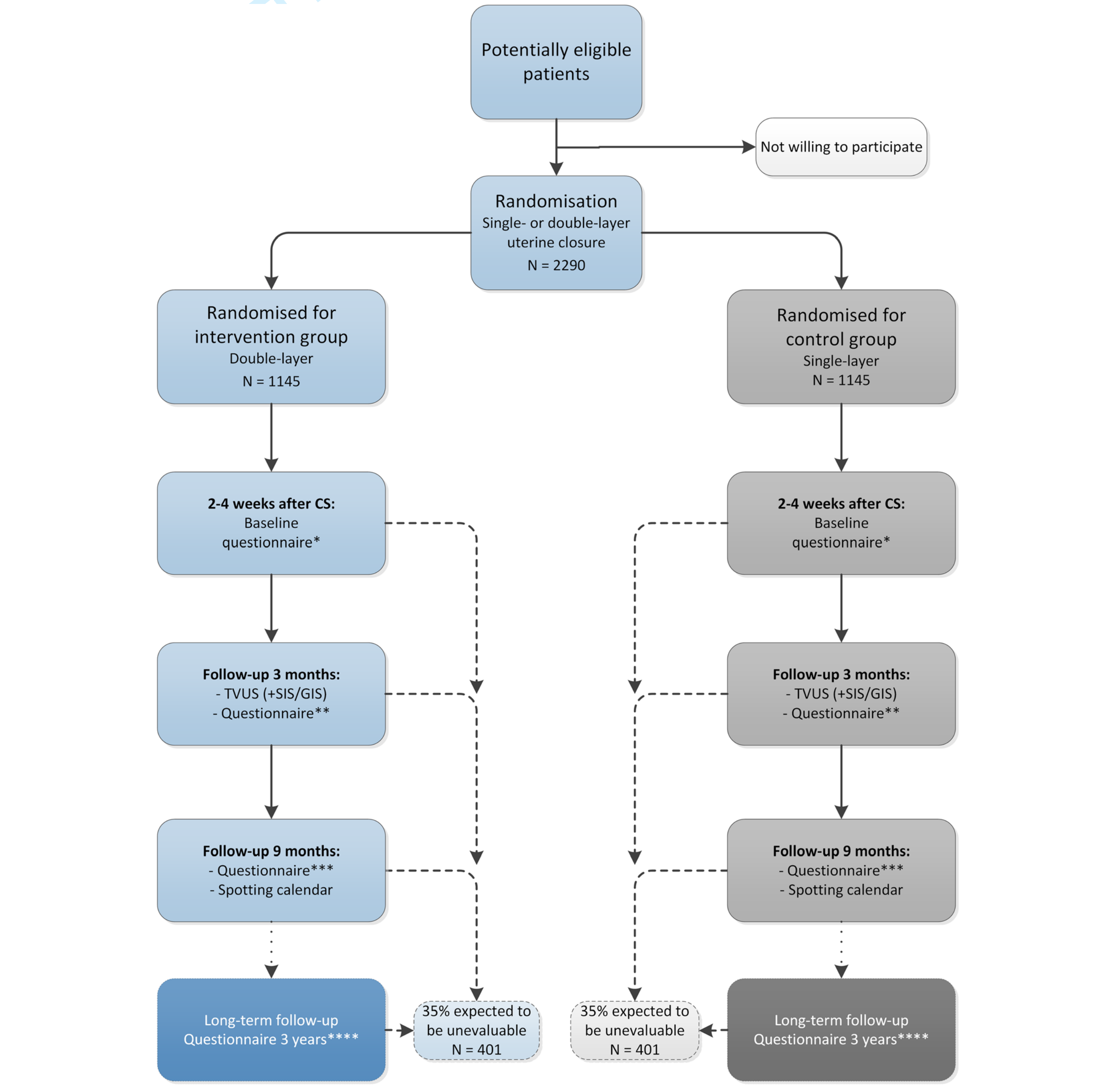

Fig. 1 Flowchart of the 2Close study. * = baseline questions, EQ-5D-5L. ** = symptom questionnaire, EQ-5D-5L, SF36, PROMIS SF8a, iMCQ, iPCQ. *** = symptom questionnaire, FSFI, EQ-5D-5L, SF36, iMCQ, iPCQ. **** = symptom questionnaire, fertility questionnaire, FSFI, EQ-5D-5L, SF36, PROMIS SF8a

thickness (excluding the endometrium) in the control group. The applied method, including endometrial saving technique or not, will be registered.

Niche evaluation

The care after CS will be according to the normal local protocol with the regular outpatient visit that is normally executed six weeks after the CS. This routine visit may be postponed to three months after the CS to enable an ultrasound evaluation to identify the existence of a niche, but participating centres may decide whether they want visits at six weeks (routine follow-up) and at three months (ultrasound follow-up) or only one visit after three months combining the regular control and the ultrasound follow-up. The ultrasound evaluation is standardised as proposed by Jordans et al. [11] (see Fig. 3). Based on this standardisation, we created an obligatory e-learning for all ultrasound performers to let all ultrasounds be performed in a uniform manner. To increase consistency and to improve the learning curve, we will evaluate a sample of ultrasounds in each centre based on recorded pictures and provide feedback to the examiners. Since it is known that a niche can be missed during TVUS only [8, 10, 25] we will additionally perform a saline infusion sonohysterography (SIS) or gel installation sonography (GIS) in case no niche is observed during the normal TVUS or if the ultrasound is inconclusive. It would be optimal to have a contrast enhanced ultrasound in all women when the uterine cavity or niche are not naturally filled with fluid, but we have chosen for this approach to prevent unnecessary burden for the participants and to reduce costs.

Outcome measures

Primary outcome measure

The primary outcome is the number of days of postmenstrual spotting during one cycle at nine months after CS. We defined postmenstrual spotting as brownish discharge for more than two days at the end of the menstruation, with a total duration (menstruation and spotting) of more than seven days, or intermenstrual blood loss that starts after the end of the menstruation. [8] The number of days of postmenstrual spotting will be counted as follows: days with brownish discharge (> two days) when the total duration of menstruation and spotting exceeds seven days + number of days with

For peer re

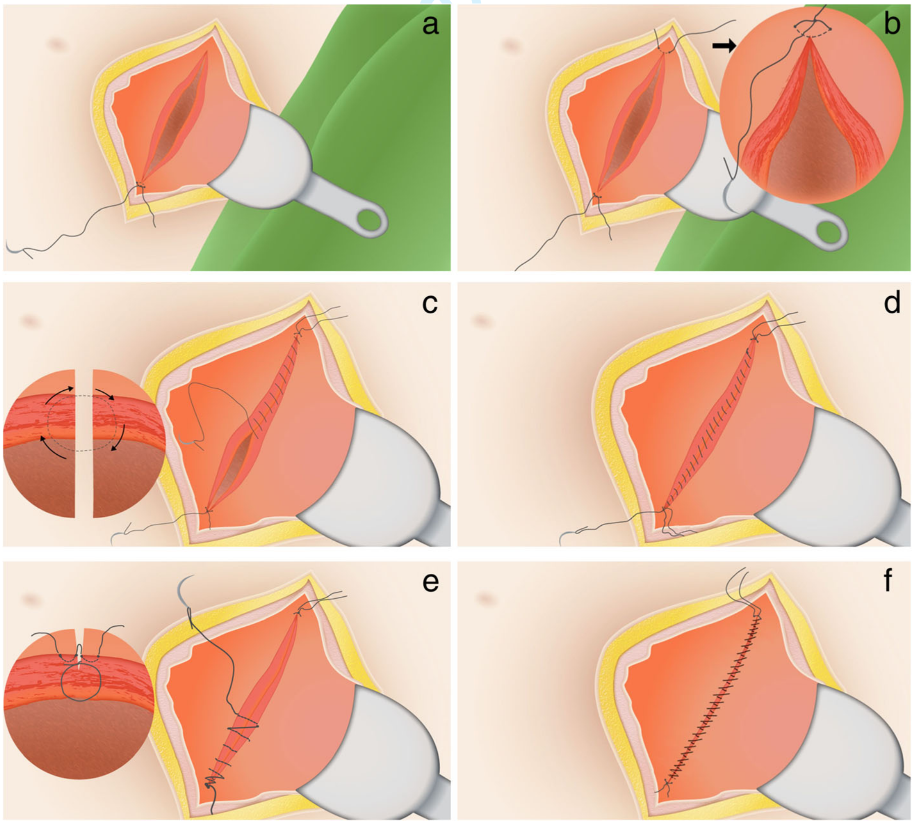

Fig. 2 Double-layer uterine closure technique. **a**. Step 1: lateral suture; **b**. Step 2: lateral suture on the other side; **c**. Step 3: First layer: full thickness, continuous, including large part of myometrium, including the endometrial layer; **d**. Step 4: End of this first layer; **e**. Step 5: Second layer: superficial continuous layer of serosal tissue, imbricating the first layer; **f**. Step 6: First and second layer should be closely connected

For peer review only - http://bmjopen.bmj.com/site/about/guidelines.xhtml

intermenstrual blood loss. Amenorrhoeic women, due to lactation, medication or other diseases, will not be evaluable for the primary outcome and will be left out of this analysis.

Secondary outcome measures at short-term

- Perioperative outcomes including blood loss, operative time, additional haemostatic sutures and complications.
- Menstruation characteristics, dysmenorrhea (visual analogue scale (VAS)), Quality of Life (QOL) using Short-Form-36 [26] and EQ-5D-5L [27, 28], societal reintegration (PROMIS Short-Form-8a [29]), sexual function using the Female Sexual Function Index (FSFI [30]), applied medical and/or surgical therapy because of gynaecological symptoms, all obtained through digital questionnaires, will be assessed at three and nine months follow-up.
- Ultrasound evaluation will be performed at three months follow-up using TVUS, in which RMT, adjacent myometrium thickness (AMT), presence of a niche (depth of ≥2mm), length, depth and width of the niche, presence of large niches (RMT < 50% of AMT, RMT <3mm) and niche volume will be measured.

Cost-effectiveness outcomes

Costs will be measured using adapted versions of the iMTA Productivity Cost Questionnaire (iPCQ [31]) and iMTA Medical Consumption Questionnaire (iMCQ [32]) from a health care and societal perspective at nine months of follow-up.

Secondary outcome measures at long-term

- Menstruation characteristics, pain, sexual functioning, QOL and social reintegration will be evaluated at three years follow-up.
- Reproductive outcomes at three years follow-up: % of women desiring to conceive, % of women that conceived including time to conceive, % of women with an ongoing pregnancy, the need for fertility treatment and pregnancy outcomes such as mode of delivery or complications will be determined.

Long-term outcomes will be presented in a separate article.

Data collection and data management

Intraoperative data

Immediately after the CS we will register relevant items regarding the delivery and CS in an eCRF, in which confidentiality and anonymity are ensured and audit trails are accessible. These items include: reason for planned or unplanned CS, emergency CS or not, whether women experienced contractions, dilatation, performed method for uterine closure, endometrial saving technique applied, used suturing material, extra haemostatic sutures, operative time, blood loss and complications.

Collection of baseline characteristics and patient reported outcomes

Baseline characteristics will be collected through a digital questionnaire at 2–4 weeks after caesarean section, sent to the e-mail address of participants. Since we will also include unplanned CSs, we decided that it is not possible for all participants to answer questions regarding baseline characteristics before the operation. Baseline parameters include maternal age, body mass index, social economic status, smoking habit, medical and obstetric history, gestational age and previous vaginal deliveries, all reported by the participant. We expect that the impact of niche related symptoms such as postmenstrual spotting on daily activities and sexual behaviour may be influenced by ethnic background and religion, therefore we will also register these characteristics. At three months, nine months and three years follow-up, again digital questionnaires will be sent to participants to assess the primary and secondary outcomes (see Fig. 1). At nine months, we ask participants record their exact menstrual and spotting pattern, if any, in an adjusted menstruation score chart. [33] Reminders for all questionnaires will be sent every two weeks, with a maximum of three times. When no response is given after the reminders, research nurses from participating centres will be asked to call the participant.

Data niche evaluation

Results of the TVUS and GIS or SIS, performed three months after CS, will be registered. Women will not receive information regarding the presence of a niche, since it has no clinical consequences so shortly after CS and this may influence the answers given in the questionnaires. Other important abnormalities visualised by ultrasound will be reported as usual.

Statistical issues

Sample size calculation

We use a superiority design since we expect double-layer closure to be favourable. Literature for making reliable estimations on postmenstrual spotting in relation to niches is scarce. We have used baseline data from the HysNiche [19] and LapNiche [15] study. We

For peer review only - http://bmjopen.bmj.com/site/about/guidelines.xhtml

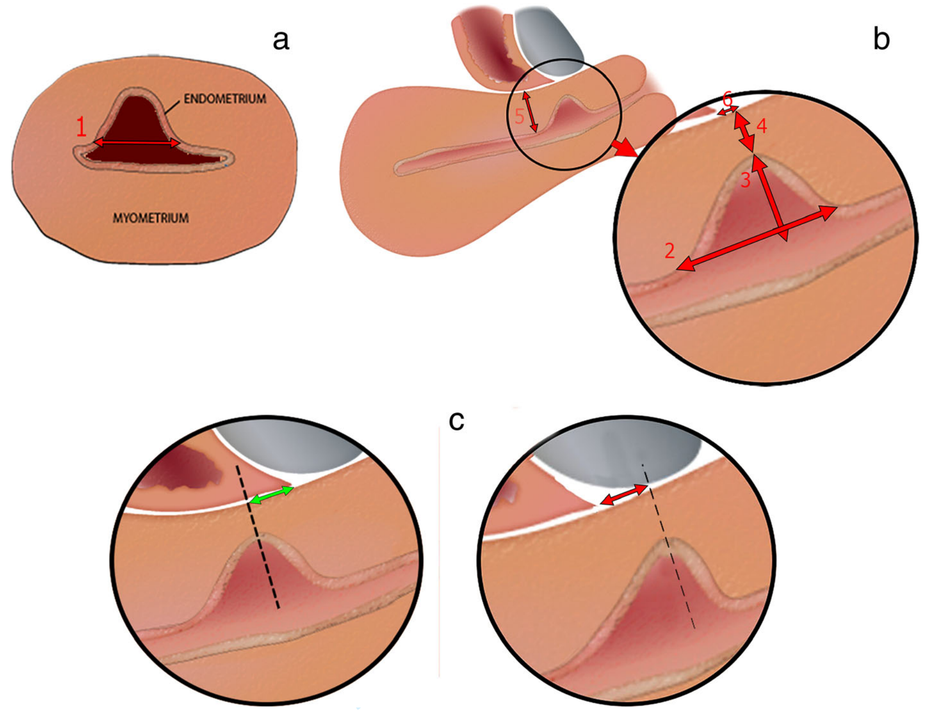

Fig. 3 Standardised transvaginal ultrasound evaluation of a niche in the non-pregnant uterus. **a**. Measured in the transversal plane: niche width (1); **b**. Measured in the sagittal plane: niche length (2), niche depth (from cervical canal until apex of the niche) (3), residual myometrium thickness (from deepest part of the niche until the serosa) (4), adjacent myometrium thickness (myometrium thickness close to base of the niche) (5), distance from apex of the niche to vesico-vaginal fold (see **c**) (6); **c**. Measuring the niche relatively to the vesico-vaginal fold in the sagittal plane: positive value (green arrow, in mm) or negative value (red arrow, in mm)

estimate the mean number of spotting days to be 3.5 days/month in the total group. We consider a 15% reduction in the mean number of spotting days clinically relevant, which is 0.5 day/month reduction. Assuming a standard deviation (SD) of 3.4 and a two-sided significance level of 5%, a total of 1488 women need to be included to achieve a power of 80%. Increasing the sample size to take into account 35% of women unevaluable (due to drop-out, non-response or amenorrhoea) for the primary outcome, 2290 women need to be included.

Data-analysis

Data-analysis will be performed according to the intention to treat principle and additional per protocol analyses will be performed. A test will be considered statistically significant when the two-sided test shows a p-value < 0.05. Baseline characteristics will be presented using percentages, means with SD and 95% CI or medians with interquartile ranges (IQR), where appropriate.

The primary outcome, number of days of postmenstrual spotting, will be presented for both groups as mean with SD or median with IQR, and presented in a Box-Whisker graph to show the distribution. Differences in primary outcome between the groups will be tested using the independent t-test in case of normal distribution (possible after transformation of the outcome) or Mann-Whitney U test. An adjusted analysis will be performed using linear regression analysis in which we adjust for factors on which randomisation was stratified and for baseline factors on which relevant differences are observed despite randomisation.

Dichotomous secondary outcomes will be presented as percentages and RR with corresponding 95% CI. *P*-values will be calculated using the chi-square test or, if the expected count for at least one cell is below 5, using the Fisher exact test. Normally distributed continuous variables will be presented as means with SD, and differences between the groups will be calculated with an independent t-test. Non-normally distributed continuous variables will be presented as medians with IQR and differences between the groups will be calculated with Mann-Whitney U test. The questionnaires will be analysed using the appropriate algorithms and usual presentation methods (FSFI, EQ-5D-5L, SF36, PROMIS SF8a, iMCQ, iPCQ).

Comparison of primary outcome between women receiving single- and double-layer closure will be done as secondary analyses within each of the following subgroups separately:

For peer review only - http://bmjopen.bmj.com/site/about/guidelines.xhtml

1. Planned (without labour) or unplanned (in labour) CS
2. Emergency CS or not
3. Preterm (< 37 weeks gestational age) or term (≥ 37 week gestational age) CS
4. Presence (> 3cm) or absence (≤ 3cm) of dilatation
5. Placenta praevia or not
6. Presence or absence of specific maternal morbidity (e.g. diabetes, pre-eclampsia, haemolysis/elevated liver enzymes/low platelet count (HELLP) syndrome, immunodeficient women)
7. Singleton versus twin pregnancy
8. Natural cycle or hormonally induced withdrawal bleeding

Within the single-layer group (control group) we will compare the primary outcome between women in whom endometrial saving technique (split thickness) was applied and women in whom an endometrial saving technique was not applied (full thickness).

Economic evaluation

The economic evaluation will be performed alongside the RCT from a societal perspective. Both a cost-effectiveness and cost-utility analysis will be performed with a time horizon of nine months to relate the difference in societal and healthcare costs between double-layer and single-layer unlocked uterine closure during a CS to the difference in clinical effects. Healthcare costs include costs of primary and secondary care, complementary care and home care. Costs in other sectors include presence and absence from paid and unpaid work. The friction cost approach will be used to estimate indirect costs. For the valuation of health care utilization standard prices published in the Dutch Costing guidelines will be used. [34] Medication use will be valued using prices of the Royal Dutch Society for Pharmacy.

Societal costs will be related to the following effect measures in the economic evaluation: days with postmenstrual spotting and quality-adjusted life-years (QALYs) based on the Dutch tariff for the EuroQol (EQ-5D-5L). [27, 28, 35]

We hypothesise that double-layer uterine closure will reduce postmenstrual spotting and related consultations for gynaecological or fertility related problems and applied therapies, and as a consequence that it will be cost-effective in comparison with single-layer uterine closure.

The analysis will be done according to the intention to treat principle. Missing costs and effect data will be imputed using multiple imputation. Incremental cost-effectiveness ratios (ICERs) will be calculated by dividing the difference in mean total costs between the treatment groups by the difference in mean effects. Bootstrapping with 5000 replications will be used to estimate 95% CI around cost differences and the uncertainty surrounding the ICERs. Uncertainty surrounding the ICERs will be graphically presented on cost-effectiveness planes. Cost-effectiveness acceptability curves showing the probability that double-layer uterine closure is cost-effective in comparison with single-layer uterine closure for a range of different ceiling ratios will also be estimated. Adjustment for confounders and effect modifiers will be done if necessary. [36]

Interim analysis and safety monitoring

Because of the type of intervention, the Medical Ethics Committee (MEC) determined that the risk for participation is negligible. Therefore, we do not have a Data Safety Monitoring Committee. No interim analysis is planned.

All serious adverse events (SAEs) will be reported to the MEC by line listing yearly. Life threatening SAEs or an event that leads to death will be reported to the MEC immediately. All SAEs will be followed until they have abated, until a stable situation has been reached or the patient was discharged. We do not expect to terminate the study prematurely given the low risk of adverse events.

Confidentiality and data security

All participating centres receive a login name and password to gain access to ALEA 2.2, the web-secured randomisation database. Randomisation is performed pseudo-anonymously with only the initials and year of birth of the participants. Linking personal data to the study number can only be performed in the local participating centres or by the trial coordinator (SS). Written informed consent forms are stored in every centre in a lockable room. All forms and data will be archived for 15 years in the participating centres.

Discussion

In the last years, studies examining complications of CSs are increasing, including the development of niches or thin residual myometrium at the site of the previous CS and related symptoms. Both RMT and the presence of a niche have been associated with postmenstrual spotting. [8, 10] Double-layer unlocked closure has been shown to result in a thicker residual myometrium and as a consequence can possibly lead to a decrease of niche development after a CS compared to single-layer closure. [5, 22, 24, 37] However, the long-term clinical outcomes in terms of postmenstrual spotting or subfertility have not been studied previously or have not been related to ultrasound findings. We hypothesise that niche related postmenstrual spotting and fertility problems will reduce together with decrease in niche prevalence, in which

For peer review only

identification of the best uterine closure technique regarding RMT and niche development will be of great significance.

Strengths and limitations

The design of this study is one of the strengths; this is the first large RCT that will evaluate the effectiveness of double-layer uterine closure compared to single-layer uterine closure after CS regarding niche related gynaecological symptoms and reproductive outcomes with a long-term follow-up. The study is adequately powered. Randomisation is performed by using a web based randomisation program. Furthermore, all participants and examiners are blinded which reduces the chance for bias regarding reported symptoms and ultrasound findings. An additional strength is the uniform manner in which we try to perform double-layer closure and ultrasound evaluation, instructed by mandatory online instruction film and e-learning, respectively. Moreover, the 2Close study will compare the cost-effectiveness of both techniques which has never been done before. As we expect that double-layer closure will reduce the incidence of niche development and as a consequence that it could possibly reduce the gynaecological symptoms including postmenstrual spotting after CS, we assume double-layer closure to be more cost-effective. Also, we expect that double-layer closure will improve the chances of conceiving after CS and lower costs in fertility treatment.

We also expect some limitations. Baseline characteristics will be collected through questionnaires that are filled in by women in the first month after CS, which might lead to recall bias regarding medical history, complications during pregnancy and labour, and other baseline measurements. We decided to lower the administrative load for participating hospitals by obtaining these characteristics through the participants. Furthermore, there is no validated questionnaire available yet for postmenstrual spotting; therefore, the questionnaires that are used in the 2Close study are not adjusted or validated for these symptoms. Moreover, the surgical techniques performed during the CS in this study are standardised in both study arms except for saving the endometrium in the control group. There is no conclusive evidence whether or not to save the endometrium in the suture according to its influence on niche development. Therefore, we chose to leave this decision with the surgeons. There may possibly be a difference in the incidence of niche development between the participants receiving single-layer split thickness or full thickness closure, also when compared to the incidence of niche development in the double-layer group. This will be further examined in a subgroup analysis.

To prevent bias regarding niche evaluation three months after CS, all ultrasonographic examiners are trained by an online learning program and a sample of ultrasounds will be evaluated. The learning module is based on the results of a Delphi procedure among international niche experts. [11] Although the niche examiners in the 2Close are trained by a standardised method, experience in measuring niches and as a consequence differences in niche measurement may occur among examiners.

Potential impact and implications

This study will gain insight in the most optimal uterine closure technique after CS which is relevant for women and gynaecologists, since we will focus on long-term gynaecological symptoms and reproductive outcomes in relation to changes of the lower uterine segment after CS and in particular niche development. Since many studies have already shown that RMT and niches are related to several symptoms and therapies for niche resection are being developed, we think it is necessary to provide evidence for the development of preventive strategies regarding niche related symptoms. It is important to realise that the best way to prevent a niche and its related symptoms, is to not perform a CS. But since it is often inevitable to perform a CS, care takers should perform it in the most optimal way.

After the results of this study become available, the most optimal and cost-effective technique can be implemented in order to reduce symptoms and problems in a subsequent pregnancy. This will not be difficult, since the technique is easy to learn and many gynaecologists and residents are familiar with it after the trial. Especially for a scheduled CS, women should be informed about the risk to develop a niche and the risk that it might cause symptoms or complications later in life.

Additional files

Additional file 1: Affiliations of all 32 participating hospitals in the Netherlands that granted approval. The board of the hospitals granted approval to participate and to start recruiting patients. (DOCX 15 kb)

Additional file 2: Text of the online standardised instruction film for double-layer closure of the uterotomy. The spoken text in the online instruction film, which shows a standardised way to perform double-layer closure of the uterotomy, has been translated into English. (DOCX 15 kb)

Abbreviations

AMT: Adjacent myometrium thickness; BROK: Basic course Regulations and Organisation for Clinical researchers; CI: Confidence interval; eCRF: electronic case report form; CS: Caesarean section; GCP: Good clinical practice; GIS: Gel installation sonohysterography; ICER: Incremental cost-effectiveness ratio; iMCQ: iMTA Medical Consumption Questionnaire; iMTA: Institute for Medical Technology Assessment; iPCQ: iMTA Productivity Cost Questionnaire; IQR: Interquartile range; MEC: Medical ethics committee; OR: Odds ratio; QALY: Quality adjusted life years; QOL: Quality of life; RCT: Randomised controlled trial; RMT: Residual myometrium thickness; RR: Relative risk; SAE: Serious adverse event; SD: Standard deviation; SIS: Saline infusion sonohysterography; TVUS: Transvaginal ultrasound; VAS: Visual analogue scale

For peer review only

Acknowledgements
None.

Funding
This study group received a grant from The Netherlands Organisation for Health Research and Development (ZonMw, project number: 843002605, grant number: 80–84300–98-62021), a Dutch organization that supports research in health and health innovation. The funder peer-reviewed the study design and will receive an annual progress report of the trial, but has no further role in the collection, analysis and interpretation of data, writing of the manuscript or decision to submit the report for publication.

Availability of data and materials
Data entry and secure storage is performed with data management system OpenClinica (OpenClinica LLC and collaborators, facilitated by TraIT (Translational Research IT infrastructure) and hosted by Vancis, which meets the ISO 27001 standard for information security management, version: 3.6). Data control during the trial is possible. The trial will be monitored on-site by a quality advisor of the Dutch Consortium for Studies in Women's Health and Reproduction. The quality advisor will also perform data verification at the end of the trial. The NVOG Consortium stores all data from clinical trials centrally so data will not become publicly available but will be available upon reasonable request to the corresponding author and with permission of the NVOG Consortium. Trial data will be accessible for verification or other research purposes, after an embargo period of 12 months in order to enable publication of the results by the project group.

Authors' contributions
JH, IJ, LvdV, MB, CdG, CL, RdL and WH made substantial contributions to the conception and design of the study. SS is responsible for the overall logistical aspects of the trial and general acquisition of data. SS and IJ drafted the manuscript. PV and JB provided statistical support regarding future analyses. EP, EB1, CR, MH, WvB, HV, JvL, HvV, RR, MS, CJ, WH, AF, KK, HS, JL, KdB, SC, DS, AO, MK, DP, LdV, EvB, MB, AH, WM, KD, EB2 and HvE made substantial contributions to acquisition of data. All authors critically revised the manuscript, approved the final version of the manuscript and agreed to be accountable for all aspects of the manuscript ensuring that questions related to the accuracy or integrity of the work are appropriately investigated and resolved.

Ethics approval and consent to participate
This study is approved by the ethics committee of Amsterdam UMC - location VUmc (reference number: 2015.462) and by the boards of all participating hospitals (Additional file 1). Protocol amendments will be communicated to participating hospitals, after approval of the central MEC of Amsterdam UMC – location VUmc. This trial is registered in the Dutch Trial Register (NTR5480, www.trialregister.nl). Counselling of eligible women will be done according to the 'good clinical practice' (GCP) guidelines or doctors certified according to Basic course Regulations and Organisation for Clinical researchers (BROK). After reading the patient information form and counselling by GCP- or BROK-certified personnel, women will be asked for written consent. This informed consent form will be obtained before participation in the clinical trial.
The research team declares that the SPIRIT guidelines were followed. All recommended items of the SPIRIT 2013 checklist were addressed in this clinical trial protocol.
The trial results will be communicated after the final inclusion and follow-up through publication in a peer-reviewed international journal and in an international congress. Participants will be informed through e-mail and the study website.

Consent for publication
Not applicable.

Competing interests
All authors declare that they have no competing interests.

Publisher's Note
Springer Nature remains neutral with regard to jurisdictional claims in published maps and institutional affiliations.

Author details
[1]Department of Obstetrics and Gynaecology, Research institutes 'Amsterdam Cardiovascular Sciences' and 'Amsterdam Reproduction and Development', Amsterdam UMC, Vrije Universiteit Amsterdam, De Boelelaan 1117, 1081 HV Amsterdam, Netherlands. [2]Department of Obstetrics and Gynaecology, Deventer Hospital, Nico Bolkesteinlaan 75, 7416 SE Deventer, the Netherlands. [3]Department of Obstetrics and Gynaecology, Máxima Medical Centre, De Run 4600, 5504 DB Veldhoven, the Netherlands. [4]Department of Epidemiology and Biostatistics, Vrije Universiteit Amsterdam, De Boelelaan 1105, 1081 HV Amsterdam, the Netherlands. [5]Department of Health sciences, Faculty of Science, Research institute 'Amsterdam Public Health', Vrije Universiteit Amsterdam, De Boelelaan 1085, 1081 HV Amsterdam, the Netherlands. [6]Department of Obstetrics and Gynaecology, Amsterdam UMC, University of Amsterdam, Meibergdreef 9, 1105 AZ Amsterdam, the Netherlands. [7]Department of Obstetrics and Gynaecology, OLVG-oost, Oosterpark 9, 1091 AC Amsterdam, the Netherlands. [8]Department of Obstetrics and Gynaecology, OLVG-west, Jan Tooropstraat 164, 1061 AE Amsterdam, the Netherlands. [9]Department of Obstetrics and Gynaecology, Westfriesgasthuis, Maelsonstraat 3, 1624 NP Hoorn, the Netherlands. [10]Department of Obstetrics and Gynaecology, Flevo hospital, Hospitaalweg 1, 1315 RA Almere, the Netherlands. [11]Department of Obstetrics and Gynaecology, Tergooi hospital, Rijksstraatweg 1, 1261 AN Blaricum, the Netherlands. [12]Department of Obstetrics and Gynaecology, Catharina hospital, Michelangelolaan 2, 5623 EJ Eindhoven, the Netherlands. [13]Department of Obstetrics and Gynaecology, Jeroen Bosch hospital, Henri Dunantstraat 1, 5223 GZ 's-Hertogenbosch, the Netherlands. [14]Department of Obstetrics and Gynaecology, Leiden University Medical Centre, Albinusdreef 2, 2333 ZA Leiden, the Netherlands. [15]Department of Obstetrics and Gynaecology, Groene Hart hospital, Bleulandweg 10, 2803 HH Gouda, the Netherlands. [16]Department of Obstetrics and Gynaecology, Haaglanden Medical Centre – Westeinde hospital, Lijnbaan 32, 2512 VA Den Haag, the Netherlands. [17]Department of Obstetrics and Gynaecology, Haga hospital, Els-Borst-Eilersplein 275, 2545 AA Den Haag, the Netherlands. [18]Department of Obstetrics and Gynaecology, Reinier de Graaf hospital, Reinier de Graafweg 5, 2625 AD Delft, the Netherlands. [19]Department of Obstetrics and Gynaecology, Research school 'GROW', Maastricht University Medical Centre, P. Debyelaan 25, 6229 HX Maastricht, the Netherlands. [20]Department of Obstetrics and Gynaecology, Zuyderland Medical Centre, Henri Dunantstraat 5, 6419 PC Heerlen, the Netherlands. [21]Department of Obstetrics and Gynaecology, Rijnstate hospital, Wagnerlaan 55, 6815 AD Arnhem, the Netherlands. [22]Department of Obstetrics and Gynaecology, Radboud University Nijmegen Medical Centre, Geert Grooteplein Zuid 10, 6525 GA Nijmegen, the Netherlands. [23]Department of Obstetrics and Gynaecology, Canisius-Wilhelmina hospital, Weg door Jonkerbos 100, 6532 SZ Nijmegen, the Netherlands. [24]Department of Obstetrics and Gynaecology, Bernhoven hospital, Nistelrodeseweg 10, 5406 PT Uden, the Netherlands. [25]Department of Obstetrics and Gynaecology, Röpcke-Zweers hospital, Jan Weitkamplaan 4a, 7772 SE Hardenberg, the Netherlands. [26]Department of Obstetrics and Gynaecology, Amphia hospital, Langendijk 75, 4819 EV Breda, the Netherlands. [27]Department of Obstetrics and Gynaecology, Sint Franciscus hospital, Kleiweg 500, 3045 PM Rotterdam, the Netherlands. [28]Department of Obstetrics and Gynaecology, Sint Antonius hospital, Koekoekslaan 1, 3435 CM Nieuwegein, the Netherlands. [29]Department of Obstetrics and Gynaecology, Birth Centre Wilhelmina Children hospital/ University Medical Centre Utrecht, Lundlaan 6, 3584 EA Utrecht, the Netherlands. [30]Department of Obstetrics and Gynaecology, Gelre hospital – location Apeldoorn, Albert Schweitzerlaan 31, 7334 DZ Apeldoorn, the Netherlands. [31]Department of Obstetrics and Gynaecology, Gelre hospital – location Zutphen, Den Elterweg 77, 7207 AE Zutphen, the Netherlands. [32]Department of Obstetrics and Gynaecology, Diakonessenhuis, Bosboomstraat 1, 3582 KE Utrecht, the Netherlands. [33]Department of Obstetrics and Gynaecology, Meander Medical Centre, Maatweg 3, 3813 TZ Amersfoort, the Netherlands. [34]Department of Obstetrics and Gynaecology, Isala clinics, Dokter van Heesweg 2, 8025 AB Zwolle, the Netherlands.

Received: 20 February 2018 Accepted: 12 February 2019
Published online: 04 March 2019

References
1. World Health Organization Human Reproduction Programme. WHO statement on caesarean section rates. Sex Reprod Health. 2015;02:1–8 (WHO/RHR/15.02).
2. Perined: Perinatale Zorg in Nederland 2016. In Utrecht: Perined; 2018.

For peer review only - http://bmjopen.bmj.com/site/about/guidelines.xhtml

3. Naji O, Wynants L, Smith A, Abdallah Y, Saso S, Stalder C, Van Huffel S, Ghaem-Maghami S, Van Calster B, Timmerman D, et al. Does the presence of a caesarean section scar affect implantation site and early pregnancy outcome in women attending an early pregnancy assessment unit? Hum Reprod. 2013;28(6):1489–96.
4. Naji O, Wynants L, Smith A, Abdallah Y, Stalder C, Sayasneh A, McIndoe A, Ghaem-Maghami S, Van Huffel S, Van Calster B, et al. Predicting successful vaginal birth after cesarean section using a model based on cesarean scar features examined by transvaginal sonography. Ultrasound Obstet Gynecol. 2013;41(6):672–8.
5. Roberge S, Demers S, Berghella V, Chaillet N, Moore L, Bujold E. Impact of single- vs double-layer closure on adverse outcomes and uterine scar defect: a systematic review and metaanalysis. Am J Obstet Gynecol. 2014; 211(5):453–60.
6. Hesselman S, Hogberg U, Ekholm-Selling K, Rassjo EB, Jonsson M. The risk of uterine rupture is not increased with single- compared with double-layer closure: a Swedish cohort study. BJOG. 2015;122(11):1535–41.
7. Clark EA, Silver RM. Long-term maternal morbidity associated with repeat cesarean delivery. Am J Obstet Gynecol. 2011;205(6 Suppl):S2–10.
8. Bij de Vaate AJ, Brolmann HA, van der Voet LF, van der Slikke JW, Veersema S, Huirne JA. Ultrasound evaluation of the cesarean scar: relation between a niche and postmenstrual spotting. Ultrasound Obstet Gynecol. 2011;37(1):93–9.
9. Bij de Vaate AJ, van der Voet LF, Naji O, Witmer M, Veersema S, Brolmann HA, Bourne T, Huirne JA. Prevalence, potential risk factors for development and symptoms related to the presence of uterine niches following cesarean section: systematic review. Ultrasound Obstet Gynecol. 2014;43(4):372–82.
10. van der Voet LF, Bij de Vaate AM, Veersema S, Brolmann HA, Huirne JA. Long-term complications of caesarean section. The niche in the scar: a prospective cohort study on niche prevalence and its relation to abnormal uterine bleeding. BJOG. 2014;121(2):236–44.
11. Jordans IPM, de Leeuw RA, Stegwee SI, Amso NN, Barri-Soldevila PN, van den Bosch T, Bourne T, Brolmann HAM, Donnez O, Dueholm M, et al. Sonographic examination of uterine niche in non-pregnant women: a modified Delphi procedure. Ultrasound Obstet Gynecol. 2019;53(1):107–15.
12. Gurol-Urganci I, Bou-Antoun S, Lim CP, Cromwell DA, Mahmood TA, Templeton A, van der Meulen JH. Impact of caesarean section on subsequent fertility: a systematic review and meta-analysis. Hum Reprod. 2013;28(7):1943–52.
13. Vervoort AJ, Uittenbogaard LB, Hehenkamp WJ, Brolmann HA, Mol BW, Huirne JA. Why do niches develop in caesarean uterine scars? Hypotheses on the aetiology of niche development. Hum Reprod. 2015;30(12):2695–702.
14. van der Voet LF, Vervoort AJ, Veersema S, Bij de Vaate AJ, Brolmann HA, Huirne JA. Minimally invasive therapy for gynaecological symptoms related to a niche in the caesarean scar: a systematic review. BJOG. 2014;121(2): 145–56.
15. Vervoort A, Vissers J, Hehenkamp WJK, Brolmann HAM, Huirne JAF. The effect of laparoscopic resection of large niches in the uterine caesarean scar on symptoms, ultrasound findings and quality of life: a prospective cohort study. BJOG. 2018;125(3):317–25.
16. Vervoort AJ, Van der Voet LF, Witmer M, Thurkow AL, Radder CM, van Kesteren PJ, Quartero HW, Kuchenbecker WK, Bongers MY, Geomini PM, et al. The HysNiche trial: hysteroscopic resection of uterine caesarean scar defect (niche) in patients with abnormal bleeding, a randomised controlled trial. BMC Womens Health. 2015;15:103.
17. Nikkels C, Vervoort A, Mol BW, Hehenkamp WJK, Huirne JAF, Brolmann HAM. IDEAL framework in surgical innovation applied on laparoscopic niche repair. Eur J Obstet Gynecol Reprod Biol. 2017;215:247–53.
18. Huirne JAF, Vervoort A, Leeuw R, Brolmann HAM, Hehenkamp WJK. Technical aspects of the laparoscopic niche resection, a step-by-step tutorial. Eur J Obstet Gynecol Reprod Biol. 2017;219:106–12.
19. Vervoort A, van der Voet LF, Hehenkamp W, Thurkow AL, van Kesteren P, Quartero H, Kuchenbecker W, Bongers M, Geomini P, de Vleeschouwer L, et al. Hysteroscopic resection of a uterine caesarean scar defect (niche) in women with postmenstrual spotting: a randomised controlled trial. BJOG. 2018;125(3): 326–34.
20. Abalos E, Addo V, Brocklehurst P, El Sheikh M, Farrell B, Gray S, Hardy P, Juszczak E, Mathews JE, Masood SN, et al. Caesarean section surgical techniques (CORONIS): a fractional, factorial, unmasked, randomised controlled trial. Lancet. 2013;382(9888):234–48.
21. The CAESAR study collaborative group. Caesarean section surgical techniques: a randomised factorial trial (CAESAR). BJOG. 2010;117(11):1366–76.
22. Di Spiezio Sardo A, Saccone G, McCurdy R, Bujold E, Bifulco G, Berghella V. Risk of cesarean scar defect in single- versus double-layer uterine closure: a systematic review and meta-analysis of randomized controlled trials. Ultrasound Obstet Gynecol. 2017;50:578–83.
23. Dodd JM, Anderson ER, Gates S, Grivell RM. Surgical techniques for uterine incision and uterine closure at the time of caesarean section. Cochrane Database Syst Rev. 2014;7:CD004732.
24. Stegwee SI, Jordans I, van der Voet LF, van de Ven PM, Ket J, Lambalk CB, de Groot C, Hehenkamp W, Huirne J. Uterine caesarean closure techniques affect ultrasound findings and maternal outcomes: a systematic review and meta-analysis. BJOG. 2018;125(9):1097–108.
25. Osser OV, Jokubkiene L, Valentin L. Cesarean section scar defects: agreement between transvaginal sonographic findings with and without saline contrast enhancement. Ultrasound Obstet Gynecol. 2010;35(1):75–83.
26. Aaronson NK, Muller M, Cohen PD, Essink-Bot ML, Fekkes M, Sanderman R, Sprangers MA, te Velde A, Verrips E. Translation, validation, and norming of the Dutch language version of the SF-36 health survey in community and chronic disease populations. J Clin Epidemiol. 1998;51(11):1055–68.
27. Dolan P. Modeling valuations for EuroQol health states. Med Care. 1997; 35(11):1095–108.
28. EuroQol--a new facility for the measurement of health-related quality of life. Health Policy. 1990;16(3):199–208.
29. Ability to Participate in Social Roles and Activities, Short Form 8a [http://www.healthmeasures.net/explore-measurement-systems/promis/intro-to-promis/list-of-adult-measures].
30. Rosen R, Brown C, Heiman J, Leiblum S, Meston C, Shabsigh R, Ferguson D, D'Agostino R Jr. The female sexual function index (FSFI): a multidimensional self-report instrument for the assessment of female sexual function. J Sex Marital Ther. 2000;26(2):191–208.
31. Handleiding iMTA Productivity Cost Questionnaire (iPCQ). [http://www.imta.nl].
32. Handleiding iMTA Medical Cost Questionnaire (iMCQ). [http://www.imta.nl].
33. Maarse M, Bij de Vaate AJM, Huirne JAF, Brölmann HAM. De maandkalender, een hulpmiddel voor een efficiënte menstruatieanamnese. Ned Tijdschr Obstet Gynaecol. 2011;124:231–5.
34. Hakkaart-van Roijen L, van der Linden N, Bouwmans CAM, Kanters TA, Tan SS. Kostenhandleiding: Methodologie van kostenonderzoek en referentieprijzen voor economische evaluaties in de gezondheidszorg. Institute for Medical Technology Assessment; Erasmus Universiteit Rotterdam. Zorginstituut Nederland; 2016.
35. Lamers LM, Stalmeier PF, McDonnell J, Krabbe PF, van Busschbach JJ. Measuring the quality of life in economic evaluations: the Dutch EQ-5D tariff. Ned Tijdschr Geneeskd. 2005;149(28):1574–8.
36. Fenwick E, O'Brien BJ, Briggs A. Cost-effectiveness acceptability curves--facts, fallacies and frequently asked questions. Health Econ. 2004;13(5):405–15.
37. Glavind J, Madsen LD, Uldbjerg N, Dueholm M. Ultrasound evaluation of cesarean scar after single- and double-layer uterotomy closure: a cohort study. Ultrasound Obstet Gynecol. 2013;42(2):207–12.

Ready to submit your research? Choose BMC and benefit from:

- fast, convenient online submission
- thorough peer review by experienced researchers in your field
- rapid publication on acceptance
- support for research data, including large and complex data types
- gold Open Access which fosters wider collaboration and increased citations
- maximum visibility for your research: over 100M website views per year

At BMC, research is always in progress.

Learn more biomedcentral.com/submissions

For peer review only

Supplementary appendix

Appendix S1. List of collaborators.

**Hospital**	**Site principal investigator**
Amphia hospital, Breda	Dimitri NM Papatsonis
Amsterdam UMC, Univ of Amsterdam, Amsterdam	Eva Pajkrt
Amsterdam UMC, VU University, Amsterdam	Wouter JK Hehenkamp
Bernhoven hospital, Uden	Angèle LM Oei
Birth Centre Wilhelmina Children Hospital/University Medical Centre Utrecht, Utrecht	Mireille N Bekker
Canisius-Wilhelmina hospital, Nijmegen	Daniela H Schippers
Catharina hospital, Eindhoven	Huib AAM van Vliet
Deventer ziekenhuis, Deventer	Lucet van der Voet
Diakonessenhuis, Utrecht	Nico WE Schuitemaker
Dijklander hospital – location Hoorn	Majoie Hemelaar
Flevo hospital, Almere	WM (Marchien) van Baal
Gelre hospital – location Apeldoorn	Anjoke JM Huisjes
Gelre hospital – location Zutphen	Wouter J Meijer
Groene Hart hospital, Gouda	CAH (Ineke) Janssen
Haaglanden Medical Centre – Westeinde hospital, Den Haag	Wietske Hermes
Haga hospital, Den Haag	AH (Hanneke) Feitsma
Isala clinics, Zwolle	Hugo WF van Eijndhoven
Jeroen Bosch hospital, 's-Hertogenbosch	Robbert JP Rijnders
Leiden University Medical Centre, Leiden	Marieke Sueters
Maastricht University Medical Centre, Research school 'GROW', Maastricht	HCJ (Liesbeth) Scheepers
Máxima Medical Centre, Veldhoven	Judith OEH van Laar
Meander Medical Centre, Amersfoort	Elisabeth MA Boormans
OLVG-oost, Amsterdam	Paul JM van Kesteren
OLVG-west, Amsterdam	Celine M Radder
Radboud University Nijmegen Medical Centre, Nijmegen	Esther Hink
Reinier de Graaf hospital, Delft	Kitty Kapiteijn
Rijnstate hospital, Arnhem	Karin de Boer
Röpcke-Zweers hospital, Hardenberg	Mesrure Kaplan
Sint Antonius Hospital, Nieuwegein	Erik van Beek
Sint Franciscus Hospital, Rotterdam	LHM (Marloes) de Vleeschouwer
Tergooi hospital, Blaricum	Harry Visser
Zuyderland Medical Centre, Heerlen	Josje Langenveld

Collaborators (alfabetic order – surname of collaborator):
WM (Marchien) van Baal, Erik van Beek, Mireille N Bekker, Karin de Boer, Elisabeth MA Boormans, Judith E Bosmans, Hugo WF van Eijndhoven, Mohamed El Alili, AH (Hanneke) Feitsma, Majoie Hemelaar, Wietske Hermes, Esther Hink, Anjoke JM Huisjes, CAH (Ineke) Janssen, Kitty Kapiteijn, Mesrure Kaplan, Paul JM van Kesteren, Judith OEH van Laar, Josje Langenveld, Wouter J Meijer, Angèle LM Oei, Eva Pajkrt, Dimitri NM Papatsonis, Celine M Radder, Robbert JP Rijnders, HCJ (Liesbeth) Scheepers, Daniela H Schippers, Nico WE Schuitemaker, Marieke Sueters, Harry Visser, Huib AAM van Vliet, LHM (Marloes) de Vleeschouwer

For peer review only

Supplementary tables

Table S1: Costs of the intervention per participant

Resources	Units	Unit price, €	Costs, €
Additional suture material	One piece	6.14	6.14
Additional operation time, academic hospital (3.9 minutes)	Hour	1379.27	89.65
Total costs per participant in academic hospital			95.79
Additional operation time, non-academic hospital (3.9 minutes)	Hour	1000	65.00
Total costs per participant in non-academic hospital			71.14

Table S2: Description of follow-up complete cases and missing cases by effect and cost outcomes without excluding cases of amenorrhoea

	Complete cases			Missing cases (%)		
	Intervention	*Control*	*Total*	*Intervention*	*Control*	*Total*
Spotting days	936	940	1876	212 (23)	204 (22)	416 (22)
QALY	845	851	1696	303 (36)	293 (34)	596 (35)
Primary care costs	913	924	1837	235 (26)	220 (24)	455 (25)
Secondary care costs	838	833	1671	310 (37)	311 (37)	621 (37)
Medication costs	834	825	1659	314 (38)	319 (39)	633 (38)
Total healthcare costs	830	823	1653	318 (38)	321 (39)	639 (39)
Informal care costs	913	924	1837	235 (26)	220 (24)	455 (25)
Absenteeism costs at work	852	849	1701	296 (35)	295 (35)	591 (35)
Absenteeism costs at unpaid work	905	921	1826	243 (27)	223 (24)	466 (25)
Presenteeism costs	680	690	1370	468 (69)	454 (66)	922 (67)
Total lost productivity costs	677	689	1366	471 (69)	455 (66)	926 (68)
Total societal costs	664	671	1335	484 (73)	473 (70)	957 (72)

Table S3: Probability of the intervention being cost-effective at different willingness-to-pay thresholds for spotting days

	Main analyses – spotting days	
WTP €/ unit of effect gained	*Societal Perspective*	*Healthcare perspective*
0	0.23	0.53
31.6 (1 working hour)	0.23	0.53
126.4 (4 working hours)	0.24	0.54
252.8 (8 working hours)	0.24	0.56
	**Main analyses - QALYs**	
0	0.23	0.53
10000	0.21	0.40
20000	0.19	0.32
30000	0.18	0.27
40000	0.17	0.25
50000	0.16	0.23

WTP=willingness-to-pay; QALY=quality-adjusted life-years

For peer review only

CHEERS Checklist
Items to include when reporting economic evaluations of health interventions

The **ISPOR CHEERS Task Force Report**, *Consolidated Health Economic Evaluation Reporting Standards (CHEERS)—Explanation and Elaboration: A Report of the ISPOR Health Economic Evaluations Publication Guidelines Good Reporting Practices Task Force,* provides examples and further discussion of the 24-item CHEERS Checklist and the CHEERS Statement. It may be accessed via the *Value in Health* or via the ISPOR Health Economic Evaluation Publication Guidelines – CHEERS: Good Reporting Practices webpage: http://www.ispor.org/TaskForces/EconomicPubGuidelines.asp

Section/item	Item No	Recommendation	Reported on page No/ line No
**Title and abstract**			
Title	1	Identify the study as an economic evaluation or use more specific terms such as "cost-effectiveness analysis", and describe the interventions compared.	1
Abstract	2	Provide a structured summary of objectives, perspective, setting, methods (including study design and inputs), results (including base case and uncertainty analyses), and conclusions.	2
**Introduction**			
Background and objectives	3	Provide an explicit statement of the broader context for the study. Present the study question and its relevance for health policy or practice decisions.	4
**Methods**			
Target population and subgroups	4	Describe characteristics of the base case population and subgroups analysed, including why they were chosen.	6
Setting and location	5	State relevant aspects of the system(s) in which the decision(s) need(s) to be made.	7, 8
Study perspective	6	Describe the perspective of the study and relate this to the costs being evaluated.	7
Comparators	7	Describe the interventions or strategies being compared and state why they were chosen.	8
Time horizon	8	State the time horizon(s) over which costs and consequences are being evaluated and say why appropriate.	7
Discount rate	9	Report the choice of discount rate(s) used for costs and outcomes and say why appropriate.	7
Choice of health outcomes	10	Describe what outcomes were used as the measure(s) of benefit in the evaluation and their relevance for the type of analysis performed.	8 - 10
Measurement of effectiveness	11a	*Single study-based estimates:* Describe fully the design features of the single effectiveness study and why the single study was a sufficient source of clinical effectiveness data.	8, 9

	11b	*Synthesis-based estimates:* Describe fully the methods used for identification of included studies and synthesis of clinical effectiveness data.	NA
Measurement and valuation of preference based outcomes	12	If applicable, describe the population and methods used to elicit preferences for outcomes.	8 - 10
Estimating resources and costs	13a	*Single study-based economic evaluation:* Describe approaches used to estimate resource use associated with the alternative interventions. Describe primary or secondary research methods for valuing each resource item in terms of its unit cost. Describe any adjustments made to approximate to opportunity costs.	9, 10
	13b	*Model-based economic evaluation:* Describe approaches and data sources used to estimate resource use associated with model health states. Describe primary or secondary research methods for valuing each resource item in terms of its unit cost. Describe any adjustments made to approximate to opportunity costs.	NA
Currency, price date, and conversion	14	Report the dates of the estimated resource quantities and unit costs. Describe methods for adjusting estimated unit costs to the year of reported costs if necessary. Describe methods for converting costs into a common currency base and the exchange rate.	9, 10 table S1
Choice of model	15	Describe and give reasons for the specific type of decision-analytical model used. Providing a figure to show model structure is strongly recommended.	NA
Assumptions	16	Describe all structural or other assumptions underpinning the decision-analytical model.	NA
Analytical methods	17	Describe all analytical methods supporting the evaluation. This could include methods for dealing with skewed, missing, or censored data; extrapolation methods; methods for pooling data; approaches to validate or make adjustments (such as half cycle corrections) to a model; and methods for handling population heterogeneity and uncertainty.	10 - 12
**Results**			
Study parameters	18	Report the values, ranges, references, and, if used, probability distributions for all parameters. Report reasons or sources for distributions used to represent uncertainty where appropriate. Providing a table to show the input values is strongly recommended.	NA
Incremental costs and outcomes	19	For each intervention, report mean values for the main categories of estimated costs and outcomes of interest, as well as mean differences between the comparator groups. If applicable, report incremental cost-effectiveness ratios.	13 - 15 Table 2+3
Characterising uncertainty	20a	*Single study-based economic evaluation:* Describe the effects of sampling uncertainty for the estimated incremental cost and incremental effectiveness parameters, together with the impact	11

		of methodological assumptions (such as discount rate, study perspective).	
	20b	*Model-based economic evaluation:* Describe the effects on the results of uncertainty for all input parameters, and uncertainty related to the structure of the model and assumptions.	NA
Characterising heterogeneity	21	If applicable, report differences in costs, outcomes, or cost-effectiveness that can be explained by variations between subgroups of patients with different baseline characteristics or other observed variability in effects that are not reducible by more information.	12 ; 15
**Discussion**			
Study findings, limitations, generalisability, and current knowledge	22	Summarise key study findings and describe how they support the conclusions reached. Discuss limitations and the generalisability of the findings and how the findings fit with current knowledge.	16-18
**Other**			
Source of funding	23	Describe how the study was funded and the role of the funder in the identification, design, conduct, and reporting of the analysis. Describe other non-monetary sources of support.	19
Conflicts of interest	24	Describe any potential for conflict of interest of study contributors in accordance with journal policy. In the absence of a journal policy, we recommend authors comply with International Committee of Medical Journal Editors recommendations.	19, 20

For consistency, the CHEERS Statement checklist format is based on the format of the CONSORT statement checklist

The **ISPOR CHEERS Task Force Report** provides examples and further discussion of the 24-item CHEERS Checklist and the CHEERS Statement. It may be accessed via the *Value in Health* link or via the ISPOR Health Economic Evaluation Publication Guidelines – CHEERS: Good Reporting Practices webpage: http://www.ispor.org/TaskForces/EconomicPubGuidelines.asp

The citation for the CHEERS Task Force Report is:
Husereau D, Drummond M, Petrou S, et al. Consolidated health economic evaluation reporting standards (CHEERS)—Explanation and elaboration: A report of the ISPOR health economic evaluations publication guidelines good reporting practices task force. Value Health 2013;16:231-50.

BMJ Open

Cost-effectiveness of single- versus double-layer uterine closure during caesarean section on postmenstrual spotting: economic evaluation alongside a randomised controlled trial

Journal:	*BMJ Open*
Manuscript ID	bmjopen-2020-044340.R1
Article Type:	Original research
Date Submitted by the Author:	23-Mar-2021
Complete List of Authors:	Stegwee, Sanne; Amsterdam UMC Locatie VUmc, Obstetrics and Gynaecology<br>Ben, Ângela; Vrije Universiteit Amsterdam, Health Sciences<br>El Alili, Mohamed ; Vrije Universiteit Amsterdam<br>van der Voet, Lucet; Deventer Ziekenhuis, Obstetrics and Gynaecology<br>de Groot, Christianne; Amsterdam UMC Locatie VUmc, Obstetrics and Gynaecology<br>Bosmans, J; Vrije Universiteit Amsterdam, Health Sciences<br>Huirne, Judith; Amsterdam UMC Locatie AMC, Obstetrics and Gynaecology
**Primary Subject Heading**:	Obstetrics and gynaecology
Secondary Subject Heading:	Health economics
Keywords:	OBSTETRICS, Maternal medicine < OBSTETRICS, Ultrasonography < OBSTETRICS, HEALTH ECONOMICS

SCHOLARONE™
Manuscripts

I, the Submitting Author has the right to grant and does grant on behalf of all authors of the Work (as defined in the below author licence), an exclusive licence and/or a non-exclusive licence for contributions from authors who are: i) UK Crown employees; ii) where BMJ has agreed a CC-BY licence shall apply, and/or iii) in accordance with the terms applicable for US Federal Government officers or employees acting as part of their official duties; on a worldwide, perpetual, irrevocable, royalty-free basis to BMJ Publishing Group Ltd ("BMJ") its licensees and where the relevant Journal is co-owned by BMJ to the co-owners of the Journal, to publish the Work in this journal and any other BMJ products and to exploit all rights, as set out in our licence.

The Submitting Author accepts and understands that any supply made under these terms is made by BMJ to the Submitting Author unless you are acting as an employee on behalf of your employer or a postgraduate student of an affiliated institution which is paying any applicable article publishing charge ("APC") for Open Access articles. Where the Submitting Author wishes to make the Work available on an Open Access basis (and intends to pay the relevant APC), the terms of reuse of such Open Access shall be governed by a Creative Commons licence – details of these licences and which Creative Commons licence will apply to this Work are set out in our licence referred to above.

Other than as permitted in any relevant BMJ Author's Self Archiving Policies, I confirm this Work has not been accepted for publication elsewhere, is not being considered for publication elsewhere and does not duplicate material already published. I confirm all authors consent to publication of this Work and authorise the granting of this licence.

For peer review only

Cost-effectiveness of single- versus double-layer uterine closure during caesarean section on postmenstrual spotting: economic evaluation alongside a randomised controlled trial

Sanne I Stegwee[1]*, Ângela Jornada Ben[2], Mohamed El Alili[2], Lucet F van der Voet[3], Christianne JM de Groot[1], Judith E Bosmans[2], Judith AF Huirne[1]*, for the 2Close study group†

[1] Department of Obstetrics and Gynaecology, Amsterdam UMC, Vrije Universiteit, Amsterdam Reproduction & Development, Amsterdam, Netherlands

[2] Department of Health Sciences, Amsterdam Public Health, Faculty of Science, VU University, Amsterdam, The Netherlands

[3] Department of Obstetrics and Gynaecology, Deventer Hospital, Deventer, Netherlands

*Corresponding author during submission: SI Stegwee, MD, Amsterdam UMC - VU University, Department of Obstetrics and Gynaecology, De Boelelaan 1117, 1081 HV Amsterdam, The Netherlands. T: +31 20 4444807, F: +31 20 444 83 87, s.stegwee@amsterdamumc.nl.

*Corresponding author after publication: Prof JAF Huirne, Amsterdam UMC - VU University, Department of Obstetrics and Gynaecology, De Boelelaan 1117, 1081 HV Amsterdam, The Netherlands. T: +31 20 4444444, F: +31 20 444 83 87, j.huirne@amsterdamumc.nl.

†A full list of collaborators is provided in the appendix

For peer review only - http://bmjopen.bmj.com/site/about/guidelines.xhtml

Abstract

Objective: To evaluate the cost-effectiveness of double-layer compared to single-layer uterine closure after a first caesarean section (CS) from a societal and healthcare perspective.

Design: Economic evaluation alongside a multicentre, double-blind, randomised controlled trial

Setting: 32 hospitals in the Netherlands, 2016-18.

Participants: 2292 women ≥ 18 years undergoing a first CS were randomly assigned (1:1). Exclusion criteria were: inability for counselling, previous uterine surgery, known menstrual disorder, placenta increta or percreta, pregnant with three or more fetuses. 1144 women were assigned to single-layer and 1148 to double-layer closure. We included 1620 women with a menstrual cycle in the main analysis.

Interventions: Single-layer unlocked uterine closure and double-layer unlocked uterine closure with the second layer imbricating the first.

Main outcome measures: Spotting days, quality-adjusted life-years (QALYs), and societal costs at nine months of follow-up. Missing data were imputed using multiple imputation.

Results: No significant differences were found between single- versus double-layer closure in mean spotting days (1.44 and 1.40 days; mean difference [md] -0.045, 95% CI, -0.274; 0.364), QALYs (0.663 and 0.659; md -0.004, 95% CI, -0.013; 0.005), total healthcare costs (€760 and €728; md €-32, 95% CI, -390; 150), and total societal costs (€5426 and €5719; md €292, 95% CI, -518; 1096). The probability of the intervention being cost-effective at willingness-to-pay of 0, 10,000 and 20,000 €/QALY gained was 0.25, 0.23 and 0.22, respectively, (societal perspective), and 0.57, 0.48 and 0.41, respectively, (healthcare perspective).

Conclusion: Double-layer uterine closure is not cost-effective compared to single-layer uterine closure from both perspectives. If this is confirmed by our long-term reproductive follow-up, we suggest to adjust uterine closure technique guidelines.

Trial registration: Netherlands Trial Register (NTR5480/NL5380; trial finished).

Key words: caesarean section; uterine closure technique; postmenstrual spotting; cost-effectiveness analysis

For peer review only - http://bmjopen.bmj.com/site/about/guidelines.xhtml

Article summary

Strengths and limitations of this study

This study has been performed alongside large multicentre RCT, which is considered the best vehicle for economic evaluations

We prospectively collected data regarding costs and effect, and we used patient level information

All relevant costs for decision making were included in the analysis, to conduct an analysis from a societal perspective

Possible recall bias due to retrospective self-reported questionnaire over 3- and 6-month period

Generalizability of the results to other populations may be limited, as other healthcare practices and payment systems may exist

For peer review only

For peer review only - http://bmjopen.bmj.com/site/about/guidelines.xhtml

Introduction

Caesarean section (CS) rates rise globally and is the mode of delivery for approximately one in five live births globally.[1 2] As a consequence, a rise in morbidity related to CS is observed as well.[3] Severe morbidity associated with a subsequent pregnancy includes caesarean scar pregnancy, placenta accreta spectrum disorders and uterine rupture. However, less severe but more prevalent gynaecological morbidity related to a CS have recently gained more interest as well. Chronic maternal morbidity after CS includes dysmenorrhoea and abnormal uterine bleeding, which are both associated with a sonographically visible indentation at the site of the previous uterine incision.[4-6] This indentation is called a niche and is seen in approximately 60% of women after CS.[7 8] Of them, 30% develops abnormal uterine bleeding and more specifically, postmenstrual spotting.[5 6] This is brownish discharge at the end of the menstruation or blood loss in between two menstruations that is limiting women in daily life.[5] Over the last years, an increase in the development of medical treatments and surgical procedures to treat or remove the niche is observed, primarily aiming to reduce spotting.[9]

CS is the most common major surgical intervention.[10] However, there is no international guideline on the most optimal way to close the uterine incision while the specific closure technique may influence healing of the uterine wound. A specific issue on which no consensus exists is whether to use single- or double-layer closure of the uterine layers.[11 12] When comparing these techniques, no differences were found at short-term except for longer operation time after double-layer closure.[13 14] Nevertheless, previous studies also suggested that double-layer closure may result in better uterine scar healing and lower prevalence of large niches thereby possibly leading to lower medical costs than single-layer closure.[13 15] There is, however, a lack of studies on uterine closure techniques and their impact on maternal health outcomes related to gynaecological symptoms.[14] The impact of different uterine closure techniques on healthcare utilization, informal care, and lost productivity costs has never been investigated previously. As decision makers increasingly demand evidence of cost-effectiveness of health care interventions, conducting economic analysis alongside clinical trials is desirable because it allows the prospective collection of cost and effect data and the use of patient

For peer review only - http://bmjopen.bmj.com/site/about/guidelines.xhtml

level information for drawing inferences about additional costs and benefits of interventions.[16] In addition, regulatory and reimbursement agencies of many countries consider evidence of economic value along with clinical effectiveness.[16]

Therefore, the aim of this study was to perform a cost-effectiveness analysis of double-layer compared to single-layer uterine closure after a first CS from both a societal and healthcare perspective. We hypothesised that double-layer closure would reduce postmenstrual spotting and total societal and healthcare costs compared to single-layer closure as a result of less morbidity, despite slightly higher intervention costs of double-layer closure.

For peer review only

For peer review only - http://bmjopen.bmj.com/site/about/guidelines.xhtml

Methods

Study design

An economic evaluation was performed alongside a multicentre randomised controlled superiority trial comparing double-layer closure and single-layer uterine closure after a first CS. The study protocol and the effect paper have been published elsewhere[17 18] The study was approved by the Institutional Review Board (IRB) of Amsterdam UMC – location VU University medical centre (registration number 2015.462) and by the boards of all participating hospitals. No substantial changes were made to the protocol after commencement of the trial.[17] This trial-based economic evaluation is reported according to the Consolidated Health Economic Evaluating Reporting Standards (CHEERS) statement.[19]

Target population

All women who underwent a first CS, planned or unplanned, at one of the participating hospitals were asked to participate in the study. Inclusion criteria were sufficient command of the Dutch or English language, age 18 years or older and written informed consent. Exclusion criteria were: inadequate possibility for counselling (e.g., indication for emergency CS without being informed about the study previously, women in severe pain without adequate therapy), previous major uterine surgery (e.g., laparoscopic or laparotomic fibroid resection, septum resection), women with known causes of menstrual disorders (e.g., cervical dysplasia, communicating hydrosalpinx, uterine anomaly or endocrine disorders disturbing ovulation), placenta increta or percreta during the current pregnancy, or three or more foetuses during the current pregnancy. After informed consent had been signed and a CS was indicated, participants were randomly allocated to receive single-layer (control) or double-layer (intervention) closure of the uterine incision in a 1:1 ratio. Due to the nature of the treatment, surgeons performing the CS were not masked to the allocated method. Participants and sonographers were blinded to the allocation, researchers and statisticians were not. Detailed information about study design and randomization can be found in the study protocol.[17]

Choice of health outcomes

For peer review only - http://bmjopen.bmj.com/site/about/guidelines.xhtml

119 For this trial-based economic evaluation, two main health outcomes were used: postmenstrual spotting (referred to as spotting days in this paper) and Quality-Adjusted Life-Years (QALYs) at nine months after CS. Spotting days was chosen because it has been strongly related to a niche[5 6] (i.e., an indentation at the site of the caesarean scar with a depth of at least 2 mm[20]), which may be influenced by uterine closure technique.[8] QALY is routinely used as a summary outcome measure of health in economic evaluations, because it incorporates the impact of interventions on both the quantity and quality of life,[21] and allows decision makers to compare the effectiveness and cost-effectiveness of a range of interventions for different health conditions.[22]

127 **Study perspective and time-horizon**

128 This trial-based economic evaluation was performed from a societal and a healthcare perspective over a time-horizon of nine months. Therefore, discounting of costs and effects was not necessary. When a healthcare perspective is adopted, only the intervention costs and costs related to healthcare utilization are included in the analysis.[23] For the societal perspective, costs related to informal care and productivity losses are included in addition to intervention and healthcare utilization costs.[23]

133 **Setting and location**

134 In total, 32 hospitals in the Netherlands, both academic (n=6) and non-academic (n=26), collaborating within the Dutch Consortium for Healthcare Evaluation and Research in Obstetrics and Gynaecology (NVOG Consortium 2.0, www.zorgevaluatienederland.nl), participated in this study.[17] In the Netherlands, a CS is only performed in a hospital setting. In most cases, without maternal or neonatal complications, women will be discharged from the hospital after a CS after two or three days. All costs regarding the CS and admission days are standard care and are paid by an individual's health insurance. Maternity leave of at least ten weeks is regulated through the Employee Insurance Agency. Paternity leave is limited to one week. The first eight days after delivery, a maternity nurse visits the family at least three hours a day.

143 **Control and intervention condition**

For peer review only - http://bmjopen.bmj.com/site/about/guidelines.xhtml

The control group underwent single-layer closure of the uterus using unlocked continuous running multifilament sutures, which is the usual care provided by hospitals in the Netherlands.[17] In the intervention group, double-layer closure of the uterine incision was performed using unlocked multifilament continuous running sutures for both layers and the endometrial layer was included in the first layer. The second layer was a continuous running suture that imbricated the first. A mandatory online instruction video was shown to all surgeons in participating hospitals prior to participation for the intervention group. The exact procedures in both study arms regarding uterine closure are described in the study protocol.[17]

At baseline, data was collected on sociodemographic characteristics for all participants.[17]

Outcomes

Health outcomes

Spotting days was the primary outcome of the trial, and was defined as number of days with brownish discharge for more than two days at the end of the menstruation, with a total duration (menstruation and spotting) of more than seven days, or intermenstrual blood loss that started after the end of the menstruation.[5] Spotting days were self-reported by participants through a digital questionnaire at nine months after CS, including a calendar on which women could record daily blood loss during one month. Women who reported that they had no blood loss were classified as amenorrhoeic.

Health-related quality of life was measured using the EQ-5D-5L at baseline, and at three and nine months after CS.[24] The EQ-5D-5L has five dimensions of quality of life (mobility, self-care, usual activities, pain/discomfort, and anxiety/depression) using five response levels (i.e., no problems, slight problems, moderate problems, severe problems, or extreme problems) describing 234 health states.[24] The participants' health states obtained from EQ-5D-5L responses were converted into utility values using the Dutch tariff.[25] The utility values were used to calculate QALYs by means of the area under the curve method (i.e., the duration of a health state is multiplied by the utility related to that health state).

For peer review only - http://bmjopen.bmj.com/site/about/guidelines.xhtml

For peer review only

Cost outcomes

Intervention costs

The average costs of performing a CS reported by the participating hospitals was €5360. The intervention incurred additional suture material and additional operation time (3.9 minutes on average). The costs of additional resources were obtained from the academic and non-academic hospitals using a bottom-up approach. On average, the intervention resulted in additional costs of €95.79 per participant in academic hospitals and €71.14 per participant in non-academic hospitals (Supplementary Table S1).

Healthcare utilization and informal care costs

A specifically adapted version of the iMTA Medical Cost Questionnaire (iMCQ)[26] was used to measure healthcare utilization and care provided by family and/or friends (i.e., informal care) using 3-month and 6-month recall periods at 3 and 9 months of follow-up, respectively. The iMCQ is a standardized generic instrument for measuring medical costs including questions related to healthcare utilization and informal care.[26] Healthcare utilization was valued using prices from the Dutch costing guideline.[27] Healthcare ultilization costs included primary care costs (e.g., costs of visits to general practitioners, health professionals, and complementary healthcare providers), secondary care costs (e.g., costs of ambulatory hospital visits, visits to other healthcare organizations and admissions to the hospital), and medication costs. Secondary care costs were recorded after discharge from the hospital. The average costs of performing the CS included the operation and hospital stay until discharge and were, therefore, not included in the secondary care costs to avoid double-counting.

The informal care costs were based on the amount of time the participant needed help in performing household tasks or received care from family and/or friends, because of health problems. Dutch standard prices were used for informal care costs.[27] Medication use was valued using data from the Dutch Healthcare Institute (www.medicijnkosten.nl).[28]

Lost productivity costs

For peer review only - http://bmjopen.bmj.com/site/about/guidelines.xhtml

The iMTA Productivity Cost Questionnaire (iPCQ)[29] was used to measure self-reported sickness absenteeism from paid and unpaid work, and presenteeism using 3-month and 6-month recall periods at 3 and 9 months of follow-up, respectively. The iPCQ is a standardized generic questionnaire to measure productivity costs and it is applicable to national and international studies.[29] The friction cost approach (FCA) was used to calculate sickness absenteeism costs from paid work.[30] The FCA assumes that sickness absenteeism costs are limited to the period needed to replace an absent, sick worker (the friction period), which has been estimated to be 12 weeks (85 days) in the Netherlands.[27] Gender-specific estimates of the mean wages of the Dutch population were used to calculate sickness absenteeism costs from paid work.[27]

To measure sickness absenteeism from unpaid work, the participants were asked whether they had difficulty in performing unpaid work activities due to sickness (e.g., household tasks, childcare, voluntary work), and if that was the case, for how many hours.[29] Costs related to sickness absenteeism from unpaid work were valued using a shadow price for legally employing a domestic assistant.[29] To measure participants' level of presenteeism, participants rated how efficiently they worked while suffering from health complaints on a scale from 0 (I was unable to do anything) to 10 (I was able to do as much as usual). The resulting efficiency score was used to calculate presenteeism costs: Presenteeism costs = number of days working with complaints * [1 - (efficiency score / 10)] * number of working hours per day * gender-specific mean wage rates.[31]

Statistical analysis

The main analyses included all participants with a menstrual cycle (i.e., participants with amenorrhoea for any reason were excluded) at 9 months follow-up.

Analyses were performed according to the intention-to-treat principle using StataSE 16® (StataCorp LP, CollegeStation, TX, US). Multiple Imputation by Chained Equations (MICE), stratified by group allocation, was used to impute missing data. Variables associated with missingness and outcomes as well as potential confounders were included in the imputation model such as age, BMI, level of

For peer review only - http://bmjopen.bmj.com/site/about/guidelines.xhtml

education, parity, previous miscarriage, gestational age at CS, hypertension, diabetes, use of contraception, and breastfeeding. Predictive Mean Matching was used in the imputation procedure to account for the skewed distribution of the costs.[32] The number of imputations was increased until there was a loss of efficiency of ≤5%, resulting in twenty imputed datasets.[32 33] The twenty datasets were analyzed separately and estimates were pooled using Rubin's rules.[34] After multiple imputation, amenorrhoeic women were excluded from the analyses as a priori decided, because the outcome spotting days could not be evaluated in these women.[17]

Differences in costs and effects between treatment groups at nine months follow-up were estimated using seemingly unrelated regression analyses, which accounts for the correlation between costs and effects.[35] The intraclass correlation coefficient (ICC, i.e., the variation around the subjects belonging to the same hospital cluster divided by the total variance between hospitals)[36] was small (ICC=0.004). This means that hardly any of the variance in the outcome measure was accounted for by clustering at the level of the hospital. In addition to the small ICC, patients were randomised at the individual level and not at the hospital level. Therefore, multilevel analysis was not necessary. Incremental Cost-Effectiveness Ratios (ICERs) were calculated by dividing the difference in costs (i.e., total societal costs and total healthcare costs) between groups by the difference in effects.

Bias-corrected accelerated bootstrapping with 5,000 replications was used to estimate the joint uncertainty surrounding differences in costs and effects. Bootstrapped cost-effect pairs were plotted on cost-effectiveness planes (CE-planes).[37] Cost-effectiveness acceptability curves (CEACs) were estimated that show the probability of double-layer closure being cost-effective compared to single-layer closure for a range of willingness-to-pay (WTP) thresholds (i.e., the maximum amount of money society is willing to pay for a unit of effect gained).[38] For spotting days, we used a maximum WTP threshold of €253 per one day reduction. This threshold was based on the value of eight hours of paid work given the average productivity costs per working hour for women in the Netherlands (i.e., €31.6 per hour). For QALYs, we used a WTP threshold of €20,000/QALY gained recommended by the Dutch

For peer review only - http://bmjopen.bmj.com/site/about/guidelines.xhtml

Health Care Institute[27] and €23,420/QALY (equivalent of £20,000/QALY) recommended by the National Institute for Health and Clinical Excellence (NICE).[39]

Sensitivity analysis

Four sensitivity analyses (SA) were performed to assess the robustness of the results. SA1 consisted of a CEA including all women randomised (i.e., without excluding amenorrhoeic women) from both a societal and healthcare perspective for the QALY outcome. SA2 consisted of a complete case analysis from both a societal and healthcare perspective including only women without amenorrhoea for both spotting days and QALYs. Third, we performed per protocol analyses for both outcomes from both a societal and healthcare perspective (SA3). Finally, we performed a sensitivity analysis in which we adjusted for hormonal contraception and breastfeeding (exclusively or combined with formula) at 9 months of follow-up (SA4).

Patient and public involvement

The Dutch gynaecologic patients' association agreed upon the design of the study and the grant proposal for funding. They were not involved in outcome measures or recruitment, and they were not asked to give advice in the interpretation of the results. We will disseminate the study results to all participants, and to the public through popular science articles.

For peer review only

Results

Participants

In total, 2292 women undergoing a first CS were included. Of them, 1144 participants were randomised to single-layer and 1148 participants to double-layer closure of the uterine incision. In the single-layer group, 694 (60.7%) participants underwent planned CS, and in the double-layer group this was done in 705 (61.4%) participants.

In total, 672 women reported amenorrhoea (n=331 in control and n=341 in intervention group), resulting in 1620 women reported having menstrual blood loss over nine months of follow-up and were included in the main analyses (n=813 in control and n=807 in intervention group) [Figure 1]. Of all women included in the main analysis (n=1620), 95% had completed follow-up data for spotting days (n=1544, 774 in control group and 770 in intervention group). Within the total group (n=2292), complete follow-up data was available for 74% of QALYs (n=1696, 851 in control group and 845 in intervention group), for 72% of total healthcare costs (n=1653, 823 in control group and 830 in intervention group), and for 58% of total societal costs (n=1335, 671 in control group and 664 in intervention group)[Figure 1]. At baseline, no meaningful differences were found between both groups (Table 1). At 9 months follow-up, 12.1% of women in the single-layer arm and 17.1% of women in the double-layer arm were breastfeeding their child(ren). In the single-layer arm, 40.3% of the participants used hormonal contraceptives at 9 months follow-up, and in the double-layer arm this was 38.2%. Participants with complete follow-up were more likely to be nulliparous and to have a higher education level compared to participants without complete follow-up.

Effectiveness

There were no statistically significant differences between groups in spotting days (mean difference -0.045, 95% CI, -0.274, 0.364) and QALYs (mean difference -0.004, 95% CI, -0.013; 0.005) at nine months follow-up (Table 2).

Costs

For peer review only - http://bmjopen.bmj.com/site/about/guidelines.xhtml

The main contributors to total societal costs in both groups were lost productivity costs (€5426 in control group and €5719 in intervention group) and healthcare costs (€760 in control group and €728 in intervention group, Table 2). There were no differences in days of hospital stay between the groups.[18] Costs associated with absenteeism from unpaid work due to sickness (i.e., costs related to getting help to perform household tasks, childcare, voluntary work) was the largest contributor to lost productivity costs in both groups (€3513 in control group and €3820 in intervention group). Secondary care costs were the largest contributor to total healthcare costs in both groups (€393 in control, group and €315 in intervention group). During follow-up, there were no statistically significant differences between groups in total healthcare costs (€-32, 95% CI, -390; 150) and total societal costs (€292, 95% CI, -518; 1035, Table 2). Presenteeism costs were statistically significantly lower in the intervention group compared to the control group (€-92, 95% CI, -177; -11, Table 2).

Cost-effectiveness analysis

From a societal perspective, most bootstrapped cost-effect pairs (45%) were in the North-East quadrant of the CE-plane for spotting days (Table 3, Figure 2 [1A]). The probability of double-layer closure being cost-effective compared to single-layer was 0.25 at a WTP of €0/spotting day less and 0.26 at €253/spotting day less (Figure 2 [2A]). For QALYs, most of the bootstrapped cost-effect pairs (63%) was in the north-west quadrant of the CE-plane (Table 3, Figure 2 [1B]). The probability of double-layer closure being cost-effective compared to single-layer at both the Dutch WTP threshold of €20,000/QALY gained, and the UK WTP threshold of €23,420/QALY gained, was 0.22 from a societal perspective (Figure 2 [2B], supplementary table D).

From a healthcare perspective, bootstrapped cost-effect pairs were equally distributed among the eastern and western quadrants of the CE-plane for spotting days (Table 3, Figure 2 [1C]). This shows that uncertainty around costs and effects is large. The CEA curve presented in figure 2 [2C] shows that if the WTP for one spotting day less is €0, the probability of double-layer closure being cost-effective in comparison with single-layer was 0.57. This probability increases to 0.59 if the WTP is €253/spotting day less (Supplementary table S3). For QALYs, from a healthcare perspective, most of bootstrapped

For peer review only - http://bmjopen.bmj.com/site/about/guidelines.xhtml

cost-effect pairs were in the north-western quadrants of the CE-plane (Table 3, Figure 2 [1D]). The probability of double-layer closure being cost-effective compared to single-layer closure at the Dutch and UK WTP threshold (€20,000 and €23,420/QALY gained, respectively) was 0.41from a healthcare perspective (Table 3, Figure 2 [1D]).

The results of the sensitivity analyses were similar to those of the main analysis (Table 3).

For peer review only

Discussion

The results of this trial-based economic evaluation showed that double-layer uterine closure after a first CS did not significantly decrease spotting days nor improve QALYs compared to single-layer at nine months follow-up. In addition, total healthcare costs and societal costs related to double-layer closure did not significantly differ from single-layer closure. Low probabilities of double-layer closure being cost-effective in comparison with single-layer closure were found for all relevant willingness-to-pay thresholds. Therefore, double-layer closure was not considered cost-effective compared to single-layer closure after a first CS from a societal and a healthcare perspective.

<u>Comparison with previous studies</u>

The results of this economic evaluation are not in line with our hypothesis, which was based on previously conducted observational studies[5 6 40] and meta-analyses.[14] These showed that single-layer closure resulted in thinner residual myometrium and a higher proportion of large niches than double-layer closure. These sonographic findings, or surrogates, were suggested to lead to more postmenstrual spotting and, therefore, higher related costs. Although double-layer closure resulted in increased CS costs, these costs were neutralised by higher secondary care and presenteeism costs in the single-layer closure group, resulting in no overall difference in total health care costs or total societal costs. It is unclear how to explain the significantly lower presenteeism costs in the double-layer closure group, especially considering the similar estimates for all other cost categories. Since we did not correct for multiple testing, this statistically significant finding may be the result of chance.

To the best of our knowledge, this is the first study evaluating the cost-effectiveness of double-layer closure in comparison with single-layer closure after a CS. The largest study (n=15,935) on this topic mentioned possible cost savings but no cost-effectiveness analysis was performed.[41] The second largest study (n=3033) comparing single- versus double-layer closure in a factorial RCT hypothesised on a possible reduction in costs in their study protocol, since a CS is conducted so frequently that *'any difference in morbidity is likely to have significant cost and community effects'*. The authors found a

For peer review only

difference in operative time, though they did not discuss costs in the 2010 publication, and it is unlikely that a cost-effectiveness analysis is going to be performed.[42]

Based on the current and previous studies[43 44], we recommend to leave the choice of uterine closure technique with the preference of the surgeon. Previous studies reported only short-term maternal outcomes in the first few weeks after CS.[42-44] We confirmed these findings using a follow-up of nine months after CS. In addition, we showed that there is no difference in costs between the two types of closure. Our three-years follow-up results will show whether double-layer is superior compared to single-layer closure with regard to long-term outcomes. These outcomes include fertility outcomes, pregnancy complications and mode of delivery, as well as safety outcomes such as uterine dehiscence or rupture and related neonatal and maternal morbidity. In addition, a cost-effectiveness analysis for long-term outcomes will be performed as well. When superiority cannot be shown on the long-term either, guidelines should recommend to leave the uterine closure technique regarding single- versus double-layer up to the preference of the performing surgeon.

For peer review only

Strengths and limitations

This study was performed alongside a large multicenter randomised controlled superiority trial, which is considered the best vehicle for economic evaluations because it allows the prospective collection of cost and effect data and the use of patient level information for drawing inferences about additional costs and benefits of interventions.[16] Additionally, the CEA was conducted from a societal perspective meaning that all relevant costs for decision making (i.e., intervention, healthcare utilization, informal care and lost productivity costs) were included in the analysis.[22] Several sensitivity analyses were performed, to assess the robustness of our results, which resulted in similar results as compared to the main analyses.

However, one of the limitations of this study was that the cost questionnaires included retrospective self-reported questions over a 3-month and 6-month period, which may have caused recall bias. Nevertheless, we assume that this bias is equally distributed across the two groups and does,

For peer review only - http://bmjopen.bmj.com/site/about/guidelines.xhtml

therefore, does not impact the difference between groups. Although there is no gold standard for measuring lost productivity costs, we used a standardised instrument[29], which is considered best practice currently.[45] Another limitation is that generalizability of the results to healthcare systems in other countries may be limited, as they may adopt different usual practices and have different payment systems.[46] Additionally, generalizability may be impaired since in our study sample relatively many planned CS were performed compared to the Dutch average[47], probably resulting in an overall underestimation of niche related postmenstrual spotting.

Future research

It is important to realise that we have only evaluated the aspect of single-versus double-layer closure of the uterine incision in our trial. A CS consists of multiple steps and other aspects of the surgical technique used to perform a CS may also affect clinical outcomes and costs, and should therefore be subject to future research. Examples of uterine incision and repair are the level of hysterotomy (above or below the plica vesicouterina)[48] and inclusion or exclusion of the endometrium in the uterine suture.

Conclusions and policy implications

In conclusion, double-layer uterine closure is not cost-effective compared to single-layer uterine closure from both a societal and healthcare perspective. Thus, from a cost-effectiveness point of view, there is no reason to advocate double-layer over single-layer uterine closure. Long-term follow-up will show whether guidelines should be adapted based on obstetric and reproductive outcomes of double-layer closure compared to single-layer closure.

For peer review only - http://bmjopen.bmj.com/site/about/guidelines.xhtml

Acknowledgments

This study was performed with funding from ZonMw: The Netherlands Organisation for Health Research and Development (project number 843002605). We would like to thank all participants of the 2Close study. Additionally, we would like to thank the Departments of Obstetrics and Gynaecology of all participating hospitals, with special thanks to all research nurses and research midwives for their contribution in data collection.

The following are members of the 2Close study group: WM (Marchien) van Baal, Erik van Beek, Mireille N Bekker, Ângela Jornada Ben, Karin de Boer, Elisabeth MA Boormans, Judith E Bosmans, Hugo WF van Eijndhoven, Mohamed El Alili, AH (Hanneke) Feitsma, Christianne JM de Groot, Majoie Hemelaar, Wouter JK Hehenkamp, Wietske Hermes, Esther Hink, Judith AF Huirne, Anjoke JM Huisjes, CAH (Ineke) Janssen, Kitty Kapiteijn, Mesrure Kaplan, Paul JM van Kesteren, Judith OEH van Laar, Josje Langenveld, Wouter J Meijer, Angèle LM Oei, Eva Pajkrt, Dimitri NM Papatsonis, Celine M Radder, Robbert JP Rijnders, HCJ (Liesbeth) Scheepers, Daniela H Schippers, Nico WE Schuitemaker, Sanne I Stegwee, Marieke Sueters, Harry Visser, Huib AAM van Vliet, LHM (Marloes) de Vleeschouwer, Lucet F van der Voet

Contributors: SIS collected data, interpreted data and drafted the report. AJB and MEA analysed and interpreted data and drafted the report. LFvdV designed the study, collected data and revised the first draft of the report critically. CJMdG designed the study and revised the first draft of the report critically. JEB designed the study, interpreted data and participated in drafting and revising the report. JAFH designed the study, was principle investigator, interpreted the data and participated in drafting and revising the report. All other members of the 2Close study group (WMvB, EvB, MNB, KdB, EMAB, HWFvE, AHF, MH, WJKH, WH, EH, AJMH, CAHJ, KK, MK, PJMvK, JOEHvL, JL, WJM, ALMO, EP, DNMP, CMR, RJPR, HCJS, DHS, NWES, MS, HV, HAAMvV, LHMdV) agreed with the design of the trial, participated in data collection as local investigators, and revised the draft paper. All authors and collaborators approved the final version of the manuscript.

For peer review only

Funding: This study was performed with funding from ZonMw: The Netherlands Organisation for Health Research and Development (project number 843002605). The funder of the study approved the study protocol. The funder had no role in study design, data collection, data analysis, data interpretation, or writing of the manuscript. The corresponding author had full access to all the data in the study and had final responsibility for the decision to submit for publication.

Competing interests: JAFH received grants from ZonMw, during the conduct of the study; and reports grants from Samsung, grants from PlantTec Medical GmbH, and received a fee from Olympus, all outside the submitted work. CJMdG received a grant from ZonMw outside the submitted work. The other authors declare no competing interests.

Ethical approval: The study was approved by the Institutional Review Board (IRB) of Amsterdam UMC – location VU University medical centre in December 2015 (registration number 2015.462) and by the boards of all participating hospitals before start of inclusion. No substantial changes were made to the protocol after commencement of the trial. All participants provided written informed consent before taking part in the study.

Data sharing: De-identified individual participant data collected during the 2Close trial will be shared at one year after publication of the long-term results on request (j.huirne@amsterdamumc.nl). Approval of a proposal will be necessary before data will be shared. To gain access, requesters will need to sign an agreement form and confirm that data will be used for the purpose for which access was granted.

The corresponding and lead author (JAFH) affirms that the manuscript is an honest, accurate, and transparent account of the study being reported; no important aspects of the study have been omitted; any discrepancies from the study as originally planned in the registered study protocol have been explained. The corresponding author (JAFH) had the final responsibility for the decision to submit for publication.

For peer review only - http://bmjopen.bmj.com/site/about/guidelines.xhtml

439 This is an open access article distributed in accordance with the Creative Commons Attribution Non Commercial (CC BY-NC 4.0) license, which permits others to distribute, remix, adapt, build upon this work non-commercially, and license their derivative works on different terms, provided the original work is properly cited and the use is non-commercial. See: http://creativecommons.org/licenses/by-nc/4.0.

For peer review only

For peer review only - http://bmjopen.bmj.com/site/about/guidelines.xhtml

References

1. Boerma T, Ronsmans C, Melesse DY, et al. Global epidemiology of use of and disparities in caesarean sections. *Lancet* 2018;392(10155):1341-48. doi: 10.1016/s0140-6736(18)31928-7 [published Online First: 2018/10/17]
2. Betran AP, Ye J, Moller AB, et al. The Increasing Trend in Caesarean Section Rates: Global, Regional and National Estimates: 1990-2014. *PLoS One* 2016;11(2):e0148343. doi: 10.1371/journal.pone.0148343 [published Online First: 2016/02/06]
3. Marshall NE, Fu R, Guise JM. Impact of multiple cesarean deliveries on maternal morbidity: a systematic review. *Am J Obstet Gynecol* 2011;205(3):262.e1-8. doi: 10.1016/j.ajog.2011.06.035 [published Online First: 2011/11/11]
4. Wang CB, Chiu WW, Lee CY, et al. Cesarean scar defect: correlation between Cesarean section number, defect size, clinical symptoms and uterine position. *Ultrasound Obstet Gynecol* 2009;34(1):85-9. doi: 10.1002/uog.6405 [published Online First: 2009/07/01]
5. Bij de Vaate AJ, Brolmann HA, van der Voet LF, et al. Ultrasound evaluation of the Cesarean scar: relation between a niche and postmenstrual spotting. *Ultrasound Obstet Gynecol* 2011;37(1):93-9. doi: 10.1002/uog.8864 [published Online First: 2010/10/30]
6. van der Voet LF, Bij de Vaate AM, Veersema S, et al. Long-term complications of caesarean section. The niche in the scar: a prospective cohort study on niche prevalence and its relation to abnormal uterine bleeding. *BJOG* 2014;121(2):236-44. doi: 10.1111/1471-0528.12542 [published Online First: 2014/01/01]
7. Roberge S, Boutin A, Chaillet N, et al. Systematic review of cesarean scar assessment in the nonpregnant state: imaging techniques and uterine scar defect. *Am J Perinatol* 2012;29(6):465-71. doi: 10.1055/s-0032-1304829 [published Online First: 2012/03/09]
8. Bij de Vaate AJ, van der Voet LF, Naji O, et al. Prevalence, potential risk factors for development and symptoms related to the presence of uterine niches following Cesarean section: systematic review. *Ultrasound Obstet Gynecol* 2014;43(4):372-82. doi: 10.1002/uog.13199 [published Online First: 2013/09/03]
9. van der Voet LF, Vervoort AJ, Veersema S, et al. Minimally invasive therapy for gynaecological symptoms related to a niche in the caesarean scar: a systematic review. *BJOG* 2014;121(2):145-56. doi: 10.1111/1471-0528.12537 [published Online First: 2014/01/01]
10. Eurostat. Surgical operations and procedures statistics - statistics explained. . 03 Oct 2019. https://ec.europa.eu/eurostat/statisticsexplained/ (accessed 18 Nov 2019).
11. Caughey AB, Wood SL, Macones GA, et al. Guidelines for intraoperative care in cesarean delivery: Enhanced Recovery After Surgery Society Recommendations (Part 2). *Am J Obstet Gynecol* 2018;219(6):533-44. doi: 10.1016/j.ajog.2018.08.006 [published Online First: 2018/08/18]
12. NICE. Caesarean section - clinical guideline. . 01 Nov 2011. https://www.nice.org.uk/guidance/cg132 (accessed 19 Nov 2019).
13. Roberge S, Demers S, Berghella V, et al. Impact of single- vs double-layer closure on adverse outcomes and uterine scar defect: a systematic review and metaanalysis. *Am J Obstet Gynecol* 2014;211(5):453-60. doi: 10.1016/j.ajog.2014.06.014 [published Online First: 2014/06/10]
14. Stegwee SI, Jordans I, van der Voet LF, et al. Uterine caesarean closure techniques affect ultrasound findings and maternal outcomes: a systematic review and meta-analysis. *BJOG* 2018;125(9):1097-108. doi: 10.1111/1471-0528.15048 [published Online First: 2017/12/08]
15. Sevket O, Ates S, Molla T, et al. Hydrosonographic assessment of the effects of 2 different suturing techniques on healing of the uterine scar after cesarean delivery. *Int J Gynaecol Obstet* 2014;125(3):219-22. doi: 10.1016/j.ijgo.2013.11.013 [published Online First: 2014/04/01]

For peer review only

For peer review only - http://bmjopen.bmj.com/site/about/guidelines.xhtml

16. Ramsey SD, Willke RJ, Glick H, et al. Cost-effectiveness analysis alongside clinical trials II-An ISPOR Good Research Practices Task Force report. *Value Health* 2015;18(2):161-72. doi: 10.1016/j.jval.2015.02.001 [published Online First: 2015/03/17]

17. Stegwee SI, Jordans IPM, van der Voet LF, et al. Single- versus double-layer closure of the caesarean (uterine) scar in the prevention of gynaecological symptoms in relation to niche development - the 2Close study: a multicentre randomised controlled trial. *BMC Pregnancy Childbirth* 2019;19(1):85. doi: 10.1186/s12884-019-2221-y [published Online First: 2019/03/06]

18. Stegwee S, van der Voet L, Ben A, et al. Effect of single- versus double-layer uterine closure during caesarean section on postmenstrual spotting (2Close): multicentre, double-blind, randomised controlled superiority trial. *BJOG* 2020 doi: 10.1111/1471-0528.16472

19. Husereau D, Drummond M, Petrou S, et al. Consolidated Health Economic Evaluation Reporting Standards (CHEERS) statement. *BMJ* 2013;346:f1049. doi: 10.1136/bmj.f1049 [published Online First: 2013/03/27]

20. Jordans IPM, de Leeuw RA, Stegwee SI, et al. Sonographic examination of uterine niche in non-pregnant women: a modified Delphi procedure. *Ultrasound Obstet Gynecol* 2019;53(1):107-15. doi: 10.1002/uog.19049 [published Online First: 2018/03/15]

21. Whitehead SJ, Ali S. Health outcomes in economic evaluation: the QALY and utilities. *Br Med Bull* 2010;96:5-21. doi: 10.1093/bmb/ldq033 [published Online First: 2010/11/03]

22. van Dongen JM, van Wier MF, Tompa E, et al. Trial-based economic evaluations in occupational health: principles, methods, and recommendations. *J Occup Environ Med* 2014;56(6):563-72. doi: 10.1097/jom.0000000000000165 [published Online First: 2014/05/24]

23. Gray A, Clarke PM, Wolstenholme JL, et al. Applied Methods of Cost-effectiveness Analysis in Healthcare: Oxford University Press. http://econpapers.repec.org/bookchap/oxpobooks/9780199227280.htm (accessed Oct 28, 2019), 2010.

24. Herdman M, Gudex C, Lloyd A, et al. Development and preliminary testing of the new five-level version of EQ-5D (EQ-5D-5L). *Qual Life Res* 2011;20(10):1727-36. doi: 10.1007/s11136-011-9903-x [published Online First: 2011/04/12]

25. Versteegh MM, Vermeulen KM, Evers SMAA, et al. Dutch Tariff for the Five-Level Version of EQ-5D. *Value Health* 2016;19(4):343-52. doi: 10.1016/j.jval.2016.01.003 [published Online First: 2016/06/22]

26. Bouwmans C, Hakkaart-van Roijen L, Koopmanschap M, et al. Manual iMTA medical cost questionnaire (iMCQ). *Rotterdam: iMTA, Erasmus Universiteit Rotterdam* 2013

27. Hakkaart - van Roijen L, van der Linden N, Bouwmans CAM, et al. Costing manual: Methodology of costing research and reference prices for economic evaluations in healthcare. In: Volksgezondheid WeS, ed. Nederland: Zorginstituut Nederland, 2016.

28. VWS. Medicijnkosten - Zorginstituut Nederland - Ministerie van Volksgezondheid, Welzijn en Sport, 2018.

29. Bouwmans C, Krol M, Severens H, et al. The iMTA Productivity Cost Questionnaire: A Standardized Instrument for Measuring and Valuing Health-Related Productivity Losses. *Value Health* 2015;18(6):753-8. doi: 10.1016/j.jval.2015.05.009 [published Online First: 2015/09/28]

30. Koopmanschap MA, Rutten FF, van Ineveld BM, et al. The friction cost method for measuring indirect costs of disease. *J Health Econ* 1995;14(2):171-89. [published Online First: 1995/05/08]

31. Krol M, Brouwer W. How to estimate productivity costs in economic evaluations. *Pharmacoeconomics* 2014;32(4):335-44. doi: 10.1007/s40273-014-0132-3 [published Online First: 2014/02/08]

32. White IR, Royston P, Wood AM. Multiple imputation using chained equations: Issues and guidance for practice. *Stat Med* 2011;30(4):377-99. doi: 10.1002/sim.4067 [published Online First: 2011/01/13]

For peer review only - http://bmjopen.bmj.com/site/about/guidelines.xhtml

33. Madley-Dowd P, Hughes R, Tilling K, et al. The proportion of missing data should not be used to guide decisions on multiple imputation. *J Clin Epidemiol* 2019;110:63-73. doi: 10.1016/j.jclinepi.2019.02.016 [published Online First: 2019/03/18]
34. Rubin DB. Multiple Imputation for Nonresponse in Surveys: John Wiley & Sons 2004.
35. Willan AR, Briggs AH, Hoch JS. Regression methods for covariate adjustment and subgroup analysis for non-censored cost-effectiveness data. *Health Econ* 2004;13(5):461-75. doi: 10.1002/hec.843 [published Online First: 2004/05/06]
36. Twisk JWR. Applied Longitudinal Data Analysis for Epidemiology: A Practical Guide: Cambridge University Press 2013.
37. Black WC. The CE plane: a graphic representation of cost-effectiveness. *Med Decis Making* 1990;10(3):212-4. doi: 10.1177/0272989x9001000308 [published Online First: 1990/07/01]
38. Fenwick E, O'Brien BJ, Briggs A. Cost-effectiveness acceptability curves--facts, fallacies and frequently asked questions. *Health Econ* 2004;13(5):405-15. doi: 10.1002/hec.903 [published Online First: 2004/05/06]
39. McCabe C, Claxton K, Culyer AJ. The NICE cost-effectiveness threshold: what it is and what that means. *Pharmacoeconomics* 2008;26(9):733-44. doi: 10.2165/00019053-200826090-00004 [published Online First: 2008/09/05]
40. Hayakawa H, Itakura A, Mitsui T, et al. Methods for myometrium closure and other factors impacting effects on cesarean section scars of the uterine segment detected by the ultrasonography. *Acta Obstet Gynecol Scand* 2006;85(4):429-34. doi: 10.1080/00016340500430436 [published Online First: 2006/04/14]
41. Abalos E, Addo V, Brocklehurst P, et al. Caesarean section surgical techniques: 3 year follow-up of the CORONIS fractional, factorial, unmasked, randomised controlled trial. *Lancet* 2016;388(10039):62-72. doi: 10.1016/s0140-6736(16)00204-x [published Online First: 2016/05/09]
42. The CAESAR study collaborative group. Caesarean section surgical techniques: a randomised factorial trial (CAESAR). *BJOG* 2010;117(11):1366-76. doi: 10.1111/j.1471-0528.2010.02686.x [published Online First: 2010/09/16]
43. Dodd JM, Anderson ER, Gates S, et al. Surgical techniques for uterine incision and uterine closure at the time of caesarean section. *Cochrane Database Syst Rev* 2014(7):CD004732. doi: 10.1002/14651858.CD004732.pub3 [published Online First: 2014/07/23]
44. Abalos E, Addo V, Brocklehurst P, et al. Caesarean section surgical techniques (CORONIS): a fractional, factorial, unmasked, randomised controlled trial. *Lancet* 2013;382(9888):234-48. doi: 10.1016/s0140-6736(13)60441-9 [published Online First: 2013/06/01]
45. Versteegh M, Knies S, Brouwer W. From Good to Better: New Dutch Guidelines for Economic Evaluations in Healthcare. *Pharmacoeconomics* 2016;34(11):1071-74. doi: 10.1007/s40273-016-0431-y [published Online First: 2016/10/22]
46. Drummond M, Barbieri M, Cook J, et al. Transferability of economic evaluations across jurisdictions: ISPOR Good Research Practices Task Force report. *Value Health* 2009;12(4):409-18. doi: 10.1111/j.1524-4733.2008.00489.x [published Online First: 2009/11/11]
47. Perined. Perinatale zorg in Nederland anno 2018: landelijke perinatale cijfers en duiding, 2019.
48. Vikhareva O, Rickle GS, Lavesson T, et al. Hysterotomy level at Cesarean section and occurrence of large scar defects: a randomized single-blind trial. *Ultrasound Obstet Gynecol* 2019;53(4):438-42. doi: 10.1002/uog.20184 [published Online First: 2018/11/30]

For peer review only

For peer review only - http://bmjopen.bmj.com/site/about/guidelines.xhtml

Figures

Figure 1: Trial profile

*Logistical reasons, computer randomisation issues, passing through the allocated method to operating gynaecologist, or participant not traceable after randomisation.

Figure 2: Cost-effectiveness planes and cost acceptability curves from a societal and healthcare perspective comparing double- to single-layer uterine closure

[1] Cost-effectiveness plane (CE-plane) showing the incremental cost-effectiveness ratio point estimate (ICER, red dot) and the distribution of the 5000 replications of the bootstrapped cost-effective pairs (blue dots). [2] Cost-acceptability curve (CEAC) indicating the probability of the double-layer uterine closure being cost-effective compared to the single layer (y-axis) for different willingness-to-pay (WTP) thresholds per unit of effect gained (x-axis).
[1A] CE-plane for spotting days from a societal perspective showing that most of bootstrapped cost-effect pairs were equally distributed across CE-plane quadrants representing high uncertainty around ICER.
[2A] CEAC for spotting days from a societal perspective indicating a steady 0.2 probability of the double-layer uterine closure being cost-effective compared to the single-layer for different WTP thresholds per fewer spotting days.
[1B] CE-plane for QALYs from a societal perspective showing that most of the bootstrapped cost-effect pairs were in the north quadrants (i.e., higher costs) and western quadrants where the double-layer uterine closure was less effective compared to the single-layer.
[2B] CEAC for QALYs from a societal perspective indicating a probability of the double-layer uterine closure being cost-effective around 0.2 for different WTP thresholds per QALY gained.
[1C] CE-plane for spotting days from a healthcare perspective showing that most of the bootstrapped cost-effect pairs were in south quadrants, where the double-layer uterine closure was less costly compared to the single-layer, but they are equally distributed across the east and western quadrants representing high uncertainty around the effectiveness of the double-layer uterine closure compared to the single-closure.
[2C] CEAC for spotting days from a healthcare perspective indicating a steady 0.6 probability of the double-layer uterine closure being cost-effective compared to the single layer for different WTP thresholds per fewer spotting days.
[1D] CE-plane for QALYs from a healthcare perspective showing that most of the bootstrapped cost-effect pairs were in the south quadrants (i.e., lower costs) and western quadrants where the double-layer uterine closure was less effective compared to the single-layer.
[2D] CEAC for QALYs from a healthcare perspective indicating that the probability of the double-layer uterine closure being cost-effective compared to the single-layer decreased with an increasing of the different WTP thresholds per QALY gained because healthcare costs were on average lower in the intervention group while it is less effective compared to the usual practice.

For peer review only - http://bmjopen.bmj.com/site/about/guidelines.xhtml

Tables

Table 1: Baseline characteristics of women without amenorrhoea in the control group and intervention group.

	Single-layer (N=813)*	Double-layer (N=807)†
Age, years	32.1 (4.7)	32.0 (4.6)
Level of education		
Low	50 (6.5)	54 (7.1)
Middle	263 (34.2)	242 (31.8)
High	452 (58.7)	457 (60.1)
Nulliparous women	568 (73.9)	578 (75.9)
BMI kg/m^2	26.4 (4.5)	26.7 (4.9)
Smoking habit	44 (5.7)	37 (4.9)
Hypertension	146 (19.0)	127 (16.7)
Diabetes mellitus	89 (11.6)	66 (8.6)
Gestational age	38.6 (2.4)	38.6 (2.3)
Previous miscarriage	255 (33.2)	221 (29.0)
Previous ectopic pregnancy	10 (1.3)	12 (1.6)
Planned CS	504 (62.0)	503 (62.3)

Data are mean (SD), or n (%). N is equal to the total number of patients in the group. BMI, body mass index. *5.5% missing data for all variables, except 'planned CS' (0%). †5.9% missing data for all variables, except 'planned CS' (0%).

For peer review only

Table 2: Multiply imputed mean effects and costs by group and mean difference at nine months follow-up in women without amenorrhoea

	**Single-layer (n=813)**	**Double-layer (n=807)**	**Mean difference* (95% CI)**
**Effects**			
Spotting days	1.44 (0.11)	1.40 (0.11)	-0.045 (-0.364 to 0.274)
QALYs gained	0.663 (0.003)	0.659 (0.003)	-0.004 (-0.013 to 0.005)
**Costs**			
Intervention costs†	0	76 (0.31)	76 (75 to 76)
Primary care costs	253 (16)	249 (17)	-4 (-49 to 43)
Secondary care costs	393 (74)	315 (47)	-78 (-295 to 43)
Medication costs	114 (140)	88 (26)	-25 (-257 to 72)
Total healthcare costs‡	760 (164)	728 (61)	-32 (-390 to 150)
Informal care costs	76 (17)	123 (37)	47 (-10 to 138)
Absenteeism costs at paid work	613 (43)	675 (47)	62 (-51 to 1979)
Absenteeism costs at unpaid work	3513 (224)	3820 (261)	307 (-331 to 998)
Presenteeism costs	463 (68)	371 (30)	-92 (-177 to -11)
Total lost productivity costs	4589 (251)	4866 (279)	277 (-466 to 1021)
**Total societal costs§**	5426 (305)	5719 (311)	292 (-518 to 1096)

Data are mean (SE). CI=confidence interval, QALY=quality-adjusted life-years. Multiple imputation model consisted of age, education level, parity, body mass index, smoking habit, hypertensive disorder, diabetic status, gestational complications, gestational age, previous miscarriage or ectopic pregnancies , use of contraception, breastfeeding, and self-reported menstrual blood loss. *Cost and effect differences at nine months follow-up were estimated using seemingly unrelated regression analyses.[35] †Additional intervention costs to perform double-layer, excluding the average costs for performing a caesarean section (€5360,-). ‡The sum of intervention, primary care, secondary care and medication costs. §The sum of total healthcare costs, informal care costs, and lost productivity costs.

Primary care: costs of visits to general practitioners, health professionals, and complementary healthcare providers. Secondary care: costs of ambulatory hospital visits, visits to other healthcare organizations and hospital admissions. Medication costs: costs of medication use after discharge from the hospital. Informal care costs: costs of received care from family and/or friends due to health problems. Absenteeism costs at paid work: costs of sickness absenteeism from paid work. Absenteeism costs at unpaid work: costs of absenteeism from unpaid work activities (e.g., household tasks, childcare, voluntary work). Presenteeism costs: costs of working while suffering from health complaints.

For peer review only

Table 3: Results of the cost-effectiveness analysis

Effect outcome*	Cost difference, € (95% CI)	Effect difference (95% CI)	ICER €/ effect gained	Distribution of the cost-effectiveness plane			
				North-East	South-East	South-West	North-West
**Main analysis – Societal perspective**							
Spotting days	292 (-518 to 1096)	0.045 (-0.274 to 0.364)	6480	45%	16%	9%	30%
QALY	292 (-518 to 1096)	-0.004 (-0.013 to 0.005)	-73632	12%	8%	17%	63%
**Main analysis – Healthcare perspective**							
Spotting days	-32 (-390 to 150)	0.045 (-0.274 to 0.364)	-704	29%	32%	19%	20%
QALY	-32 (-390 to 150)	-0.004 (-0.013 to 0.005)	8004	9%	11%	39%	41%
**SA1 – including all women randomised - Societal perspective**							
QALY	147 (-729 to 872)	-0.005 (-0.013 to 0.003)	-29192	5%	6%	30%	59%
**SA1 – including all women randomised - Healthcare perspective**							
QALY	-240 (-1237 to 87)	-0.004 (-0.013 to 0.004)	55741	3%	12%	65%	20%
**SA2 – complete case analysis – Societal perspective**							
Spotting days	380 (-451 to 1273)	0.149 (-0.452 to 0.138)	2554	74%	13%	2%	11%
QALY	397 (-427 to 1275)	-0.006 (-0.017 to 0.003)	-64515	22%	4%	12%	62%
**SA2 - complete case analysis – Healthcare perspective**							
Spotting days	-9 (-251 to 174)	0.186 (-0.486 to 0.103)	-46	54%	37%	4%	5%
QALY	1 (-255 to 177)	-0.007 (-0.016 to 0.002)	-80	23%	4%	34%	39%
**SA3 – per protocol analysis - Societal perspective**							
Spotting days	59 (-750 to 859)	0.033 (-0.288 to 0.354)	1774	31%	27%	18%	24%
QALY	59 (-750 to 859)	0.003 (-0.006 to 0.012)	19427	46%	28%	16%	10%
**SA3 – per protocol analysis – Healthcare perspective**							
Spotting days	-50 (-403 to 132)	0.033 (-0.288 to 0.354)	-1515	24%	34%	23%	19%
QALY	-50 (-403 to 132)	0.003 (-0.006 to 0.012)	-16593	34%	40%	16%	10%
**SA4 – Main analysis adjusted – Societal Perspective**							
Spotting days	304 (-510 to 1111)	0.037 (-0.284 to 0.359)	8152	43%	15%	10%	32%
QALY	304 (-510 to 1111)	-0.003 (-0.012 to 0.006)	-97125	15%	10%	15%	60%
**SA4 – Main analysis adjusted – Healthcare perspective**							
Spotting days	-26 (-392 to 161)	0.037 (-0.284 to 0.359)	-700	29%	29%	19%	23%
QALY	-26 (-392 to 161)	-0.003 (-0.012 to 0.006)	8340	12%	13%	34%	41%

Data are mean (95% CI). *The effect outcome "Spotting days" was multiplied by -1 in the cost-effectiveness analysis to keep the CE-plane interpretable. CI=confidence interval, ICER=incremental cost-effectiveness ratio, QALY=quality-adjusted life-year, SA=sensitivity analysis.

Main analysis: CEA from a societal and a healthcare perspective for spotting days and QALY including only women without amenorrhoea (total=1620, control n=813, intervention n=807).

SA1: CEA from a societal and a healthcare perspective for QALY, including all women randomised in the study after multiple imputation (i.e., without excluding amenorrhoeic women, n=2292)[Supplementary Table S2].

SA2: CEA from a societal perspective using complete cases for spotting days and total societal costs (total=1065, control n=544, intervention n=521) including only women without amenorrhoea (n=1620).

SA2: CEA from a societal perspective using complete cases for QALY and total societal costs (total=1057, control n=541, intervention n=516) including only women without amenorrhoea (n=1620).

SA2: CEA from a healthcare perspective using complete cases for spotting days and total healthcare costs (total=1315, control n=662, intervention n=653) including only women without amenorrhoea (n=1620).

For peer review only - http://bmjopen.bmj.com/site/about/guidelines.xhtml

SA2: CEA from a healthcare perspective using complete cases for QALY and total healthcare costs (total=1310, control n=657, intervention n=653) including only women without amenorrhoea (n=1620).
SA3: per-protocol analysis for spotting days and QALY from a societal perspective (total=1620, control n=828, intervention n=792) including only women without amenorrhoea (n=1620).
SA3: per-protocol analysis for spotting days and QALY from a healthcare perspective (total=1620, control n=828, intervention n=792) including only women without amenorrhoea (n=1620).
SA4: main analysis adjusted for the use of contraception and breastfeeding during follow-up from a societal and a healthcare perspective (total=1620, control n=813, intervention n=807)

For peer review only - http://bmjopen.bmj.com/site/about/guidelines.xhtml

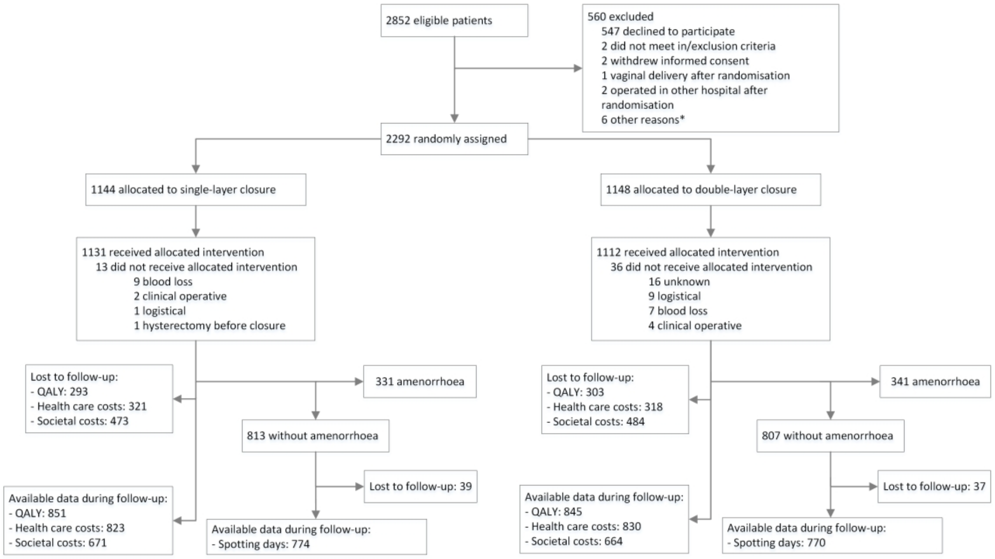

Figure 1: Trial profile

*Logistical reasons, computer randomisation issues, passing through the allocated method to operating gynaecologist, or participant not traceable after randomisation.

287x161mm (300 x 300 DPI)

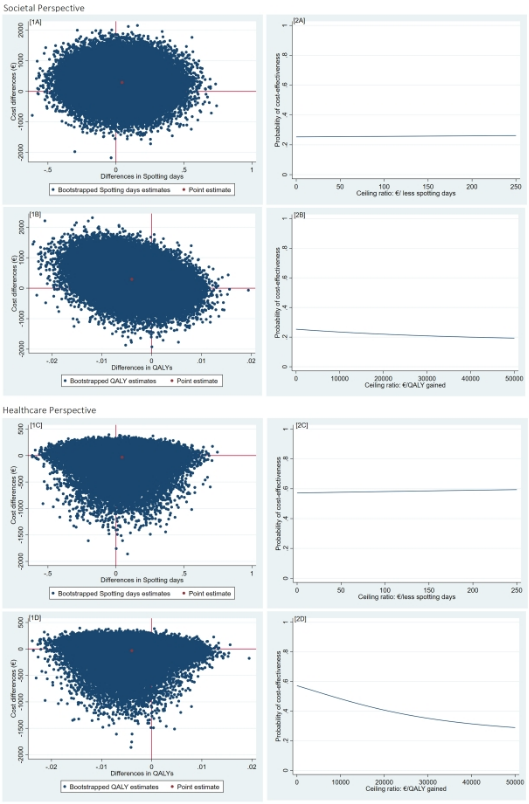

Figure 2: Cost-effectiveness planes and cost acceptability curves from a societal and healthcare perspective comparing double- to single-layer uterine closure

[1] Cost-effectiveness plane (CE-plane) showing the incremental cost-effectiveness ratio point estimate (ICER, red dot) and the distribution of the 5000 replications of the bootstrapped cost-effective pairs (blue dots). [2] Cost-acceptability curve (CEAC) indicating the probability of the double-layer uterine closure being cost-effective compared to the single layer (y-axis) for different willingness-to-pay (WTP) thresholds per unit of effect gained (x-axis).
[1A] CE-plane for spotting days from a societal perspective showing that most of bootstrapped cost-effect pairs were equally distributed across CE-plane quadrants representing high uncertainty around ICER.
[2A] CEAC for spotting days from a societal perspective indicating a steady 0.2 probability of the double-layer uterine closure being cost-effective compared to the single-layer for different WTP thresholds per fewer spotting days.
[1B] CE-plane for QALYs from a societal perspective showing that most of the bootstrapped cost-effect pairs

were in the north quadrants (i.e., higher costs) and western quadrants where the double-layer uterine closure was less effective compared to the single-layer.

[2B] CEAC for QALYs from a societal perspective indicating a probability of the double-layer uterine closure being cost-effective around 0.2 for different WTP thresholds per QALY gained.

[1C] CE-plane for spotting days from a healthcare perspective showing that most of the bootstrapped cost-effect pairs were in south quadrants, where the double-layer uterine closure was less costly compared to the single-layer, but they are equally distributed across the east and western quadrants representing high uncertainty around the effectiveness of the double-layer uterine closure compared to the single-closure.

[2C] CEAC for spotting days from a healthcare perspective indicating a steady 0.6 probability of the double-layer uterine closure being cost-effective compared to the single layer for different WTP thresholds per fewer spotting days.

[1D] CE-plane for QALYs from a healthcare perspective showing that most of the bootstrapped cost-effect pairs were in the south quadrants (i.e., lower costs) and western quadrants where the double-layer uterine closure was less effective compared to the single-layer.

[2D] CEAC for QALYs from a healthcare perspective indicating that the probability of the double-layer uterine closure being cost-effective compared to the single-layer decreased with an increasing of the different WTP thresholds per QALY gained because healthcare costs were on average lower in the intervention group while it is less effective compared to the usual practice.

Supplementary appendix

Appendix S1. List of collaborators.

**Hospital**	**Site principal investigator**
Amphia hospital, Breda	Dimitri NM Papatsonis
Amsterdam UMC, Univ of Amsterdam, Amsterdam	Eva Pajkrt
Amsterdam UMC, VU University, Amsterdam	Wouter JK Hehenkamp
Bernhoven hospital, Uden	Angèle LM Oei
Birth Centre Wilhelmina Children Hospital/University Medical Centre Utrecht, Utrecht	Mireille N Bekker
Canisius-Wilhelmina hospital, Nijmegen	Daniela H Schippers
Catharina hospital, Eindhoven	Huib AAM van Vliet
Deventer ziekenhuis, Deventer	Lucet van der Voet
Diakonessenhuis, Utrecht	Nico WE Schuitemaker
Dijklander hospital – location Hoorn	Majoie Hemelaar
Flevo hospital, Almere	WM (Marchien) van Baal
Gelre hospital – location Apeldoorn	Anjoke JM Huisjes
Gelre hospital – location Zutphen	Wouter J Meijer
Groene Hart hospital, Gouda	CAH (Ineke) Janssen
Haaglanden Medical Centre – Westeinde hospital, Den Haag	Wietske Hermes
Haga hospital, Den Haag	AH (Hanneke) Feitsma
Isala clinics, Zwolle	Hugo WF van Eijndhoven
Jeroen Bosch hospital, 's-Hertogenbosch	Robbert JP Rijnders
Leiden University Medical Centre, Leiden	Marieke Sueters
Maastricht University Medical Centre, Research school 'GROW', Maastricht	HCJ (Liesbeth) Scheepers
Máxima Medical Centre, Veldhoven	Judith OEH van Laar
Meander Medical Centre, Amersfoort	Elisabeth MA Boormans
OLVG-oost, Amsterdam	Paul JM van Kesteren
OLVG-west, Amsterdam	Celine M Radder
Radboud University Nijmegen Medical Centre, Nijmegen	Esther Hink
Reinier de Graaf hospital, Delft	Kitty Kapiteijn
Rijnstate hospital, Arnhem	Karin de Boer
Röpcke-Zweers hospital, Hardenberg	Mesrure Kaplan
Sint Antonius Hospital, Nieuwegein	Erik van Beek
Sint Franciscus Hospital, Rotterdam	LHM (Marloes) de Vleeschouwer
Tergooi hospital, Blaricum	Harry Visser
Zuyderland Medical Centre, Heerlen	Josje Langenveld

For peer review only

Supplementary tables

Table S1: Costs of the intervention per participant

Resources	Units	Unit price, €	Costs, €
Additional suture material	One piece	6.14	6.14
Additional operation time, academic hospital (3.9 minutes)	Hour	1379.27	89.65
Total costs per participant in academic hospital			95.79
Additional operation time, non-academic hospital (3.9 minutes)	Hour	1000	65.00
Total costs per participant in non-academic hospital			71.14

Table S2: Description of follow-up complete cases and missing cases by effect and cost outcomes without excluding cases of amenorrhoea

	Complete cases			Missing cases (%)		
	Intervention	*Control*	*Total*	*Intervention*	*Control*	*Total*
Spotting days	936	940	1876	212 (23)	204 (22)	416 (22)
QALY	845	851	1696	303 (36)	293 (34)	596 (35)
Primary care costs	913	924	1837	235 (26)	220 (24)	455 (25)
Secondary care costs	838	833	1671	310 (37)	311 (37)	621 (37)
Medication costs	834	825	1659	314 (38)	319 (39)	633 (38)
Total healthcare costs	830	823	1653	318 (38)	321 (39)	639 (39)
Informal care costs	913	924	1837	235 (26)	220 (24)	455 (25)
Absenteeism costs at work	852	849	1701	296 (35)	295 (35)	591 (35)
Absenteeism costs at unpaid work	905	921	1826	243 (27)	223 (24)	466 (25)
Presenteeism costs	680	690	1370	468 (69)	454 (66)	922 (67)
Total lost productivity costs	677	689	1366	471 (69)	455 (66)	926 (68)
Total societal costs	664	671	1335	484 (73)	473 (70)	957 (72)

Table S3: Probability of the intervention being cost-effective at different willingness-to-pay thresholds for spotting days

	Main analyses – spotting days	
WTP €/ unit of effect gained	*Societal Perspective*	*Healthcare perspective*
0	0.25	0.57
31.6 (1 working hour)	0.25	0.57
126.4 (4 working hours)	0.26	0.58
252.8 (8 working hours)	0.26	0.59
	**Main analyses - QALYs**	
0	0.25	0.57
10000	0.23	0.48
20000	0.22	0.41
30000	0.21	0.35
40000	0.20	0.31
50000	0.19	0.29

WTP=willingness-to-pay; QALY=quality-adjusted life-years

For peer review only

Stegwee et al. BMC Pregnancy and Childbirth (2019) 19:85
https://doi.org/10.1186/s12884-019-2221-y

BMC Pregnancy and Childbirth

STUDY PROTOCOL Open Access

Single- versus double-layer closure of the caesarean (uterine) scar in the prevention of gynaecological symptoms in relation to niche development – the 2Close study: a multicentre randomised controlled trial

S. I. Stegwee[1†], I. P. M. Jordans[1†], L. F. van der Voet[2], M. Y. Bongers[3,19], C. J. M. de Groot[1], C. B. Lambalk[1], R. A. de Leeuw[1], W. J. K. Hehenkamp[1], P. M. van de Ven[4], J. E. Bosmans[5], E. Pajkrt[6], E. A. Bakkum[7], C. M. Radder[8], M. Hemelaar[9], W. M. van Baal[10], H. Visser[11], J. O. E. H. van Laar[3], H. A. A. M. van Vliet[12], R. J. P. Rijnders[13], M. Sueters[14], C. A. H. Janssen[15], W. Hermes[16], A. H. Feitsma[17], K. Kapiteijn[18], H. C. J. Scheepers[19], J. Langenveld[20], K. de Boer[21], S. F. P. J. Coppus[22], D. H. Schippers[23], A. L. M. Oei[24], M. Kaplan[25], D. N. M. Papatsonis[26], L. H. M. de Vleeschouwer[27], E. van Beek[28], M. N. Bekker[29], A. J. M. Huisjes[30], W. J. Meijer[31], K. L. Deurloo[32], E. M. A. Boormans[33], H. W. F. van Eijndhoven[34] and J. A. F. Huirne[1*]

Abstract

Background: Double-layer compared to single-layer closure of the uterus after a caesarean section (CS) leads to a thicker myometrial layer at the site of the CS scar, also called residual myometrium thickness (RMT). It possibly decreases the development of a niche, which is an interruption of the myometrium at the site of the uterine scar. Thin RMT and a niche are associated with gynaecological symptoms, obstetric complications in a subsequent pregnancy and delivery and possibly with subfertility.

(Continued on next page)

* Correspondence: j.huirne@vumc.nl
†S. I. Stegwee and I. P. M. Jordans contributed equally to this work.
[1]Department of Obstetrics and Gynaecology, Research institutes 'Amsterdam Cardiovascular Sciences' and 'Amsterdam Reproduction and Development', Amsterdam UMC, Vrije Universiteit Amsterdam, De Boelelaan 1117, 1081 HV Amsterdam, Netherlands
Full list of author information is available at the end of the article

© The Author(s). 2019 **Open Access** This article is distributed under the terms of the Creative Commons Attribution 4.0 International License (http://creativecommons.org/licenses/by/4.0/), which permits unrestricted use, distribution, and reproduction in any medium, provided you give appropriate credit to the original author(s) and the source, provide a link to the Creative Commons license, and indicate if changes were made. The Creative Commons Public Domain Dedication waiver (http://creativecommons.org/publicdomain/zero/1.0/) applies to the data made available in this article, unless otherwise stated.

For peer review only - http://bmjopen.bmj.com/site/about/guidelines.xhtml

(Continued from previous page)

Methods: Women undergoing a first CS regardless of the gestational age will be asked to participate in this multicentre, double blinded randomised controlled trial (RCT). They will be randomised to single-layer closure or double-layer closure of the uterine incision. Single-layer closure (control group) is performed with a continuous running, unlocked suture, with or without endometrial saving technique. Double-layer closure (intervention group) is performed with the first layer in a continuous unlocked suture including the endometrial layer and the second layer is also continuous unlocked and imbricates the first. The primary outcome is the reported number of days with postmenstrual spotting during one menstrual cycle nine months after CS. Secondary outcomes include surgical data, ultrasound evaluation at three months, menstrual pattern, dysmenorrhea, quality of life, and sexual function at nine months. Structured transvaginal ultrasound (TVUS) evaluation is performed to assess the uterine scar and if necessary saline infusion sonohysterography (SIS) or gel instillation sonohysterography (GIS) will be added to the examination. Women and ultrasound examiners will be blinded for allocation. Reproductive outcomes at three years follow-up including fertility, mode of delivery and complications in subsequent deliveries will be studied as well. Analyses will be performed by intention to treat. 2290 women have to be randomised to show a reduction of 15% in the mean number of spotting days. Additionally, a cost-effectiveness analysis will be performed from a societal perspective.

Discussion: This RCT will provide insight in the outcomes of single- compared to double-layer closure technique after CS, including postmenstrual spotting and subfertility in relation to niche development measured by ultrasound.

Trial registration: Dutch Trial Register (NTR5480). Registered 29 October 2015.

Keywords: Caesarean section, Closure techniques, Long-term outcomes, Postmenstrual spotting, Niche, Quality of life, Fertility, Reproductive outcomes,

Background

Caesarean section (CS) rates have increased from 14.5 to 27.2% in the last two decades in the Western world. [1] In 2016, 26.664 CSs were performed in the Netherlands, being 16.0% of the total number of deliveries. [2] The increasing CS rate has stimulated an interest in the potential long-term morbidity of a CS scar, such as uterine rupture or malplacentation. [3–7] Other less severe, but more prevalent long-term symptoms are gynaecological symptoms and subfertility.

Only recently, gynaecological symptoms such as painful menstruations and postmenstrual spotting have been associated with CSs. [8–10] These symptoms are considered to be related to a niche, defined as *"an indentation at the site of the caesarean scar with a depth of at least 2 mm"*, visible on transvaginal ultrasound (TVUS). [11] Two cohort studies reported a strong association between postmenstrual spotting and a niche: odds ratio (OR) 3.1; 95% confidence interval (CI) 1.5–6.3 [8] and OR 5.5; 95% CI 1.1–26.5. [10] In these studies, a niche was observed in 50 to 60% of the women after a CS, using transvaginal ultrasound. [8, 10] Spotting was correlated to niche volume and inversely correlated to the residual myometrium thickness (RMT). [8, 10]

In addition to the gynaecological symptoms, a niche may influence fertility. A recent meta-analysis reported that a CS on average reduced the probability of subsequent pregnancy with 9% (relative risk (RR) 0.91; 95% CI 0.87–0.95) in comparison to a vaginal delivery. [12] None of the included studies in this meta-analysis evaluated the relation between subsequent fertility and the presence of a niche. One of the hypotheses is that intra-uterine fluid or cervical mucus or blood accumulation in the niche are expected to hamper the penetration of sperm cells or impair embryo implantation. [13] Long-term follow-up will facilitate the evaluation of the association between uterine closure, niche development, accumulation of intra-uterine fluid and subfertility.

In the last years, various therapies have been developed and implemented to treat niche related symptoms such as menstrual disorders. [14–18] Effectiveness of both hysteroscopic [19] and laparoscopic niche resection [15] have recently been published. Because both niche related symptoms and applied therapies lead to increases in medical consultations and costs, it seems to be more efficient to prevent niche development in the first place. Uterine closure technique of the CS scar has been proposed as an independent factor for niche development. [9] However, large randomised trials evaluating the effect of uterine closuring techniques on postmenstrual spotting or other gynaecological or reproductive outcomes in relation to niche development and thin residual myometrium are lacking, as well as cost-effectiveness evaluations.

In order to shorten surgery time and in the absence of significant differences in short-term outcomes [20, 21], most Dutch gynaecologists (92%) have replaced double-layer by single-layer closure after a CS, using

For peer review only - http://bmjopen.bmj.com/site/about/guidelines.xhtml

multifilament continuous unlocked sutures. Given the higher risk on myometrium loss and thus development of a thinner residual myometrium after single-layer closure [5, 22], we hypothesise that this method introduces a higher risk on postmenstrual spotting and possibly subfertility after a CS and that it can be prevented by applying double-layer unlocked closure.

Double-layer unlocked closure is considered safe, without a clinically relevant higher risk on short-term outcomes. [5, 23, 24]. Moreover, it results in a thicker residual myometrium, especially when unlocked sutures are applied. [5, 22, 24] Dysmenorrhea was reported more frequently after single-layer closure, but this was only studied in two RCTs and not always related to ultrasound findings such as myometrial thickness or niche presence. [24] Prevalence of uterine rupture seems to be similar after single- versus double-layer closure [5, 22, 24], but has neither been related to ultrasound findings and since it has a very low incidence, statistically significant differences are difficult to find. Since long-term outcomes such as gynaecological symptoms, fertility outcomes and results of subsequent pregnancies are studied infrequently, additional evidence is needed before a preference for either technique can be indicated.

Objective

Our primary objective is to determine the effectiveness of unlocked double-layer uterine closure compared to unlocked single-layer uterine closure in the prevention of niche related gynaecological symptoms nine months after a first CS. Secondary objectives are to assess niche prevalence measured by ultrasound at three months follow-up and to study both reproductive outcomes related to a subsequent pregnancy and gynaecological symptoms at three years follow-up. Additionally we aim to study the cost-effectiveness alongside the trial.

Methods/design

Design

This multicentre randomised controlled superiority trial will be performed in the Netherlands, in hospitals that collaborate within the Dutch Consortium for Healthcare Evaluation and Research in Obstetrics and Gynaecology (NVOG Consortium 2.0, www.zorgevaluatienederland.nl). Centres that participate are district, teaching or university hospitals in the Netherlands. A list of study sites is available in Additional file 1.

Participants and eligibility criteria

All women who undergo a first CS, planned or unplanned, will be asked to participate in the study. Other inclusion criteria are: sufficient command of the Dutch or English language, age ≥ 18 years and written informed consent. To prevent confounding effects on niche development during the study, we will exclude women with a previous CS. Other exclusion criteria are: inadequate possibility for counselling (e.g. indication for emergency CS without being informed about the study previously, women in severe pain without adequate therapy), previous major uterine surgery (e.g. laparoscopic or laparotomic fibroid resection, septum resection), women with known causes of menstrual disorders (e.g. cervical dysplasia, communicating hydrosalpinx, uterine anomaly or endocrine disorders disturbing ovulation), placenta inor percreta during the current pregnancy or ≥ three foetuses during the current pregnancy.

Recruitment and randomisation

Eligible women will be asked by a gynaecologist, resident, clinical midwife or research nurse to participate in the trial when they undergo a planned CS. Eligible women who are planned to undergo a vaginal delivery will also be informed about this study during pregnancy in case they need an unplanned CS. Furthermore, women during induced labour and women receiving adequate therapy for pain during labour, will be asked to participate in case a CS is needed during labour for any indication.

When the decision of a CS is made and all selection criteria are met, women will be randomly allocated to single-layer (control group) or double-layer (intervention group) closure (1:1) (see Fig. 1). Randomisation will be performed using a web-based application ALEA 2.2 which displays a computer-generated random number, managed by the Clinical Research Unit of the Amsterdam UMC - location AMC. We will use a permuted block-design, stratified for recruiting centres and for planned or unplanned CS. All women that decline to participate will be registered anonymously in order to record the number and reason for refusal. Subjects who withdraw from this study will not be replaced.

Gynaecologists, residents, clinical midwives or research nurses enrol participants and assign them to the intervention. The CS will be performed by either a gynaecologist, a resident supervised by a gynaecologist or by a resident that is authorised to perform CSs without supervision. Participants and sonographers will be blinded for the closure technique. If operative reintervention after CS is needed and the gynaecologist that performs the reintervention needs to know the closure technique that the participant was assigned to, unblinding is possible through the logistic trial coordinator. We expect this situation to occur very infrequently.

Intervention (double-layer closure)

In both study arms, women will undergo a CS following a standard way with respect to mode of uterotomy,

For peer review only

correct approximations of the cutting edges and non-closure of the peritoneum. In the intervention arm, double-layer closure of the uterus will be performed using unlocked multifilament continuous running sutures for both layers and the endometrial layer will be included in the first layer (see Fig. 2). The second layer is a continuous running suture that imbricates the first layer. Since this is not the standard method for uterine closure in the Netherlands, a short online instruction film will be shown to all participating centres and surgeons prior to participation (see Additional file 2). Surgical outcomes will be registered after the procedure in the electronic case report form (eCRF).

Control group (single-layer closure)

The control group will receive usual closure technique of the uterus: a single-layer closure using unlocked continuous running multifilament sutures. The currently available evidence is inconclusive with respect to endometrial saving technique or not. Therefore, we decided that in our study surgeons are free to choose to close either full thickness (including the endometrium) or split

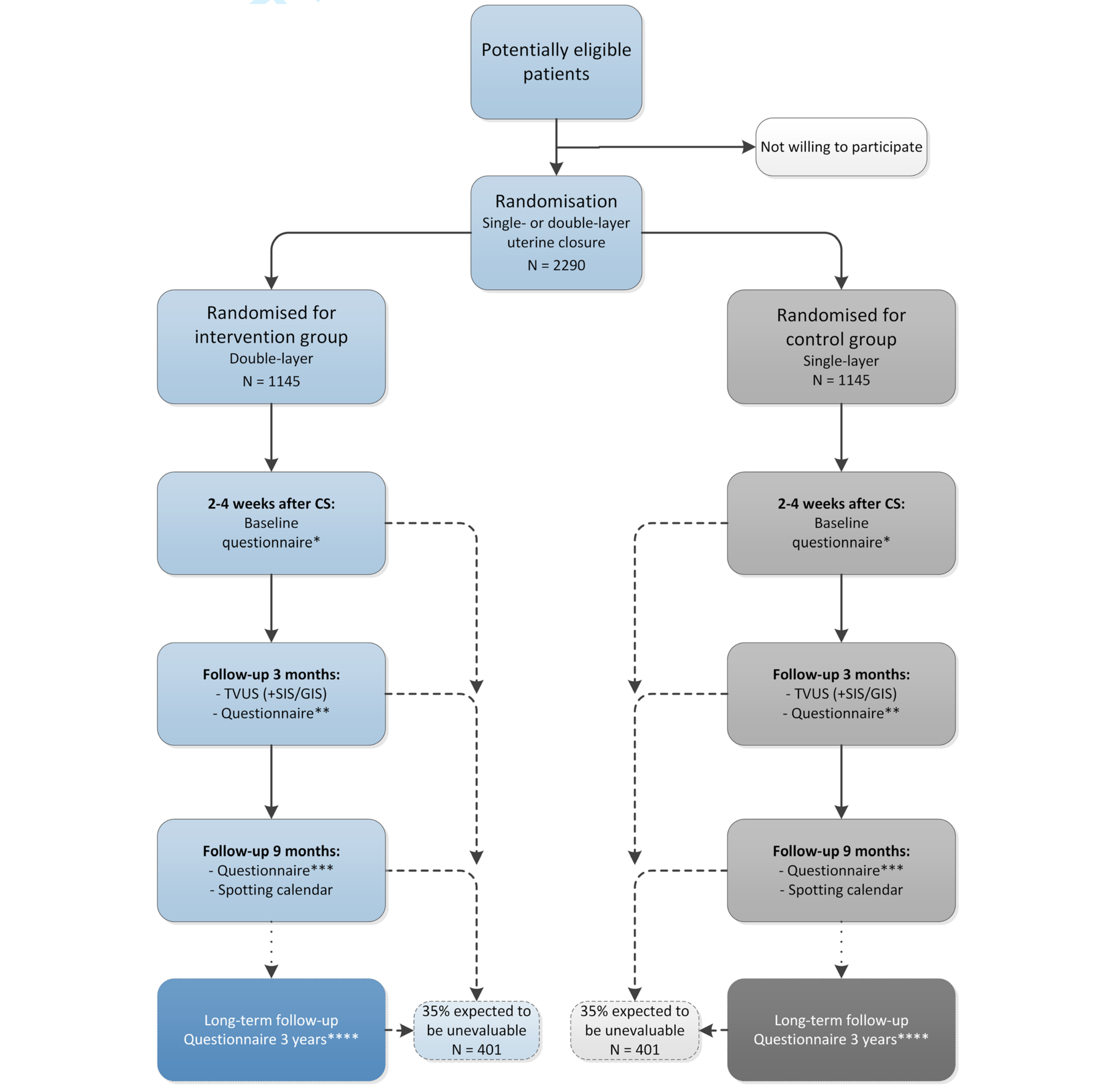

Fig. 1 Flowchart of the 2Close study. * = baseline questions, EQ-5D-5L. ** = symptom questionnaire, EQ-5D-5L, SF36, PROMIS SF8a, iMCQ, iPCQ. *** = symptom questionnaire, FSFI, EQ-5D-5L, SF36, iMCQ, iPCQ. **** = symptom questionnaire, fertility questionnaire, FSFI, EQ-5D-5L, SF36, PROMIS SF8a

thickness (excluding the endometrium) in the control group. The applied method, including endometrial saving technique or not, will be registered.

Niche evaluation

The care after CS will be according to the normal local protocol with the regular outpatient visit that is normally executed six weeks after the CS. This routine visit may be postponed to three months after the CS to enable an ultrasound evaluation to identify the existence of a niche, but participating centres may decide whether they want visits at six weeks (routine follow-up) and at three months (ultrasound follow-up) or only one visit after three months combining the regular control and the ultrasound follow-up. The ultrasound evaluation is standardised as proposed by Jordans et al. [11] (see Fig. 3). Based on this standardisation, we created an obligatory e-learning for all ultrasound performers to let all ultrasounds be performed in a uniform manner. To increase consistency and to improve the learning curve, we will evaluate a sample of ultrasounds in each centre based on recorded pictures and provide feedback to the examiners. Since it is known that a niche can be missed during TVUS only [8, 10, 25] we will additionally perform a saline infusion sonohysterography (SIS) or gel instillation sonography (GIS) in case no niche is observed during the normal TVUS or if the ultrasound is inconclusive. It would be optimal to have a contrast enhanced ultrasound in all women when the uterine cavity or niche are not naturally filled with fluid, but we have chosen for this approach to prevent unnecessary burden for the participants and to reduce costs.

Outcome measures

Primary outcome measure

The primary outcome is the number of days of postmenstrual spotting during one cycle at nine months after CS. We defined postmenstrual spotting as brownish discharge for more than two days at the end of the menstruation, with a total duration (menstruation and spotting) of more than seven days, or intermenstrual blood loss that starts after the end of the menstruation. [8] The number of days of postmenstrual spotting will be counted as follows: days with brownish discharge (> two days) when the total duration of menstruation and spotting exceeds seven days + number of days with

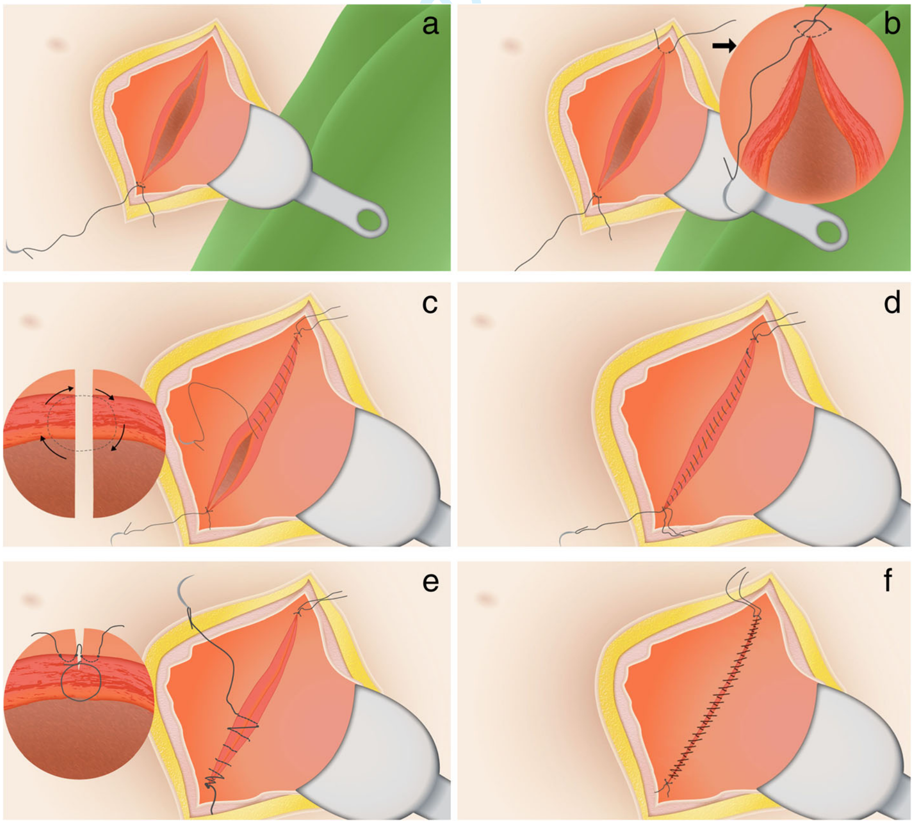

Fig. 2 Double-layer uterine closure technique. **a**. Step 1: lateral suture; **b**. Step 2: lateral suture on the other side; **c**. Step 3: First layer: full thickness, continuous, including large part of myometrium, including the endometrial layer; **d**. Step 4: End of this first layer; **e**. Step 5: Second layer: superficial continuous layer of serosal tissue, imbricating the first layer; **f**. Step 6: First and second layer should be closely connected

intermenstrual blood loss. Amenorrhoeic women, due to lactation, medication or other diseases, will not be evaluable for the primary outcome and will be left out of this analysis.

Secondary outcome measures at short-term

- Perioperative outcomes including blood loss, operative time, additional haemostatic sutures and complications.
- Menstruation characteristics, dysmenorrhea (visual analogue scale (VAS)), Quality of Life (QOL) using Short-Form-36 [26] and EQ-5D-5L [27, 28], societal reintegration (PROMIS Short-Form-8a [29]), sexual function using the Female Sexual Function Index (FSFI [30]), applied medical and/or surgical therapy because of gynaecological symptoms, all obtained through digital questionnaires, will be assessed at three and nine months follow-up.
- Ultrasound evaluation will be performed at three months follow-up using TVUS, in which RMT, adjacent myometrium thickness (AMT), presence of a niche (depth of ≥2mm), length, depth and width of the niche, presence of large niches (RMT < 50% of AMT, RMT <3mm) and niche volume will be measured.

Cost-effectiveness outcomes

Costs will be measured using adapted versions of the iMTA Productivity Cost Questionnaire (iPCQ [31]) and iMTA Medical Consumption Questionnaire (iMCQ [32]) from a health care and societal perspective at nine months of follow-up.

Secondary outcome measures at long-term

- Menstruation characteristics, pain, sexual functioning, QOL and social reintegration will be evaluated at three years follow-up.
- Reproductive outcomes at three years follow-up: % of women desiring to conceive, % of women that conceived including time to conceive, % of women with an ongoing pregnancy, the need for fertility treatment and pregnancy outcomes such as mode of delivery or complications will be determined.

Long-term outcomes will be presented in a separate article.

Data collection and data management

Intraoperative data

Immediately after the CS we will register relevant items regarding the delivery and CS in an eCRF, in which confidentiality and anonymity are ensured and audit trails are accessible. These items include: reason for planned or unplanned CS, emergency CS or not, whether women experienced contractions, dilatation, performed method for uterine closure, endometrial saving technique applied, used suturing material, extra haemostatic sutures, operative time, blood loss and complications.

Collection of baseline characteristics and patient reported outcomes

Baseline characteristics will be collected through a digital questionnaire at 2–4 weeks after caesarean section, sent to the e-mail address of participants. Since we will also include unplanned CSs, we decided that it is not possible for all participants to answer questions regarding baseline characteristics before the operation. Baseline parameters include maternal age, body mass index, social economic status, smoking habit, medical and obstetric history, gestational age and previous vaginal deliveries, all reported by the participant. We expect that the impact of niche related symptoms such as postmenstrual spotting on daily activities and sexual behaviour may be influenced by ethnic background and religion, therefore we will also register these characteristics. At three months, nine months and three years follow-up, again digital questionnaires will be sent to participants to assess the primary and secondary outcomes (see Fig. 1). At nine months, we ask participants record their exact menstrual and spotting pattern, if any, in an adjusted menstruation score chart. [33] Reminders for all questionnaires will be sent every two weeks, with a maximum of three times. When no response is given after the reminders, research nurses from participating centres will be asked to call the participant.

Data niche evaluation

Results of the TVUS and GIS or SIS, performed three months after CS, will be registered. Women will not receive information regarding the presence of a niche, since it has no clinical consequences so shortly after CS and this may influence the answers given in the questionnaires. Other important abnormalities visualised by ultrasound will be reported as usual.

Statistical issues

Sample size calculation

We use a superiority design since we expect double-layer closure to be favourable. Literature for making reliable estimations on postmenstrual spotting in relation to niches is scarce. We have used baseline data from the HysNiche [19] and LapNiche [15] study. We

For peer review only

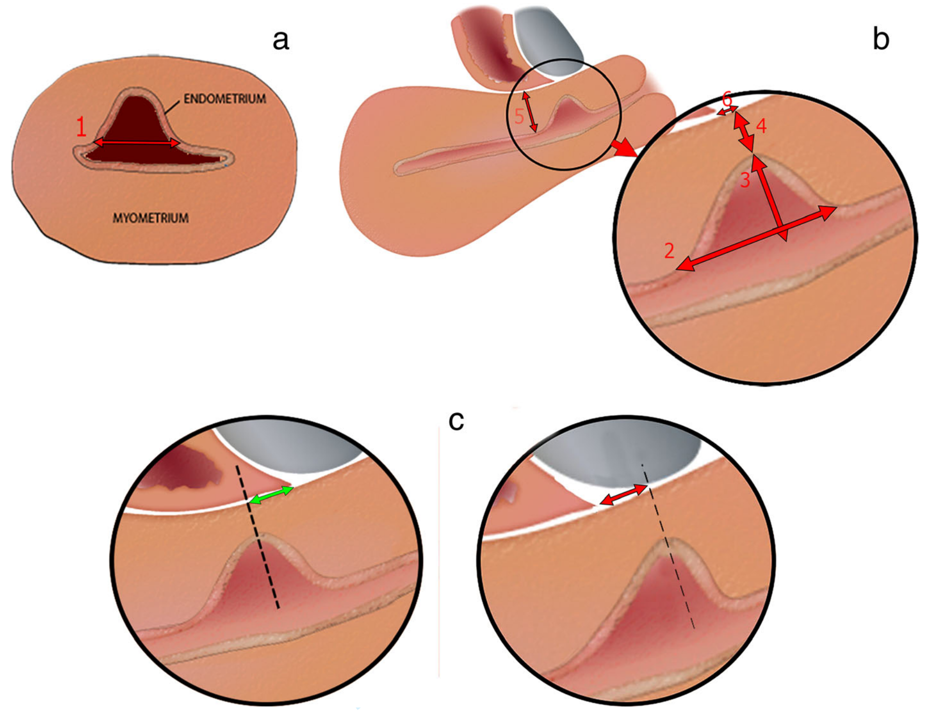

Fig. 3 Standardised transvaginal ultrasound evaluation of a niche in the non-pregnant uterus. **a**. Measured in the transversal plane: niche width (1); **b**. Measured in the sagittal plane: niche length (2), niche depth (from cervical canal until apex of the niche) (3), residual myometrium thickness (from deepest part of the niche until the serosa) (4), adjacent myometrium thickness (myometrium thickness close to base of the niche) (5), distance from apex of the niche to vesico-vaginal fold (see **c**) (6); **c**. Measuring the niche relatively to the vesico-vaginal fold in the sagittal plane: positive value (green arrow, in mm) or negative value (red arrow, in mm)

estimate the mean number of spotting days to be 3.5 days/month in the total group. We consider a 15% reduction in the mean number of spotting days clinically relevant, which is 0.5 day/month reduction. Assuming a standard deviation (SD) of 3.4 and a two-sided significance level of 5%, a total of 1488 women need to be included to achieve a power of 80%. Increasing the sample size to take into account 35% of women unevaluable (due to drop-out, non-response or amenorrhoea) for the primary outcome, 2290 women need to be included.

Data-analysis

Data-analysis will be performed according to the intention to treat principle and additional per protocol analyses will be performed. A test will be considered statistically significant when the two-sided test shows a p-value < 0.05. Baseline characteristics will be presented using percentages, means with SD and 95% CI or medians with interquartile ranges (IQR), where appropriate.

The primary outcome, number of days of postmenstrual spotting, will be presented for both groups as mean with SD or median with IQR, and presented in a Box-Whisker graph to show the distribution. Differences in primary outcome between the groups will be tested using the independent t-test in case of normal distribution (possible after transformation of the outcome) or Mann-Whitney U test. An adjusted analysis will be performed using linear regression analysis in which we adjust for factors on which randomisation was stratified and for baseline factors on which relevant differences are observed despite randomisation.

Dichotomous secondary outcomes will be presented as percentages and RR with corresponding 95% CI. *P*-values will be calculated using the chi-square test or, if the expected count for at least one cell is below 5, using the Fisher exact test. Normally distributed continuous variables will be presented as means with SD, and differences between the groups will be calculated with an independent t-test. Non-normally distributed continuous variables will be presented as medians with IQR and differences between the groups will be calculated with Mann-Whitney U test. The questionnaires will be analysed using the appropriate algorithms and usual presentation methods (FSFI, EQ-5D-5L, SF36, PROMIS SF8a, iMCQ, iPCQ).

Comparison of primary outcome between women receiving single- and double-layer closure will be done as secondary analyses within each of the following subgroups separately:

For peer review only - http://bmjopen.bmj.com/site/about/guidelines.xhtml

1. Planned (without labour) or unplanned (in labour) CS
2. Emergency CS or not
3. Preterm (< 37 weeks gestational age) or term (≥ 37 week gestational age) CS
4. Presence (> 3cm) or absence (≤ 3cm) of dilatation
5. Placenta praevia or not
6. Presence or absence of specific maternal morbidity (e.g. diabetes, pre-eclampsia, haemolysis/elevated liver enzymes/low platelet count (HELLP) syndrome, immunodeficient women)
7. Singleton versus twin pregnancy
8. Natural cycle or hormonally induced withdrawal bleeding

Within the single-layer group (control group) we will compare the primary outcome between women in whom endometrial saving technique (split thickness) was applied and women in whom an endometrial saving technique was not applied (full thickness).

Economic evaluation

The economic evaluation will be performed alongside the RCT from a societal perspective. Both a cost-effectiveness and cost-utility analysis will be performed with a time horizon of nine months to relate the difference in societal and healthcare costs between double-layer and single-layer unlocked uterine closure during a CS to the difference in clinical effects. Healthcare costs include costs of primary and secondary care, complementary care and home care. Costs in other sectors include presence and absence from paid and unpaid work. The friction cost approach will be used to estimate indirect costs. For the valuation of health care utilization standard prices published in the Dutch Costing guidelines will be used. [34] Medication use will be valued using prices of the Royal Dutch Society for Pharmacy.

Societal costs will be related to the following effect measures in the economic evaluation: days with postmenstrual spotting and quality-adjusted life-years (QALYs) based on the Dutch tariff for the EuroQol (EQ-5D-5L). [27, 28, 35]

We hypothesise that double-layer uterine closure will reduce postmenstrual spotting and related consultations for gynaecological or fertility related problems and applied therapies, and as a consequence that it will be cost-effective in comparison with single-layer uterine closure.

The analysis will be done according to the intention to treat principle. Missing costs and effect data will be imputed using multiple imputation. Incremental cost-effectiveness ratios (ICERs) will be calculated by dividing the difference in mean total costs between the treatment groups by the difference in mean effects. Bootstrapping with 5000 replications will be used to estimate 95% CI around cost differences and the uncertainty surrounding the ICERs. Uncertainty surrounding the ICERs will be graphically presented on cost-effectiveness planes. Cost-effectiveness acceptability curves showing the probability that double-layer uterine closure is cost-effective in comparison with single-layer uterine closure for a range of different ceiling ratios will also be estimated. Adjustment for confounders and effect modifiers will be done if necessary. [36]

Interim analysis and safety monitoring

Because of the type of intervention, the Medical Ethics Committee (MEC) determined that the risk for participation is negligible. Therefore, we do not have a Data Safety Monitoring Committee. No interim analysis is planned.

All serious adverse events (SAEs) will be reported to the MEC by line listing yearly. Life threatening SAEs or an event that leads to death will be reported to the MEC immediately. All SAEs will be followed until they have abated, until a stable situation has been reached or the patient was discharged. We do not expect to terminate the study prematurely given the low risk of adverse events.

Confidentiality and data security

All participating centres receive a login name and password to gain access to ALEA 2.2, the web-secured randomisation database. Randomisation is performed pseudo-anonymously with only the initials and year of birth of the participants. Linking personal data to the study number can only be performed in the local participating centres or by the trial coordinator (SS). Written informed consent forms are stored in every centre in a lockable room. All forms and data will be archived for 15 years in the participating centres.

Discussion

In the last years, studies examining complications of CSs are increasing, including the development of niches or thin residual myometrium at the site of the previous CS and related symptoms. Both RMT and the presence of a niche have been associated with postmenstrual spotting. [8, 10] Double-layer unlocked closure has been shown to result in a thicker residual myometrium and as a consequence can possibly lead to a decrease of niche development after a CS compared to single-layer closure. [5, 22, 24, 37] However, the long-term clinical outcomes in terms of postmenstrual spotting or subfertility have not been studied previously or have not been related to ultrasound findings. We hypothesise that niche related postmenstrual spotting and fertility problems will reduce together with decrease in niche prevalence, in which

For peer review only

identification of the best uterine closure technique regarding RMT and niche development will be of great significance.

Strengths and limitations

The design of this study is one of the strengths; this is the first large RCT that will evaluate the effectiveness of double-layer uterine closure compared to single-layer uterine closure after CS regarding niche related gynaecological symptoms and reproductive outcomes with a long-term follow-up. The study is adequately powered. Randomisation is performed by using a web based randomisation program. Furthermore, all participants and examiners are blinded which reduces the chance for bias regarding reported symptoms and ultrasound findings. An additional strength is the uniform manner in which we try to perform double-layer closure and ultrasound evaluation, instructed by mandatory online instruction film and e-learning, respectively. Moreover, the 2Close study will compare the cost-effectiveness of both techniques which has never been done before. As we expect that double-layer closure will reduce the incidence of niche development and as a consequence that it could possibly reduce the gynaecological symptoms including postmenstrual spotting after CS, we assume double-layer closure to be more cost-effective. Also, we expect that double-layer closure will improve the chances of conceiving after CS and lower costs in fertility treatment.

We also expect some limitations. Baseline characteristics will be collected through questionnaires that are filled in by women in the first month after CS, which might lead to recall bias regarding medical history, complications during pregnancy and labour, and other baseline measurements. We decided to lower the administrative load for participating hospitals by obtaining these characteristics through the participants. Furthermore, there is no validated questionnaire available yet for postmenstrual spotting; therefore, the questionnaires that are used in the 2Close study are not adjusted or validated for these symptoms. Moreover, the surgical techniques performed during the CS in this study are standardised in both study arms except for saving the endometrium in the control group. There is no conclusive evidence whether or not to save the endometrium in the suture according to its influence on niche development. Therefore, we chose to leave this decision with the surgeons. There may possibly be a difference in the incidence of niche development between the participants receiving single-layer split thickness or full thickness closure, also when compared to the incidence of niche development in the double-layer group. This will be further examined in a subgroup analysis.

To prevent bias regarding niche evaluation three months after CS, all ultrasonographic examiners are trained by an online learning program and a sample of ultrasounds will be evaluated. The learning module is based on the results of a Delphi procedure among international niche experts. [11] Although the niche examiners in the 2Close are trained by a standardised method, experience in measuring niches and as a consequence differences in niche measurement may occur among examiners.

Potential impact and implications

This study will gain insight in the most optimal uterine closure technique after CS which is relevant for women and gynaecologists, since we will focus on long-term gynaecological symptoms and reproductive outcomes in relation to changes of the lower uterine segment after CS and in particular niche development. Since many studies have already shown that RMT and niches are related to several symptoms and therapies for niche resection are being developed, we think it is necessary to provide evidence for the development of preventive strategies regarding niche related symptoms. It is important to realise that the best way to prevent a niche and its related symptoms, is to not perform a CS. But since it is often inevitable to perform a CS, care takers should perform it in the most optimal way.

After the results of this study become available, the most optimal and cost-effective technique can be implemented in order to reduce symptoms and problems in a subsequent pregnancy. This will not be difficult, since the technique is easy to learn and many gynaecologists and residents are familiar with it after the trial. Especially for a scheduled CS, women should be informed about the risk to develop a niche and the risk that it might cause symptoms or complications later in life.

Additional files

Additional file 1: Affiliations of all 32 participating hospitals in the Netherlands that granted approval. The board of the hospitals granted approval to participate and to start recruiting patients. (DOCX 15 kb)

Additional file 2: Text of the online standardised instruction film for double-layer closure of the uterotomy. The spoken text in the online instruction film, which shows a standardised way to perform double-layer closure of the uterotomy, has been translated into English. (DOCX 15 kb)

Abbreviations

AMT: Adjacent myometrium thickness; BROK: Basic course Regulations and Organisation for Clinical researchers; CI: Confidence interval; eCRF: electronic case report form; CS: Caesarean section; GCP: Good clinical practice; GIS: Gel instillation sonohysterography; ICER: Incremental cost-effectiveness ratio; iMCQ: iMTA Medical Consumption Questionnaire; iMTA: Institute for Medical Technology Assessment; iPCQ: iMTA Productivity Cost Questionnaire; IQR: Interquartile range; MEC: Medical ethics committee; OR: Odds ratio; QALY: Quality adjusted life years; QOL: Quality of life; RCT: Randomised controlled trial; RMT: Residual myometrium thickness; RR: Relative risk; SAE: Serious adverse event; SD: Standard deviation; SIS: Saline infusion sonohysterography; TVUS: Transvaginal ultrasound; VAS: Visual analogue scale

For peer review only

Acknowledgements
None.

Funding
This study group received a grant from The Netherlands Organisation for Health Research and Development (ZonMw, project number: 843002605, grant number: 80–84300–98-62021), a Dutch organization that supports research in health and health innovation. The funder peer-reviewed the study design and will receive an annual progress report of the trial, but has no further role in the collection, analysis and interpretation of data, writing of the manuscript or decision to submit the report for publication.

Availability of data and materials
Data entry and secure storage is performed with data management system OpenClinica (OpenClinica LLC and collaborators, facilitated by TraIT (Translational Research IT infrastructure) and hosted by Vancis, which meets the ISO 27001 standard for information security management, version: 3.6). Data control during the trial is possible. The trial will be monitored on-site by a quality advisor of the Dutch Consortium for Studies in Women's Health and Reproduction. The quality advisor will also perform data verification at the end of the trial. The NVOG Consortium stores all data from clinical trials centrally so data will not become publicly available but will be available upon reasonable request to the corresponding author and with permission of the NVOG Consortium. Trial data will be accessible for verification or other research purposes, after an embargo period of 12 months in order to enable publication of the results by the project group.

Authors' contributions
JH, IJ, LvdV, MB, CdG, CL, RdL and WH made substantial contributions to the conception and design of the study. SS is responsible for the overall logistical aspects of the trial and general acquisition of data. SS and IJ drafted the manuscript. PV and JB provided statistical support regarding future analyses. EP, EB1, CR, MH, WvB, HV, JvL, HvV, RR, MS, CJ, WH, AF, KK, HS, JL, KdB, SC, DS, AO, MK, DP, LdV, EvB, MB, AH, WM, KD, EB2 and HvE made substantial contributions to acquisition of data. All authors critically revised the manuscript, approved the final version of the manuscript and agreed to be accountable for all aspects of the manuscript ensuring that questions related to the accuracy or integrity of the work are appropriately investigated and resolved.

Ethics approval and consent to participate
This study is approved by the ethics committee of Amsterdam UMC - location VUmc (reference number: 2015.462) and by the boards of all participating hospitals (Additional file 1). Protocol amendments will be communicated to participating hospitals, after approval of the central MEC of Amsterdam UMC – location VUmc. This trial is registered in the Dutch Trial Register (NTR5480, www.trialregister.nl). Counselling of eligible women will be done according to the 'good clinical practice' (GCP) guidelines or doctors certified according to Basic course Regulations and Organisation for Clinical researchers (BROK). After reading the patient information form and counselling by GCP- or BROK-certified personnel, women will be asked for written consent. This informed consent form will be obtained before participation in the clinical trial.
The research team declares that the SPIRIT guidelines were followed. All recommended items of the SPIRIT 2013 checklist were addressed in this clinical trial protocol.
The trial results will be communicated after the final inclusion and follow-up through publication in a peer-reviewed international journal and in an international congress. Participants will be informed through e-mail and the study website.

Consent for publication
Not applicable.

Competing interests
All authors declare that they have no competing interests.

Publisher's Note
Springer Nature remains neutral with regard to jurisdictional claims in published maps and institutional affiliations.

Author details
[1]Department of Obstetrics and Gynaecology, Research institutes 'Amsterdam Cardiovascular Sciences' and 'Amsterdam Reproduction and Development', Amsterdam UMC, Vrije Universiteit Amsterdam, De Boelelaan 1117, 1081 HV Amsterdam, Netherlands. [2]Department of Obstetrics and Gynaecology, Deventer Hospital, Nico Bolkesteinlaan 75, 7416 SE Deventer, the Netherlands. [3]Department of Obstetrics and Gynaecology, Máxima Medical Centre, De Run 4600, 5504 DB Veldhoven, the Netherlands. [4]Department of Epidemiology and Biostatistics, Vrije Universiteit Amsterdam, De Boelelaan 1105, 1081 HV Amsterdam, the Netherlands. [5]Department of Health sciences, Faculty of Science, Research institute 'Amsterdam Public Health', Vrije Universiteit Amsterdam, De Boelelaan 1085, 1081 HV Amsterdam, the Netherlands. [6]Department of Obstetrics and Gynaecology, Amsterdam UMC, University of Amsterdam, Meibergdreef 9, 1105 AZ Amsterdam, the Netherlands. [7]Department of Obstetrics and Gynaecology, OLVG-oost, Oosterpark 9, 1091 AC Amsterdam, the Netherlands. [8]Department of Obstetrics and Gynaecology, OLVG-west, Jan Tooropstraat 164, 1061 AE Amsterdam, the Netherlands. [9]Department of Obstetrics and Gynaecology, Westfriesgasthuis, Maelsonstraat 3, 1624 NP Hoorn, the Netherlands. [10]Department of Obstetrics and Gynaecology, Flevo hospital, Hospitaalweg 1, 1315 RA Almere, the Netherlands. [11]Department of Obstetrics and Gynaecology, Tergooi hospital, Rijksstraatweg 1, 1261 AN Blaricum, the Netherlands. [12]Department of Obstetrics and Gynaecology, Catharina hospital, Michelangelolaan 2, 5623 EJ Eindhoven, the Netherlands. [13]Department of Obstetrics and Gynaecology, Jeroen Bosch hospital, Henri Dunantstraat 1, 5223 GZ 's-Hertogenbosch, the Netherlands. [14]Department of Obstetrics and Gynaecology, Leiden University Medical Centre, Albinusdreef 2, 2333 ZA Leiden, the Netherlands. [15]Department of Obstetrics and Gynaecology, Groene Hart hospital, Bleulandweg 10, 2803 HH Gouda, the Netherlands. [16]Department of Obstetrics and Gynaecology, Haaglanden Medical Centre – Westeinde hospital, Lijnbaan 32, 2512 VA Den Haag, the Netherlands. [17]Department of Obstetrics and Gynaecology, Haga hospital, Els-Borst-Eilersplein 275, 2545 AA Den Haag, the Netherlands. [18]Department of Obstetrics and Gynaecology, Reinier de Graaf hospital, Reinier de Graafweg 5, 2625 AD Delft, the Netherlands. [19]Department of Obstetrics and Gynaecology, Research school 'GROW', Maastricht University Medical Centre, P. Debyelaan 25, 6229 HX Maastricht, the Netherlands. [20]Department of Obstetrics and Gynaecology, Zuyderland Medical Centre, Henri Dunantstraat 5, 6419 PC Heerlen, the Netherlands. [21]Department of Obstetrics and Gynaecology, Rijnstate hospital, Wagnerlaan 55, 6815 AD Arnhem, the Netherlands. [22]Department of Obstetrics and Gynaecology, Radboud University Nijmegen Medical Centre, Geert Grooteplein Zuid 10, 6525 GA Nijmegen, the Netherlands. [23]Department of Obstetrics and Gynaecology, Canisius-Wilhelmina hospital, Weg door Jonkerbos 100, 6532 SZ Nijmegen, the Netherlands. [24]Department of Obstetrics and Gynaecology, Bernhoven hospital, Nistelrodeseweg 10, 5406 PT Uden, the Netherlands. [25]Department of Obstetrics and Gynaecology, Röpcke-Zweers hospital, Jan Weitkamplaan 4a, 7772 SE Hardenberg, the Netherlands. [26]Department of Obstetrics and Gynaecology, Amphia hospital, Langendijk 75, 4819 EV Breda, the Netherlands. [27]Department of Obstetrics and Gynaecology, Sint Franciscus hospital, Kleiweg 500, 3045 PM Rotterdam, the Netherlands. [28]Department of Obstetrics and Gynaecology, Sint Antonius hospital, Koekoekslaan 1, 3435 CM Nieuwegein, the Netherlands. [29]Department of Obstetrics and Gynaecology, Birth Centre Wilhelmina Children hospital/ University Medical Centre Utrecht, Lundlaan 6, 3584 EA Utrecht, the Netherlands. [30]Department of Obstetrics and Gynaecology, Gelre hospital – location Apeldoorn, Albert Schweitzerlaan 31, 7334 DZ Apeldoorn, the Netherlands. [31]Department of Obstetrics and Gynaecology, Gelre hospital – location Zutphen, Den Elterweg 77, 7207 AE Zutphen, the Netherlands. [32]Department of Obstetrics and Gynaecology, Diakonessenhuis, Bosboomstraat 1, 3582 KE Utrecht, the Netherlands. [33]Department of Obstetrics and Gynaecology, Meander Medical Centre, Maatweg 3, 3813 TZ Amersfoort, the Netherlands. [34]Department of Obstetrics and Gynaecology, Isala clinics, Dokter van Heesweg 2, 8025 AB Zwolle, the Netherlands.

Received: 20 February 2018 Accepted: 12 February 2019
Published online: 04 March 2019

References

1. World Health Organization Human Reproduction Programme. WHO statement on caesarean section rates. Sex Reprod Health. 2015;02:1–8 (WHO/RHR/15.02).
2. Perined: Perinatale Zorg in Nederland 2016. In Utrecht: Perined; 2018.

For peer review only - http://bmjopen.bmj.com/site/about/guidelines.xhtml

3. Naji O, Wynants L, Smith A, Abdallah Y, Saso S, Stalder C, Van Huffel S, Ghaem-Maghami S, Van Calster B, Timmerman D, et al. Does the presence of a caesarean section scar affect implantation site and early pregnancy outcome in women attending an early pregnancy assessment unit? Hum Reprod. 2013;28(6):1489–96.
4. Naji O, Wynants L, Smith A, Abdallah Y, Stalder C, Sayasneh A, McIndoe A, Ghaem-Maghami S, Van Huffel S, Van Calster B, et al. Predicting successful vaginal birth after cesarean section using a model based on cesarean scar features examined by transvaginal sonography. Ultrasound Obstet Gynecol. 2013;41(6):672–8.
5. Roberge S, Demers S, Berghella V, Chaillet N, Moore L, Bujold E. Impact of single- vs double-layer closure on adverse outcomes and uterine scar defect: a systematic review and metaanalysis. Am J Obstet Gynecol. 2014; 211(5):453–60.
6. Hesselman S, Hogberg U, Ekholm-Selling K, Rassjo EB, Jonsson M. The risk of uterine rupture is not increased with single- compared with double-layer closure: a Swedish cohort study. BJOG. 2015;122(11):1535–41.
7. Clark EA, Silver RM. Long-term maternal morbidity associated with repeat cesarean delivery. Am J Obstet Gynecol. 2011;205(6 Suppl):S2–10.
8. Bij de Vaate AJ, Brolmann HA, van der Voet LF, van der Slikke JW, Veersema S, Huirne JA. Ultrasound evaluation of the cesarean scar: relation between a niche and postmenstrual spotting. Ultrasound Obstet Gynecol. 2011;37(1):93–9.
9. Bij de Vaate AJ, van der Voet LF, Naji O, Witmer M, Veersema S, Brolmann HA, Bourne T, Huirne JA. Prevalence, potential risk factors for development and symptoms related to the presence of uterine niches following cesarean section: systematic review. Ultrasound Obstet Gynecol. 2014;43(4):372–82.
10. van der Voet LF, Bij de Vaate AM, Veersema S, Brolmann HA, Huirne JA. Long-term complications of caesarean section. The niche in the scar: a prospective cohort study on niche prevalence and its relation to abnormal uterine bleeding. BJOG. 2014;121(2):236–44.
11. Jordans IPM, de Leeuw RA, Stegwee SI, Amso NN, Barri-Soldevila PN, van den Bosch T, Bourne T, Brolmann HAM, Donnez O, Dueholm M, et al. Sonographic examination of uterine niche in non-pregnant women: a modified Delphi procedure. Ultrasound Obstet Gynecol. 2019;53(1):107–15.
12. Gurol-Urganci I, Bou-Antoun S, Lim CP, Cromwell DA, Mahmood TA, Templeton A, van der Meulen JH. Impact of caesarean section on subsequent fertility: a systematic review and meta-analysis. Hum Reprod. 2013;28(7):1943–52.
13. Vervoort AJ, Uittenbogaard LB, Hehenkamp WJ, Brolmann HA, Mol BW, Huirne JA. Why do niches develop in caesarean uterine scars? Hypotheses on the aetiology of niche development. Hum Reprod. 2015;30(12):2695–702.
14. van der Voet LF, Vervoort AJ, Veersema S, Bij de Vaate AJ, Brolmann HA, Huirne JA. Minimally invasive therapy for gynaecological symptoms related to a niche in the caesarean scar: a systematic review. BJOG. 2014;121(2): 145–56.
15. Vervoort A, Vissers J, Hehenkamp WJK, Brolmann HAM, Huirne JAF. The effect of laparoscopic resection of large niches in the uterine caesarean scar on symptoms, ultrasound findings and quality of life: a prospective cohort study. BJOG. 2018;125(3):317–25.
16. Vervoort AJ, Van der Voet LF, Witmer M, Thurkow AL, Radder CM, van Kesteren PJ, Quartero HW, Kuchenbecker WK, Bongers MY, Geomini PM, et al. The HysNiche trial: hysteroscopic resection of uterine caesarean scar defect (niche) in patients with abnormal bleeding, a randomised controlled trial. BMC Womens Health. 2015;15:103.
17. Nikkels C, Vervoort A, Mol BW, Hehenkamp WJK, Huirne JAF, Brolmann HAM. IDEAL framework in surgical innovation applied on laparoscopic niche repair. Eur J Obstet Gynecol Reprod Biol. 2017;215:247–53.
18. Huirne JAF, Vervoort A, Leeuw R, Brolmann HAM, Hehenkamp WJK. Technical aspects of the laparoscopic niche resection, a step-by-step tutorial. Eur J Obstet Gynecol Reprod Biol. 2017;219:106–12.
19. Vervoort A, van der Voet LF, Hehenkamp W, Thurkow AL, van Kesteren P, Quartero H, Kuchenbecker W, Bongers M, Geomini P, de Vleeschouwer L, et al. Hysteroscopic resection of a uterine caesarean scar defect (niche) in women with postmenstrual spotting: a randomised controlled trial. BJOG. 2018;125(3): 326–34.
20. Abalos E, Addo V, Brocklehurst P, El Sheikh M, Farrell B, Gray S, Hardy P, Juszczak E, Mathews JE, Masood SN, et al. Caesarean section surgical techniques (CORONIS): a fractional, factorial, unmasked, randomised controlled trial. Lancet. 2013;382(9888):234–48.
21. The CAESAR study collaborative group. Caesarean section surgical techniques: a randomised factorial trial (CAESAR). BJOG. 2010;117(11):1366–76.
22. Di Spiezio Sardo A, Saccone G, McCurdy R, Bujold E, Bifulco G, Berghella V. Risk of cesarean scar defect in single- versus double-layer uterine closure: a systematic review and meta-analysis of randomized controlled trials. Ultrasound Obstet Gynecol. 2017;50:578–83.
23. Dodd JM, Anderson ER, Gates S, Grivell RM. Surgical techniques for uterine incision and uterine closure at the time of caesarean section. Cochrane Database Syst Rev. 2014;7:CD004732.
24. Stegwee SI, Jordans I, van der Voet LF, van de Ven PM, Ket J, Lambalk CB, de Groot C, Hehenkamp W, Huirne J. Uterine caesarean closure techniques affect ultrasound findings and maternal outcomes: a systematic review and meta-analysis. BJOG. 2018;125(9):1097–108.
25. Osser OV, Jokubkiene L, Valentin L. Cesarean section scar defects: agreement between transvaginal sonographic findings with and without saline contrast enhancement. Ultrasound Obstet Gynecol. 2010;35(1):75–83.
26. Aaronson NK, Muller M, Cohen PD, Essink-Bot ML, Fekkes M, Sanderman R, Sprangers MA, te Velde A, Verrips E. Translation, validation, and norming of the Dutch language version of the SF-36 health survey in community and chronic disease populations. J Clin Epidemiol. 1998;51(11):1055–68.
27. Dolan P. Modeling valuations for EuroQol health states. Med Care. 1997; 35(11):1095–108.
28. EuroQol--a new facility for the measurement of health-related quality of life. Health Policy. 1990;16(3):199–208.
29. Ability to Participate in Social Roles and Activities, Short Form 8a [http://www.healthmeasures.net/explore-measurement-systems/promis/intro-to-promis/list-of-adult-measures].
30. Rosen R, Brown C, Heiman J, Leiblum S, Meston C, Shabsigh R, Ferguson D, D'Agostino R Jr. The female sexual function index (FSFI): a multidimensional self-report instrument for the assessment of female sexual function. J Sex Marital Ther. 2000;26(2):191–208.
31. Handleiding iMTA Productivity Cost Questionnaire (iPCQ). [http://www.imta.nl].
32. Handleiding iMTA Medical Cost Questionnaire (iMCQ). [http://www.imta.nl].
33. Maarse M, Bij de Vaate AJM, Huirne JAF, Brölmann HAM. De maandkalender, een hulpmiddel voor een efficiënte menstruatieanamnese. Ned Tijdschr Obstet Gynaecol. 2011;124:231–5.
34. Hakkaart-van Roijen L, van der Linden N, Bouwmans CAM, Kanters TA, Tan SS. Kostenhandleiding: Methodologie van kostenonderzoek en referentieprijzen voor economische evaluaties in de gezondheidszorg. Institute for Medical Technology Assessment; Erasmus Universiteit Rotterdam. Zorginstituut Nederland; 2016.
35. Lamers LM, Stalmeier PF, McDonnell J, Krabbe PF, van Busschbach JJ. Measuring the quality of life in economic evaluations: the Dutch EQ-5D tariff. Ned Tijdschr Geneeskd. 2005;149(28):1574–8.
36. Fenwick E, O'Brien BJ, Briggs A. Cost-effectiveness acceptability curves--facts, fallacies and frequently asked questions. Health Econ. 2004;13(5):405–15.
37. Glavind J, Madsen LD, Uldbjerg N, Dueholm M. Ultrasound evaluation of cesarean scar after single- and double-layer uterotomy closure: a cohort study. Ultrasound Obstet Gynecol. 2013;42(2):207–12.

Ready to submit your research? Choose BMC and benefit from:

- fast, convenient online submission
- thorough peer review by experienced researchers in your field
- rapid publication on acceptance
- support for research data, including large and complex data types
- gold Open Access which fosters wider collaboration and increased citations
- maximum visibility for your research: over 100M website views per year

At BMC, research is always in progress.

Learn more biomedcentral.com/submissions

BMC

For peer review only

CHEERS Checklist
Items to include when reporting economic evaluations of health interventions

The **ISPOR CHEERS Task Force Report**, *Consolidated Health Economic Evaluation Reporting Standards (CHEERS)—Explanation and Elaboration: A Report of the ISPOR Health Economic Evaluations Publication Guidelines Good Reporting Practices Task Force,* provides examples and further discussion of the 24-item CHEERS Checklist and the CHEERS Statement. It may be accessed via the *Value in Health* or via the ISPOR Health Economic Evaluation Publication Guidelines – CHEERS: Good Reporting Practices webpage: http://www.ispor.org/TaskForces/EconomicPubGuidelines.asp

Section/item	Item No	Recommendation	Reported on page No/ line No
**Title and abstract**			
Title	1	Identify the study as an economic evaluation or use more specific terms such as "cost-effectiveness analysis", and describe the interventions compared.	1
Abstract	2	Provide a structured summary of objectives, perspective, setting, methods (including study design and inputs), results (including base case and uncertainty analyses), and conclusions.	2
**Introduction**			
Background and objectives	3	Provide an explicit statement of the broader context for the study. Present the study question and its relevance for health policy or practice decisions.	4
**Methods**			
Target population and subgroups	4	Describe characteristics of the base case population and subgroups analysed, including why they were chosen.	6
Setting and location	5	State relevant aspects of the system(s) in which the decision(s) need(s) to be made.	7, 8
Study perspective	6	Describe the perspective of the study and relate this to the costs being evaluated.	7
Comparators	7	Describe the interventions or strategies being compared and state why they were chosen.	8
Time horizon	8	State the time horizon(s) over which costs and consequences are being evaluated and say why appropriate.	7
Discount rate	9	Report the choice of discount rate(s) used for costs and outcomes and say why appropriate.	7
Choice of health outcomes	10	Describe what outcomes were used as the measure(s) of benefit in the evaluation and their relevance for the type of analysis performed.	8-10
Measurement of effectiveness	11a	*Single study-based estimates:* Describe fully the design features of the single effectiveness study and why the single study was a sufficient source of clinical effectiveness data.	8, 9

	11b	*Synthesis-based estimates:* Describe fully the methods used for identification of included studies and synthesis of clinical effectiveness data.	NA
Measurement and valuation of preference based outcomes	12	If applicable, describe the population and methods used to elicit preferences for outcomes.	8 - 10
Estimating resources and costs	13a	*Single study-based economic evaluation:* Describe approaches used to estimate resource use associated with the alternative interventions. Describe primary or secondary research methods for valuing each resource item in terms of its unit cost. Describe any adjustments made to approximate to opportunity costs.	9, 10
	13b	*Model-based economic evaluation:* Describe approaches and data sources used to estimate resource use associated with model health states. Describe primary or secondary research methods for valuing each resource item in terms of its unit cost. Describe any adjustments made to approximate to opportunity costs.	NA
Currency, price date, and conversion	14	Report the dates of the estimated resource quantities and unit costs. Describe methods for adjusting estimated unit costs to the year of reported costs if necessary. Describe methods for converting costs into a common currency base and the exchange rate.	9, 10 table S1
Choice of model	15	Describe and give reasons for the specific type of decision-analytical model used. Providing a figure to show model structure is strongly recommended.	NA
Assumptions	16	Describe all structural or other assumptions underpinning the decision-analytical model.	NA
Analytical methods	17	Describe all analytical methods supporting the evaluation. This could include methods for dealing with skewed, missing, or censored data; extrapolation methods; methods for pooling data; approaches to validate or make adjustments (such as half cycle corrections) to a model; and methods for handling population heterogeneity and uncertainty.	10 - 12
**Results**			
Study parameters	18	Report the values, ranges, references, and, if used, probability distributions for all parameters. Report reasons or sources for distributions used to represent uncertainty where appropriate. Providing a table to show the input values is strongly recommended.	NA
Incremental costs and outcomes	19	For each intervention, report mean values for the main categories of estimated costs and outcomes of interest, as well as mean differences between the comparator groups. If applicable, report incremental cost-effectiveness ratios.	13 - 15 Table 2+3
Characterising uncertainty	20a	*Single study-based economic evaluation:* Describe the effects of sampling uncertainty for the estimated incremental cost and incremental effectiveness parameters, together with the impact	11

		of methodological assumptions (such as discount rate, study perspective).	
	20b	*Model-based economic evaluation:* Describe the effects on the results of uncertainty for all input parameters, and uncertainty related to the structure of the model and assumptions.	NA
Characterising heterogeneity	21	If applicable, report differences in costs, outcomes, or cost-effectiveness that can be explained by variations between subgroups of patients with different baseline characteristics or other observed variability in effects that are not reducible by more information.	12 ; 15
**Discussion**			
Study findings, limitations, generalisability, and current knowledge	22	Summarise key study findings and describe how they support the conclusions reached. Discuss limitations and the generalisability of the findings and how the findings fit with current knowledge.	16-18
**Other**			
Source of funding	23	Describe how the study was funded and the role of the funder in the identification, design, conduct, and reporting of the analysis. Describe other non-monetary sources of support.	19
Conflicts of interest	24	Describe any potential for conflict of interest of study contributors in accordance with journal policy. In the absence of a journal policy, we recommend authors comply with International Committee of Medical Journal Editors recommendations.	19, 20

For consistency, the CHEERS Statement checklist format is based on the format of the CONSORT statement checklist

The **ISPOR CHEERS Task Force Report** provides examples and further discussion of the 24-item CHEERS Checklist and the CHEERS Statement. It may be accessed via the *Value in Health* link or via the ISPOR Health Economic Evaluation Publication Guidelines – CHEERS: Good Reporting Practices webpage: http://www.ispor.org/TaskForces/EconomicPubGuidelines.asp

The citation for the CHEERS Task Force Report is:
Husereau D, Drummond M, Petrou S, et al. Consolidated health economic evaluation reporting standards (CHEERS)—Explanation and elaboration: A report of the ISPOR health economic evaluations publication guidelines good reporting practices task force. Value Health 2013;16:231-50.

BMJ Open

Cost-effectiveness of single- versus double-layer uterine closure during caesarean section on postmenstrual spotting: economic evaluation alongside a randomised controlled trial

Journal:	*BMJ Open*
Manuscript ID	bmjopen-2020-044340.R2
Article Type:	Original research
Date Submitted by the Author:	01-Jun-2021
Complete List of Authors:	Stegwee, Sanne; Amsterdam UMC Locatie VUmc, Obstetrics and Gynaecology<br>Ben, Ângela; Vrije Universiteit Amsterdam, Health Sciences<br>El Alili, Mohamed ; Vrije Universiteit Amsterdam<br>van der Voet, Lucet; Deventer Ziekenhuis, Obstetrics and Gynaecology<br>de Groot, Christianne; Amsterdam UMC Locatie VUmc, Obstetrics and Gynaecology<br>Bosmans, J; Vrije Universiteit Amsterdam, Health Sciences<br>Huirne, Judith; Amsterdam UMC Locatie AMC, Obstetrics and Gynaecology
**Primary Subject Heading**:	Obstetrics and gynaecology
Secondary Subject Heading:	Health economics
Keywords:	OBSTETRICS, Maternal medicine < OBSTETRICS, Ultrasonography < OBSTETRICS, HEALTH ECONOMICS

SCHOLARONE™
Manuscripts

I, the Submitting Author has the right to grant and does grant on behalf of all authors of the Work (as defined in the below author licence), an exclusive licence and/or a non-exclusive licence for contributions from authors who are: i) UK Crown employees; ii) where BMJ has agreed a CC-BY licence shall apply, and/or iii) in accordance with the terms applicable for US Federal Government officers or employees acting as part of their official duties; on a worldwide, perpetual, irrevocable, royalty-free basis to BMJ Publishing Group Ltd ("BMJ") its licensees and where the relevant Journal is co-owned by BMJ to the co-owners of the Journal, to publish the Work in this journal and any other BMJ products and to exploit all rights, as set out in our licence.

The Submitting Author accepts and understands that any supply made under these terms is made by BMJ to the Submitting Author unless you are acting as an employee on behalf of your employer or a postgraduate student of an affiliated institution which is paying any applicable article publishing charge ("APC") for Open Access articles. Where the Submitting Author wishes to make the Work available on an Open Access basis (and intends to pay the relevant APC), the terms of reuse of such Open Access shall be governed by a Creative Commons licence – details of these licences and which Creative Commons licence will apply to this Work are set out in our licence referred to above.

Other than as permitted in any relevant BMJ Author's Self Archiving Policies, I confirm this Work has not been accepted for publication elsewhere, is not being considered for publication elsewhere and does not duplicate material already published. I confirm all authors consent to publication of this Work and authorise the granting of this licence.

For peer review only

Cost-effectiveness of single- versus double-layer uterine closure during caesarean section on postmenstrual spotting: economic evaluation alongside a randomised controlled trial

Sanne I Stegwee[1]*, Ângela Jornada Ben[2], Mohamed El Alili[2], Lucet F van der Voet[3], Christianne JM de Groot[1], Judith E Bosmans[2], Judith AF Huirne[1]*, for the 2Close study group†

[1] Department of Obstetrics and Gynaecology, Amsterdam UMC, Vrije Universiteit, Amsterdam Reproduction & Development, Amsterdam, Netherlands

[2] Department of Health Sciences, Amsterdam Public Health, Faculty of Science, VU University, Amsterdam, The Netherlands

[3] Department of Obstetrics and Gynaecology, Deventer Hospital, Deventer, Netherlands

*Corresponding author during submission: SI Stegwee, MD, Amsterdam UMC - VU University, Department of Obstetrics and Gynaecology, De Boelelaan 1117, 1081 HV Amsterdam, The Netherlands. T: +31 20 4444807, F: +31 20 444 83 87, s.stegwee@amsterdamumc.nl.

*Corresponding author after publication: Prof JAF Huirne, Amsterdam UMC - VU University, Department of Obstetrics and Gynaecology, De Boelelaan 1117, 1081 HV Amsterdam, The Netherlands. T: +31 20 4444444, F: +31 20 444 83 87, j.huirne@amsterdamumc.nl.

†A full list of collaborators is provided in the appendix

For peer review only - http://bmjopen.bmj.com/site/about/guidelines.xhtml

Abstract

Objective: To evaluate the cost-effectiveness of double-layer compared to single-layer uterine closure after a first caesarean section (CS) from a societal and healthcare perspective.

Design: Economic evaluation alongside a multicentre, double-blind, randomised controlled trial

Setting: 32 hospitals in the Netherlands, 2016-18.

Participants: 2292 women ≥ 18 years undergoing a first CS were randomly assigned (1:1). Exclusion criteria were: inability for counselling, previous uterine surgery, known menstrual disorder, placenta increta or percreta, pregnant with three or more fetuses. 1144 women were assigned to single-layer and 1148 to double-layer closure. We included 1620 women with a menstrual cycle in the main analysis.

Interventions: Single-layer unlocked uterine closure and double-layer unlocked uterine closure with the second layer imbricating the first.

Main outcome measures: Spotting days, quality-adjusted life-years (QALYs), and societal costs at nine months of follow-up. Missing data were imputed using multiple imputation.

Results: No significant differences were found between single- versus double-layer closure in mean spotting days (1.44 and 1.39 days; mean difference [md] -0.056, 95% CI, -0.374; 0.263), QALYs (0.663 and 0.658; md -0.005, 95% CI, -0.015; 0.005), total healthcare costs (€744 and €727; md €-17, 95% CI, -273; 143), and total societal costs (€5689 and €5927; md €238, 95% CI, -624; 1108). The probability of the intervention being cost-effective at willingness-to-pay of 0, 10,000 and 20,000 €/QALY gained was 0.30, 0.27 and 0.25, respectively, (societal perspective), and 0.55, 0.41 and 0.32, respectively, (healthcare perspective).

Conclusion: Double-layer uterine closure is not cost-effective compared to single-layer uterine closure from both perspectives. If this is confirmed by our long-term reproductive follow-up, we suggest to adjust uterine closure technique guidelines.

Trial registration: Netherlands Trial Register (NTR5480/NL5380; trial finished).

Key words: caesarean section; uterine closure technique; postmenstrual spotting; cost-effectiveness analysis

For peer review only - http://bmjopen.bmj.com/site/about/guidelines.xhtml

Article summary

Strengths and limitations of this study

This study has been performed alongside large multicentre RCT, which is considered the best vehicle for economic evaluations

We prospectively collected data regarding costs and effect, and we used patient level information

All relevant costs for decision making were included in the analysis, to conduct an analysis from a societal perspective

Possible recall bias due to retrospective self-reported questionnaire over 3- and 6-month period

Generalizability of the results to other populations may be limited, as other healthcare practices and payment systems may exist

For peer review only

For peer review only - http://bmjopen.bmj.com/site/about/guidelines.xhtml

Introduction

Caesarean section (CS) rates rise globally and is the mode of delivery for approximately one in five live births globally.[1 2] As a consequence, a rise in morbidity related to CS is observed as well.[3] Severe morbidity associated with a subsequent pregnancy includes caesarean scar pregnancy, placenta accreta spectrum disorders and uterine rupture. However, less severe but more prevalent gynaecological morbidity related to a CS have recently gained more interest as well. Chronic maternal morbidity after CS includes dysmenorrhoea and abnormal uterine bleeding, which are both associated with a sonographically visible indentation at the site of the previous uterine incision.[4-6] This indentation is called a niche and is seen in approximately 60% of women after CS.[7 8] Of them, 30% develops abnormal uterine bleeding and more specifically, postmenstrual spotting.[5 6] This is brownish discharge at the end of the menstruation or blood loss in between two menstruations that is limiting women in daily life.[5] Over the last years, an increase in the development of medical treatments and surgical procedures to treat or remove the niche is observed, primarily aiming to reduce spotting.[9]

CS is the most common major surgical intervention.[10] However, there is no international guideline on the most optimal way to close the uterine incision while the specific closure technique may influence healing of the uterine wound. A specific issue on which no consensus exists is whether to use single- or double-layer closure of the uterine layers.[11 12] When comparing these techniques, no differences were found at short-term except for longer operation time after double-layer closure.[13 14] Nevertheless, previous studies also suggested that double-layer closure may result in better uterine scar healing and lower prevalence of large niches thereby possibly leading to lower medical costs than single-layer closure.[13 15] There is, however, a lack of studies on uterine closure techniques and their impact on maternal health outcomes related to gynaecological symptoms.[14] The impact of different uterine closure techniques on healthcare utilization, informal care, and lost productivity costs has never been investigated previously. As decision makers increasingly demand evidence of cost-effectiveness of health care interventions, conducting economic analysis alongside clinical trials is desirable because it allows the prospective collection of cost and effect data and the use of patient

For peer review only - http://bmjopen.bmj.com/site/about/guidelines.xhtml

level information for drawing inferences about additional costs and benefits of interventions.[16] In addition, regulatory and reimbursement agencies of many countries consider evidence of economic value along with clinical effectiveness.[16]

Therefore, the aim of this study was to perform a cost-effectiveness analysis of double-layer compared to single-layer uterine closure after a first CS from both a societal and healthcare perspective. We hypothesised that double-layer closure would reduce postmenstrual spotting and total societal and healthcare costs compared to single-layer closure as a result of less morbidity, despite slightly higher intervention costs of double-layer closure.

For peer review only

For peer review only - http://bmjopen.bmj.com/site/about/guidelines.xhtml

Methods

Study design

An economic evaluation was performed alongside a multicentre randomised controlled superiority trial comparing double-layer closure and single-layer uterine closure after a first CS. The study protocol and the effect paper have been published elsewhere[17 18] The study was approved by the Institutional Review Board (IRB) of Amsterdam UMC – location VU University medical centre (registration number 2015.462) and by the boards of all participating hospitals. No substantial changes were made to the protocol after commencement of the trial.[17] This trial-based economic evaluation is reported according to the Consolidated Health Economic Evaluating Reporting Standards (CHEERS) statement.[19]

Target population

All women who underwent a first CS, planned or unplanned, at one of the participating hospitals were asked to participate in the study. Inclusion criteria were sufficient command of the Dutch or English language, age 18 years or older and written informed consent. Exclusion criteria were: inadequate possibility for counselling (e.g., indication for emergency CS without being informed about the study previously, women in severe pain without adequate therapy), previous major uterine surgery (e.g., laparoscopic or laparotomic fibroid resection, septum resection), women with known causes of menstrual disorders (e.g., cervical dysplasia, communicating hydrosalpinx, uterine anomaly or endocrine disorders disturbing ovulation), placenta increta or percreta during the current pregnancy, or three or more foetuses during the current pregnancy. After informed consent had been signed and a CS was indicated, participants were randomly allocated to receive single-layer (control) or double-layer (intervention) closure of the uterine incision in a 1:1 ratio. Due to the nature of the treatment, surgeons performing the CS were not masked to the allocated method. Participants and sonographers were blinded to the allocation, researchers and statisticians were not. Detailed information about study design and randomization can be found in the study protocol.[17]

Choice of health outcomes

For peer review only

For this trial-based economic evaluation, two main health outcomes were used: postmenstrual spotting (referred to as spotting days in this paper) and Quality-Adjusted Life-Years (QALYs) at nine months after CS. Spotting days was chosen because it has been strongly related to a niche[5 6] (i.e., an indentation at the site of the caesarean scar with a depth of at least 2 mm[20]), which may be influenced by uterine closure technique.[8] QALY is routinely used as a summary outcome measure of health in economic evaluations, because it incorporates the impact of interventions on both the quantity and quality of life,[21] and allows decision makers to compare the effectiveness and cost-effectiveness of a range of interventions for different health conditions.[22]

Study perspective and time-horizon

This trial-based economic evaluation was performed from a societal and a healthcare perspective over a time-horizon of nine months. Therefore, discounting of costs and effects was not necessary. When a healthcare perspective is adopted, only the intervention costs and costs related to healthcare utilization are included in the analysis.[23] For the societal perspective, costs related to informal care and productivity losses are included in addition to intervention and healthcare utilization costs.[23]

Setting and location

In total, 32 hospitals in the Netherlands, both academic (n=6) and non-academic (n=26), collaborating within the Dutch Consortium for Healthcare Evaluation and Research in Obstetrics and Gynaecology (NVOG Consortium 2.0, www.zorgevaluatienederland.nl), participated in this study.[17] In the Netherlands, a CS is only performed in a hospital setting. In most cases, without maternal or neonatal complications, women will be discharged from the hospital after a CS after two or three days. All costs regarding the CS and admission days are standard care and are paid by an individual's health insurance. Maternity leave of at least ten weeks is regulated through the Employee Insurance Agency. Paternity leave is limited to one week. The first eight days after delivery, a maternity nurse visits the family at least three hours a day.

Control and intervention condition

For peer review only - http://bmjopen.bmj.com/site/about/guidelines.xhtml
For peer review only

The control group underwent single-layer closure of the uterus using unlocked continuous running multifilament sutures, which is the usual care provided by hospitals in the Netherlands.[17] In the intervention group, double-layer closure of the uterine incision was performed using unlocked multifilament continuous running sutures for both layers and the endometrial layer was included in the first layer. The second layer was a continuous running suture that imbricated the first. A mandatory online instruction video was shown to all surgeons in participating hospitals prior to participation for the intervention group. The exact procedures in both study arms regarding uterine closure are described in the study protocol.[17]

At baseline, data was collected on sociodemographic characteristics for all participants.[17]

Outcomes

Health outcomes

Spotting days was the primary outcome of the trial, and was defined as number of days with brownish discharge for more than two days at the end of the menstruation, with a total duration (menstruation and spotting) of more than seven days, or intermenstrual blood loss that started after the end of the menstruation.[5] Spotting days were self-reported by participants through a digital questionnaire at nine months after CS, including a calendar on which women could record daily blood loss during one month. Women who reported that they had no blood loss were classified as amenorrhoeic.

Health-related quality of life was measured using the EQ-5D-5L at baseline, and at three and nine months after CS.[24] The EQ-5D-5L has five dimensions of quality of life (mobility, self-care, usual activities, pain/discomfort, and anxiety/depression) using five response levels (i.e., no problems, slight problems, moderate problems, severe problems, or extreme problems) describing 234 health states.[24] The participants' health states obtained from EQ-5D-5L responses were converted into utility values using the Dutch tariff.[25] The utility values were used to calculate QALYs by means of the area under the curve method (i.e., the duration of a health state is multiplied by the utility related to that health state).

For peer review only - http://bmjopen.bmj.com/site/about/guidelines.xhtml

Cost outcomes

Intervention costs

The average costs of performing a CS reported by the participating hospitals was €5360. The intervention incurred additional suture material and additional operation time (3.9 minutes on average). The costs of additional resources were obtained from the academic and non-academic hospitals using a bottom-up approach. On average, the intervention resulted in additional costs of €95.79 per participant in academic hospitals and €71.14 per participant in non-academic hospitals (Supplementary Table S1).

Healthcare utilization and informal care costs

A specifically adapted version of the iMTA Medical Cost Questionnaire (iMCQ)[26] was used to measure healthcare utilization and care provided by family and/or friends (i.e., informal care) using 3-month and 6-month recall periods at 3 and 9 months of follow-up, respectively. The iMCQ is a standardized generic instrument for measuring medical costs including questions related to healthcare utilization and informal care.[26] Healthcare utilization was valued using prices from the Dutch costing guideline.[27] Healthcare ultilization costs included primary care costs (e.g., costs of visits to general practitioners, health professionals, and complementary healthcare providers), secondary care costs (e.g., costs of ambulatory hospital visits, visits to other healthcare organizations and admissions to the hospital), and medication costs. Secondary care costs were recorded after discharge from the hospital. The average costs of performing the CS included the operation and hospital stay until discharge and were, therefore, not included in the secondary care costs to avoid double-counting.

The informal care costs were based on the amount of time the participant needed help in performing household tasks or received care from family and/or friends, because of health problems. Dutch standard prices were used for informal care costs.[27] Medication use was valued using data from the Dutch Healthcare Institute (www.medicijnkosten.nl).[28]

Lost productivity costs

For peer review only - http://bmjopen.bmj.com/site/about/guidelines.xhtml

For peer review only

The iMTA Productivity Cost Questionnaire (iPCQ)[29] was used to measure self-reported sickness absenteeism from paid and unpaid work, and presenteeism using 3-month and 6-month recall periods at 3 and 9 months of follow-up, respectively. The iPCQ is a standardized generic questionnaire to measure productivity costs and it is applicable to national and international studies.[29] The friction cost approach (FCA) was used to calculate sickness absenteeism costs from paid work.[30] The FCA assumes that sickness absenteeism costs are limited to the period needed to replace an absent, sick worker (the friction period), which has been estimated to be 12 weeks (85 days) in the Netherlands.[27] Gender-specific estimates of the mean wages of the Dutch population were used to calculate sickness absenteeism costs from paid work.[27]

To measure sickness absenteeism from unpaid work, the participants were asked whether they had difficulty in performing unpaid work activities due to sickness (e.g., household tasks, childcare, voluntary work), and if that was the case, for how many hours.[29] Costs related to sickness absenteeism from unpaid work were valued using a shadow price for legally employing a domestic assistant.[29] To measure participants' level of presenteeism, participants rated how efficiently they worked while suffering from health complaints on a scale from 0 (I was unable to do anything) to 10 (I was able to do as much as usual). The resulting efficiency score was used to calculate presenteeism costs: Presenteeism costs = number of days working with complaints * [1 - (efficiency score / 10)] * number of working hours per day * gender-specific mean wage rates.[31]

For peer review only

Statistical analysis

The main analyses included all participants with a menstrual cycle (i.e., participants with amenorrhoea for any reason were excluded) at 9 months follow-up.

Analyses were performed according to the intention-to-treat principle using StataSE 16® (StataCorp LP, CollegeStation, TX, US). Multiple Imputation by Chained Equations (MICE), stratified by group allocation, was used to impute missing data. Variables associated with missingness and outcomes as well as potential confounders were included in the imputation model such as age, BMI, level of

For peer review only - http://bmjopen.bmj.com/site/about/guidelines.xhtml

education, parity, previous miscarriage, gestational age at CS, hypertension, diabetes, use of contraception, and breastfeeding. Predictive Mean Matching was used in the imputation procedure to account for the skewed distribution of the costs.[32] The number of imputations was increased until there was a loss of efficiency of ≤5%, resulting in twenty imputed datasets.[32 33] The twenty datasets were analyzed separately and estimates were pooled using Rubin's rules.[34] After multiple imputation, amenorrhoeic women were excluded from the analyses as a priori decided, because the outcome spotting days could not be evaluated in these women.[17]

Differences in costs and effects between treatment groups at nine months follow-up were estimated using seemingly unrelated regression analyses, which accounts for the correlation between costs and effects.[35] The intraclass correlation coefficient (ICC, i.e., the variation around the subjects belonging to the same hospital cluster divided by the total variance between hospitals)[36] was small (ICC=0.004). This means that hardly any of the variance in the outcome measure was accounted for by clustering at the level of the hospital. In addition to the small ICC, patients were randomised at the individual level and not at the hospital level. Therefore, multilevel analysis was not necessary. Incremental Cost-Effectiveness Ratios (ICERs) were calculated by dividing the difference in costs (i.e., total societal costs and total healthcare costs) between groups by the difference in effects.

Bias-corrected accelerated bootstrapping with 5,000 replications was used to estimate the joint uncertainty surrounding differences in costs and effects. Bootstrapped cost-effect pairs were plotted on cost-effectiveness planes (CE-planes).[37] Cost-effectiveness acceptability curves (CEACs) were estimated that show the probability of double-layer closure being cost-effective compared to single-layer closure for a range of willingness-to-pay (WTP) thresholds (i.e., the maximum amount of money society is willing to pay for a unit of effect gained).[38] For spotting days, we used a maximum WTP threshold of €253 per one day reduction. This threshold was based on the value of eight hours of paid work given the average productivity costs per working hour for women in the Netherlands (i.e., €31.6 per hour). For QALYs, we used a WTP threshold of €20,000/QALY gained recommended by the Dutch

For peer review only - http://bmjopen.bmj.com/site/about/guidelines.xhtml

Health Care Institute[27] and €23,420/QALY (equivalent of £20,000/QALY) recommended by the National Institute for Health and Clinical Excellence (NICE).[39]

Sensitivity analysis

Four sensitivity analyses (SA) were performed to assess the robustness of the results. SA1 consisted of a CEA including all women randomised (i.e., without excluding amenorrhoeic women) from both a societal and healthcare perspective for the QALY outcome. SA2 consisted of a complete case analysis from both a societal and healthcare perspective including only women without amenorrhoea for both spotting days and QALYs. Third, we performed per protocol analyses for both outcomes from both a societal and healthcare perspective (SA3). Finally, we performed a sensitivity analysis in which we adjusted for hormonal contraception and breastfeeding (exclusively or combined with formula) at 9 months of follow-up (SA4).

Patient and public involvement

The Dutch gynaecologic patients' association agreed upon the design of the study and the grant proposal for funding. They were not involved in outcome measures or recruitment, and they were not asked to give advice in the interpretation of the results. We will disseminate the study results to all participants, and to the public through popular science articles.

For peer review only

Results

Participants

In total, 2292 women undergoing a first CS were included. Of them, 1144 participants were randomised to single-layer and 1148 participants to double-layer closure of the uterine incision. In the single-layer group, 694 (60.7%) participants underwent planned CS, and in the double-layer group this was done in 705 (61.4%) participants.

In total, 672 women reported amenorrhoea (n=331 in control and n=341 in intervention group), resulting in 1620 women reported having menstrual blood loss over nine months of follow-up and were included in the main analyses (n=813 in control and n=807 in intervention group) [Figure 1]. Of all women included in the main analysis (n=1620), 95% had completed follow-up data for spotting days (n=1544, 774 in control group and 770 in intervention group). Within the total group (n=2292), complete follow-up data was available for 74% of QALYs (n=1696, 851 in control group and 845 in intervention group), for 72% of total healthcare costs (n=1653, 823 in control group and 830 in intervention group), and for 58% of total societal costs (n=1335, 671 in control group and 664 in intervention group)[Figure 1]. At baseline, no meaningful differences were found between both groups (Table 1). At 9 months follow-up, 12.1% of women in the single-layer arm and 17.1% of women in the double-layer arm were breastfeeding their child(ren). In the single-layer arm, 40.3% of the participants used hormonal contraceptives at 9 months follow-up, and in the double-layer arm this was 38.2%. Participants with complete follow-up were more likely to be nulliparous and to have a higher education level compared to participants without complete follow-up.

Effectiveness

There were no statistically significant differences between groups in spotting days (mean difference -0.056, 95% CI, -0.374, 0.263) and QALYs (mean difference -0.005, 95% CI, -0.015; 0.005) at nine months follow-up (Table 2).

Costs

For peer review only - http://bmjopen.bmj.com/site/about/guidelines.xhtml

The main contributors to total societal costs in both groups were lost productivity costs (€5689 in control group and €5927 in intervention group) and healthcare costs (€744 in control group and €727 in intervention group, Table 2). There were no differences in days of hospital stay between the groups.[18] Costs associated with absenteeism from unpaid work due to sickness (i.e., costs related to getting help to perform household tasks, childcare, voluntary work) was the largest contributor to lost productivity costs in both groups (€3525 in control group and €3810 in intervention group). Secondary care costs were the largest contributor to total healthcare costs in both groups (€400 in control, group and €317 in intervention group). During follow-up, there were no statistically significant differences between groups in total healthcare costs (€-17, 95% CI, -273; 143) and total societal costs (€238, 95% CI, -624; 1108, Table 2). Presenteeism costs were not statistically significantly different in the intervention group compared to the control group (€-34, 95% CI, -98; 28, Table 2).

Cost-effectiveness analysis

From a societal perspective, most bootstrapped cost-effect pairs (44%) were in the North-East quadrant of the CE-plane for spotting days (Table 3, Figure 2 [1A]). The probability of double-layer closure being cost-effective compared to single-layer was 0.30 at a WTP of €0/spotting day less and 0.31 at €253/spotting day less (Figure 2 [2A]). For QALYs, most of the bootstrapped cost-effect pairs (62%) was in the north-west quadrant of the CE-plane (Table 3, Figure 2 [1B]). The probability of double-layer closure being cost-effective compared to single-layer at both the Dutch WTP threshold of €20,000/QALY gained, and the UK WTP threshold of €23,420/QALY gained, was 0.25 from a societal perspective (Figure 2 [2B], supplementary table S2).

From a healthcare perspective, bootstrapped cost-effect pairs were equally distributed among the eastern and western quadrants of the CE-plane for spotting days (Table 3, Figure 2 [1C]). This shows that uncertainty around costs and effects is large. The CEA curve presented in figure 2 [2C] shows that if the WTP for one spotting day less is €0, the probability of double-layer closure being cost-effective in comparison with single-layer was 0.55. This probability increases to 0.59 if the WTP is €253/spotting day less (Supplementary table S2). For QALYs, from a healthcare perspective, most of bootstrapped

For peer review only - http://bmjopen.bmj.com/site/about/guidelines.xhtml

cost-effect pairs were in the north-western quadrants of the CE-plane (Table 3, Figure 2 [1D]). The probability of double-layer closure being cost-effective compared to single-layer closure at the Dutch and UK WTP threshold (€20,000 and €23,420/QALY gained, respectively) was 0.41 from a healthcare perspective (Table 3, Figure 2 [1D]).

The results of the sensitivity analyses were similar to those of the main analysis (Table 3).

For peer review only

For peer review only - http://bmjopen.bmj.com/site/about/guidelines.xhtml

Discussion

The results of this trial-based economic evaluation showed that double-layer uterine closure after a first CS did not significantly decrease spotting days nor improve QALYs compared to single-layer at nine months follow-up. In addition, total healthcare costs and societal costs related to double-layer closure did not significantly differ from single-layer closure. Low probabilities of double-layer closure being cost-effective in comparison with single-layer closure were found for all relevant willingness-to-pay thresholds. Therefore, double-layer closure was not considered cost-effective compared to single-layer closure after a first CS from a societal and a healthcare perspective.

Comparison with previous studies

The results of this economic evaluation are not in line with our hypothesis, which was based on previously conducted observational studies[5 6 40] and meta-analyses.[14] These showed that single-layer closure resulted in thinner residual myometrium and a higher proportion of large niches than double-layer closure. These sonographic findings, or surrogates, were suggested to lead to more postmenstrual spotting and, therefore, higher related costs. Although double-layer closure resulted in increased CS costs, these costs were neutralised by higher secondary care and presenteeism costs in the single-layer closure group, resulting in no overall difference in total health care costs or total societal costs.

To the best of our knowledge, this is the first study evaluating the cost-effectiveness of double-layer closure in comparison with single-layer closure after a CS. The largest study (n=15,935) on this topic mentioned possible cost savings but no cost-effectiveness analysis was performed.[41] The second largest study (n=3033) comparing single- versus double-layer closure in a factorial RCT hypothesised on a possible reduction in costs in their study protocol, since a CS is conducted so frequently that *'any difference in morbidity is likely to have significant cost and community effects'*. The authors found a difference in operative time, though they did not discuss costs in the 2010 publication, and it is unlikely that a cost-effectiveness analysis is going to be performed.[42]

For peer review only - http://bmjopen.bmj.com/site/about/guidelines.xhtml

Based on the current and previous studies[43 44], we recommend to leave the choice of uterine closure technique with the preference of the surgeon. Previous studies reported only short-term maternal outcomes in the first few weeks after CS.[42-44] We confirmed these findings using a follow-up of nine months after CS. In addition, we showed that there is no difference in costs between the two types of closure. Our three-years follow-up results will show whether double-layer is superior compared to single-layer closure with regard to long-term outcomes. These outcomes include fertility outcomes, pregnancy complications and mode of delivery, as well as safety outcomes such as uterine dehiscence or rupture and related neonatal and maternal morbidity. In addition, a cost-effectiveness analysis for long-term outcomes will be performed as well. When superiority cannot be shown on the long-term either, guidelines should recommend to leave the uterine closure technique regarding single- versus double-layer up to the preference of the performing surgeon.

Strengths and limitations

This study was performed alongside a large multicenter randomised controlled superiority trial, which is considered the best vehicle for economic evaluations because it allows the prospective collection of cost and effect data and the use of patient level information for drawing inferences about additional costs and benefits of interventions.[16] Additionally, the CEA was conducted from a societal perspective meaning that all relevant costs for decision making (i.e., intervention, healthcare utilization, informal care and lost productivity costs) were included in the analysis.[22] Several sensitivity analyses were performed, to assess the robustness of our results, which resulted in similar results as compared to the main analyses.

However, one of the limitations of this study was that the cost questionnaires included retrospective self-reported questions over a 3-month and 6-month period, which may have caused recall bias. Nevertheless, we assume that this bias is equally distributed across the two groups and does, therefore, does not impact the difference between groups. Although there is no gold standard for measuring lost productivity costs, we used a standardised instrument[29], which is considered best

For peer review only - http://bmjopen.bmj.com/site/about/guidelines.xhtml

practice currently.[45] Another limitation is that generalizability of the results to healthcare systems in other countries may be limited, as they may adopt different usual practices and have different payment systems.[46] Additionally, generalizability may be impaired since in our study sample relatively many planned CS were performed compared to the Dutch average[47], probably resulting in an overall underestimation of niche related postmenstrual spotting.

Future research

It is important to realise that we have only evaluated the aspect of single-versus double-layer closure of the uterine incision in our trial. A CS consists of multiple steps and other aspects of the surgical technique used to perform a CS may also affect clinical outcomes and costs, and should therefore be subject to future research. Examples of uterine incision and repair are the level of hysterotomy (above or below the plica vesicouterina)[48] and inclusion or exclusion of the endometrium in the uterine suture.

Conclusions and policy implications

In conclusion, double-layer uterine closure is not cost-effective compared to single-layer uterine closure from both a societal and healthcare perspective. Thus, from a cost-effectiveness point of view, there is no reason to advocate double-layer over single-layer uterine closure. Long-term follow-up will show whether guidelines should be adapted based on obstetric and reproductive outcomes of double-layer closure compared to single-layer closure.

For peer review only

Acknowledgments

This study was performed with funding from ZonMw: The Netherlands Organisation for Health Research and Development (project number 843002605). We would like to thank all participants of the 2Close study. Additionally, we would like to thank the Departments of Obstetrics and Gynaecology of all participating hospitals, with special thanks to all research nurses and research midwives for their contribution in data collection.

The following are members of the 2Close study group: WM (Marchien) van Baal, Erik van Beek, Mireille N Bekker, Ângela Jornada Ben, Karin de Boer, Elisabeth MA Boormans, Judith E Bosmans, Hugo WF van Eijndhoven, Mohamed El Alili, AH (Hanneke) Feitsma, Christianne JM de Groot, Majoie Hemelaar, Wouter JK Hehenkamp, Wietske Hermes, Esther Hink, Judith AF Huirne, Anjoke JM Huisjes, CAH (Ineke) Janssen, Kitty Kapiteijn, Mesrure Kaplan, Paul JM van Kesteren, Judith OEH van Laar, Josje Langenveld, Wouter J Meijer, Angèle LM Oei, Eva Pajkrt, Dimitri NM Papatsonis, Celine M Radder, Robbert JP Rijnders, HCJ (Liesbeth) Scheepers, Daniela H Schippers, Nico WE Schuitemaker, Sanne I Stegwee, Marieke Sueters, Harry Visser, Huib AAM van Vliet, LHM (Marloes) de Vleeschouwer, Lucet F van der Voet

Contributors: SIS collected data, interpreted data and drafted the report. AJB and MEA analysed and interpreted data and drafted the report. LFvdV designed the study, collected data and revised the first draft of the report critically. CJMdG designed the study and revised the first draft of the report critically. JEB designed the study, interpreted data and participated in drafting and revising the report. JAFH designed the study, was principle investigator, interpreted the data and participated in drafting and revising the report. All authors approved the final version of the manuscript.

Funding: This study was performed with funding from ZonMw: The Netherlands Organisation for Health Research and Development (project number 843002605). The funder of the study approved the study protocol. The funder had no role in study design, data collection, data analysis, data interpretation, or writing of the manuscript. The corresponding author had full access to all the data in the study and had final responsibility for the decision to submit for publication.

For peer review only

For peer review only - http://bmjopen.bmj.com/site/about/guidelines.xhtml

Competing interests: JAFH received grants from ZonMw, during the conduct of the study; and reports grants from Samsung, grants from PlantTec Medical GmbH, and received a fee from Olympus, all outside the submitted work. CJMdG received a grant from ZonMw outside the submitted work. The other authors declare no competing interests.

Ethical approval: The study was approved by the Institutional Review Board (IRB) of Amsterdam UMC – location VU University medical centre in December 2015 (registration number 2015.462) and by the boards of all participating hospitals before start of inclusion. No substantial changes were made to the protocol after commencement of the trial. All participants provided written informed consent before taking part in the study.

Data sharing: De-identified individual participant data collected during the 2Close trial will be shared at one year after publication of the long-term results on request (j.huirne@amsterdamumc.nl). Approval of a proposal will be necessary before data will be shared. To gain access, requesters will need to sign an agreement form and confirm that data will be used for the purpose for which access was granted.

The corresponding and lead author (JAFH) affirms that the manuscript is an honest, accurate, and transparent account of the study being reported; no important aspects of the study have been omitted; any discrepancies from the study as originally planned in the registered study protocol have been explained. The corresponding author (JAFH) had the final responsibility for the decision to submit for publication.

This is an open access article distributed in accordance with the Creative Commons Attribution Non Commercial (CC BY-NC 4.0) license, which permits others to distribute, remix, adapt, build upon this work non-commercially, and license their derivative works on different terms, provided the original work is properly cited and the use is non-commercial. See: http://creativecommons.org/licenses/by-nc/4.0.

For peer review only - http://bmjopen.bmj.com/site/about/guidelines.xhtml

References

1. Boerma T, Ronsmans C, Melesse DY, et al. Global epidemiology of use of and disparities in caesarean sections. *Lancet* 2018;392(10155):1341-48. doi: 10.1016/s0140-6736(18)31928-7 [published Online First: 2018/10/17]
2. Betran AP, Ye J, Moller AB, et al. The Increasing Trend in Caesarean Section Rates: Global, Regional and National Estimates: 1990-2014. *PLoS One* 2016;11(2):e0148343. doi: 10.1371/journal.pone.0148343 [published Online First: 2016/02/06]
3. Marshall NE, Fu R, Guise JM. Impact of multiple cesarean deliveries on maternal morbidity: a systematic review. *Am J Obstet Gynecol* 2011;205(3):262.e1-8. doi: 10.1016/j.ajog.2011.06.035 [published Online First: 2011/11/11]
4. Wang CB, Chiu WW, Lee CY, et al. Cesarean scar defect: correlation between Cesarean section number, defect size, clinical symptoms and uterine position. *Ultrasound Obstet Gynecol* 2009;34(1):85-9. doi: 10.1002/uog.6405 [published Online First: 2009/07/01]
5. Bij de Vaate AJ, Brolmann HA, van der Voet LF, et al. Ultrasound evaluation of the Cesarean scar: relation between a niche and postmenstrual spotting. *Ultrasound Obstet Gynecol* 2011;37(1):93-9. doi: 10.1002/uog.8864 [published Online First: 2010/10/30]
6. van der Voet LF, Bij de Vaate AM, Veersema S, et al. Long-term complications of caesarean section. The niche in the scar: a prospective cohort study on niche prevalence and its relation to abnormal uterine bleeding. *BJOG* 2014;121(2):236-44. doi: 10.1111/1471-0528.12542 [published Online First: 2014/01/01]
7. Roberge S, Boutin A, Chaillet N, et al. Systematic review of cesarean scar assessment in the nonpregnant state: imaging techniques and uterine scar defect. *Am J Perinatol* 2012;29(6):465-71. doi: 10.1055/s-0032-1304829 [published Online First: 2012/03/09]
8. Bij de Vaate AJ, van der Voet LF, Naji O, et al. Prevalence, potential risk factors for development and symptoms related to the presence of uterine niches following Cesarean section: systematic review. *Ultrasound Obstet Gynecol* 2014;43(4):372-82. doi: 10.1002/uog.13199 [published Online First: 2013/09/03]
9. van der Voet LF, Vervoort AJ, Veersema S, et al. Minimally invasive therapy for gynaecological symptoms related to a niche in the caesarean scar: a systematic review. *BJOG* 2014;121(2):145-56. doi: 10.1111/1471-0528.12537 [published Online First: 2014/01/01]
10. Eurostat. Surgical operations and procedures statistics - statistics explained. . 03 Oct 2019. https://ec.europa.eu/eurostat/statisticsexplained/ (accessed 18 Nov 2019).
11. Caughey AB, Wood SL, Macones GA, et al. Guidelines for intraoperative care in cesarean delivery: Enhanced Recovery After Surgery Society Recommendations (Part 2). *Am J Obstet Gynecol* 2018;219(6):533-44. doi: 10.1016/j.ajog.2018.08.006 [published Online First: 2018/08/18]
12. NICE. Caesarean section - clinical guideline. . 01 Nov 2011. https://www.nice.org.uk/guidance/cg132 (accessed 19 Nov 2019).
13. Roberge S, Demers S, Berghella V, et al. Impact of single- vs double-layer closure on adverse outcomes and uterine scar defect: a systematic review and metaanalysis. *Am J Obstet Gynecol* 2014;211(5):453-60. doi: 10.1016/j.ajog.2014.06.014 [published Online First: 2014/06/10]
14. Stegwee SI, Jordans I, van der Voet LF, et al. Uterine caesarean closure techniques affect ultrasound findings and maternal outcomes: a systematic review and meta-analysis. *BJOG* 2018;125(9):1097-108. doi: 10.1111/1471-0528.15048 [published Online First: 2017/12/08]
15. Sevket O, Ates S, Molla T, et al. Hydrosonographic assessment of the effects of 2 different suturing techniques on healing of the uterine scar after cesarean delivery. *Int J Gynaecol Obstet* 2014;125(3):219-22. doi: 10.1016/j.ijgo.2013.11.013 [published Online First: 2014/04/01]

For peer review only

16. Ramsey SD, Willke RJ, Glick H, et al. Cost-effectiveness analysis alongside clinical trials II-An ISPOR Good Research Practices Task Force report. *Value Health* 2015;18(2):161-72. doi: 10.1016/j.jval.2015.02.001 [published Online First: 2015/03/17]
17. Stegwee SI, Jordans IPM, van der Voet LF, et al. Single- versus double-layer closure of the caesarean (uterine) scar in the prevention of gynaecological symptoms in relation to niche development - the 2Close study: a multicentre randomised controlled trial. *BMC Pregnancy Childbirth* 2019;19(1):85. doi: 10.1186/s12884-019-2221-y [published Online First: 2019/03/06]
18. Stegwee S, van der Voet L, Ben A, et al. Effect of single- versus double-layer uterine closure during caesarean section on postmenstrual spotting (2Close): multicentre, double-blind, randomised controlled superiority trial. *BJOG* 2020 doi: 10.1111/1471-0528.16472
19. Husereau D, Drummond M, Petrou S, et al. Consolidated Health Economic Evaluation Reporting Standards (CHEERS) statement. *BMJ* 2013;346:f1049. doi: 10.1136/bmj.f1049 [published Online First: 2013/03/27]
20. Jordans IPM, de Leeuw RA, Stegwee SI, et al. Sonographic examination of uterine niche in non-pregnant women: a modified Delphi procedure. *Ultrasound Obstet Gynecol* 2019;53(1):107-15. doi: 10.1002/uog.19049 [published Online First: 2018/03/15]
21. Whitehead SJ, Ali S. Health outcomes in economic evaluation: the QALY and utilities. *Br Med Bull* 2010;96:5-21. doi: 10.1093/bmb/ldq033 [published Online First: 2010/11/03]
22. van Dongen JM, van Wier MF, Tompa E, et al. Trial-based economic evaluations in occupational health: principles, methods, and recommendations. *J Occup Environ Med* 2014;56(6):563-72. doi: 10.1097/jom.0000000000000165 [published Online First: 2014/05/24]
23. Gray A, Clarke PM, Wolstenholme JL, et al. Applied Methods of Cost-effectiveness Analysis in Healthcare: Oxford University Press. http://econpapers.repec.org/bookchap/oxpobooks/9780199227280.htm (accessed Oct 28, 2019), 2010.
24. Herdman M, Gudex C, Lloyd A, et al. Development and preliminary testing of the new five-level version of EQ-5D (EQ-5D-5L). *Qual Life Res* 2011;20(10):1727-36. doi: 10.1007/s11136-011-9903-x [published Online First: 2011/04/12]
25. Versteegh MM, Vermeulen KM, Evers SMAA, et al. Dutch Tariff for the Five-Level Version of EQ-5D. *Value Health* 2016;19(4):343-52. doi: 10.1016/j.jval.2016.01.003 [published Online First: 2016/06/22]
26. Bouwmans C, Hakkaart-van Roijen L, Koopmanschap M, et al. Manual iMTA medical cost questionnaire (iMCQ). *Rotterdam: iMTA, Erasmus Universiteit Rotterdam* 2013
27. Hakkaart - van Roijen L, van der Linden N, Bouwmans CAM, et al. Costing manual: Methodology of costing research and reference prices for economic evaluations in healthcare. In: Volksgezondheid WeS, ed. Nederland: Zorginstituut Nederland, 2016.
28. VWS. Medicijnkosten - Zorginstituut Nederland - Ministerie van Volksgezondheid, Welzijn en Sport, 2018.
29. Bouwmans C, Krol M, Severens H, et al. The iMTA Productivity Cost Questionnaire: A Standardized Instrument for Measuring and Valuing Health-Related Productivity Losses. *Value Health* 2015;18(6):753-8. doi: 10.1016/j.jval.2015.05.009 [published Online First: 2015/09/28]
30. Koopmanschap MA, Rutten FF, van Ineveld BM, et al. The friction cost method for measuring indirect costs of disease. *J Health Econ* 1995;14(2):171-89. [published Online First: 1995/05/08]
31. Krol M, Brouwer W. How to estimate productivity costs in economic evaluations. *Pharmacoeconomics* 2014;32(4):335-44. doi: 10.1007/s40273-014-0132-3 [published Online First: 2014/02/08]
32. White IR, Royston P, Wood AM. Multiple imputation using chained equations: Issues and guidance for practice. *Stat Med* 2011;30(4):377-99. doi: 10.1002/sim.4067 [published Online First: 2011/01/13]

For peer review only - http://bmjopen.bmj.com/site/about/guidelines.xhtml

33. Madley-Dowd P, Hughes R, Tilling K, et al. The proportion of missing data should not be used to guide decisions on multiple imputation. *J Clin Epidemiol* 2019;110:63-73. doi: 10.1016/j.jclinepi.2019.02.016 [published Online First: 2019/03/18]
34. Rubin DB. Multiple Imputation for Nonresponse in Surveys: John Wiley & Sons 2004.
35. Willan AR, Briggs AH, Hoch JS. Regression methods for covariate adjustment and subgroup analysis for non-censored cost-effectiveness data. *Health Econ* 2004;13(5):461-75. doi: 10.1002/hec.843 [published Online First: 2004/05/06]
36. Twisk JWR. Applied Longitudinal Data Analysis for Epidemiology: A Practical Guide: Cambridge University Press 2013.
37. Black WC. The CE plane: a graphic representation of cost-effectiveness. *Med Decis Making* 1990;10(3):212-4. doi: 10.1177/0272989x9001000308 [published Online First: 1990/07/01]
38. Fenwick E, O'Brien BJ, Briggs A. Cost-effectiveness acceptability curves--facts, fallacies and frequently asked questions. *Health Econ* 2004;13(5):405-15. doi: 10.1002/hec.903 [published Online First: 2004/05/06]
39. McCabe C, Claxton K, Culyer AJ. The NICE cost-effectiveness threshold: what it is and what that means. *Pharmacoeconomics* 2008;26(9):733-44. doi: 10.2165/00019053-200826090-00004 [published Online First: 2008/09/05]
40. Hayakawa H, Itakura A, Mitsui T, et al. Methods for myometrium closure and other factors impacting effects on cesarean section scars of the uterine segment detected by the ultrasonography. *Acta Obstet Gynecol Scand* 2006;85(4):429-34. doi: 10.1080/00016340500430436 [published Online First: 2006/04/14]
41. Abalos E, Addo V, Brocklehurst P, et al. Caesarean section surgical techniques: 3 year follow-up of the CORONIS fractional, factorial, unmasked, randomised controlled trial. *Lancet* 2016;388(10039):62-72. doi: 10.1016/s0140-6736(16)00204-x [published Online First: 2016/05/09]
42. The CAESAR study collaborative group. Caesarean section surgical techniques: a randomised factorial trial (CAESAR). *BJOG* 2010;117(11):1366-76. doi: 10.1111/j.1471-0528.2010.02686.x [published Online First: 2010/09/16]
43. Dodd JM, Anderson ER, Gates S, et al. Surgical techniques for uterine incision and uterine closure at the time of caesarean section. *Cochrane Database Syst Rev* 2014(7):CD004732. doi: 10.1002/14651858.CD004732.pub3 [published Online First: 2014/07/23]
44. Abalos E, Addo V, Brocklehurst P, et al. Caesarean section surgical techniques (CORONIS): a fractional, factorial, unmasked, randomised controlled trial. *Lancet* 2013;382(9888):234-48. doi: 10.1016/s0140-6736(13)60441-9 [published Online First: 2013/06/01]
45. Versteegh M, Knies S, Brouwer W. From Good to Better: New Dutch Guidelines for Economic Evaluations in Healthcare. *Pharmacoeconomics* 2016;34(11):1071-74. doi: 10.1007/s40273-016-0431-y [published Online First: 2016/10/22]
46. Drummond M, Barbieri M, Cook J, et al. Transferability of economic evaluations across jurisdictions: ISPOR Good Research Practices Task Force report. *Value Health* 2009;12(4):409-18. doi: 10.1111/j.1524-4733.2008.00489.x [published Online First: 2009/11/11]
47. Perined. Perinatale zorg in Nederland anno 2018: landelijke perinatale cijfers en duiding, 2019.
48. Vikhareva O, Rickle GS, Lavesson T, et al. Hysterotomy level at Cesarean section and occurrence of large scar defects: a randomized single-blind trial. *Ultrasound Obstet Gynecol* 2019;53(4):438-42. doi: 10.1002/uog.20184 [published Online First: 2018/11/30]

For peer review only - http://bmjopen.bmj.com/site/about/guidelines.xhtml

Figures

Figure 1: Trial profile

*Logistical reasons, computer randomisation issues, passing through the allocated method to operating gynaecologist, or participant not traceable after randomisation.

Figure 2: Cost-effectiveness planes and cost acceptability curves from a societal and healthcare perspective comparing double- to single-layer uterine closure

[1] Cost-effectiveness plane (CE-plane) showing the incremental cost-effectiveness ratio point estimate (ICER, red dot) and the distribution of the 5000 replications of the bootstrapped cost-effective pairs (blue dots). [2] Cost-acceptability curve (CEAC) indicating the probability of the double-layer uterine closure being cost-effective compared to the single layer (y-axis) for different willingness-to-pay (WTP) thresholds per unit of effect gained (x-axis).
[1A] CE-plane for spotting days from a societal perspective showing that most of bootstrapped cost-effect pairs were equally distributed across CE-plane quadrants representing high uncertainty around ICER.
[2A] CEAC for spotting days from a societal perspective indicating a steady 0.2 probability of the double-layer uterine closure being cost-effective compared to the single-layer for different WTP thresholds per fewer spotting days.
[1B] CE-plane for QALYs from a societal perspective showing that most of the bootstrapped cost-effect pairs were in the north quadrants (i.e., higher costs) and western quadrants where the double-layer uterine closure was less effective compared to the single-layer.
[2B] CEAC for QALYs from a societal perspective indicating a probability of the double-layer uterine closure being cost-effective around 0.2 for different WTP thresholds per QALY gained.
[1C] CE-plane for spotting days from a healthcare perspective showing that most of the bootstrapped cost-effect pairs were in south quadrants, where the double-layer uterine closure was less costly compared to the single-layer, but they are equally distributed across the east and western quadrants representing high uncertainty around the effectiveness of the double-layer uterine closure compared to the single-closure.
[2C] CEAC for spotting days from a healthcare perspective indicating a steady 0.6 probability of the double-layer uterine closure being cost-effective compared to the single layer for different WTP thresholds per fewer spotting days.
[1D] CE-plane for QALYs from a healthcare perspective showing that most of the bootstrapped cost-effect pairs were in the south quadrants (i.e., lower costs) and western quadrants where the double-layer uterine closure was less effective compared to the single-layer.
[2D] CEAC for QALYs from a healthcare perspective indicating that the probability of the double-layer uterine closure being cost-effective compared to the single-layer decreased with an increasing of the different WTP thresholds per QALY gained because healthcare costs were on average lower in the intervention group while it is less effective compared to the usual practice.

For peer review only - http://bmjopen.bmj.com/site/about/guidelines.xhtml

Tables

Table 1: Baseline characteristics of women without amenorrhoea in the control group and intervention group.

	Single-layer (N=813)*	Double-layer (N=807)†
Age, years	32.1 (4.7)	32.0 (4.6)
Level of education		
Low	50 (6.5)	54 (7.1)
Middle	263 (34.2)	242 (31.8)
High	452 (58.8)	457 (60.0)
Nulliparous women	568 (73.9)	578 (75.9)
BMI (kg/m^2)	26.4 (4.5)	26.7 (4.9)
Smoking habit	44 (5.7)	37 (4.9)
Hypertension	146 (19.0)	127 (16.7)
Diabetes mellitus	89 (11.6)	66 (8.7)
Gestational age	38.6 (2.4)	38.6 (2.3)
Previous miscarriage	255 (33.2)	221 (29.0)
Previous ectopic pregnancy	10 (1.3)	12 (1.6)
Planned CS	504 (62.0)	503 (62.3)

Data are mean (SD), or n (%). N is equal to the total number of patients in the group. BMI, body mass index; CS, caesarean section. *5.5% missing data for all variables, except 'planned CS' (0%). †5.9% missing data for all variables, except 'planned CS' (0%).

For peer review only - http://bmjopen.bmj.com/site/about/guidelines.xhtml

Table 2: Multiply imputed mean effects and costs by group and mean difference at nine months follow-up in women without amenorrhoea

	Single-layer (n=813)	Double-layer (n=807)	Mean difference* (95% CI)
**Effects**			
Spotting days	1.44 (0.11)	1.39 (0.11)	-0.056 (-0.374 to 0.263)
QALYs gained	0.663 (0.003)	0.658 (0.004)	-0.005 (-0.015 to 0.005)
**Costs**			
Intervention costs†	0	76 (0.31)	76 (75 to 76)
Primary care costs	255 (16)	250 (17)	-5 (-49 to 40)
Secondary care costs	400 (75)	317 (44)	-83 (-292 to 38)
Medication costs	89 (84)	84 (23)	-5 (-103 to 70)
Total healthcare costs‡	744 (112)	727 (58)	-17 (-273 to 143)
Informal care costs	77 (18)	124 (33)	47 (-10 to 141)
Absenteeism costs at paid work	1052 (122)	1009 (110)	-42 (-34 to 261)
Absenteeism costs at unpaid work	3525 (226)	3810 (263)	284 (-360 to 964)
Presenteeism costs	290 (26)	256 (24)	-34 (-98 to 28)
Total lost productivity costs	4857 (280)	5076 (299)	208 (-574 to 999)
**Total societal costs§**	5689 (321)	5927 (324)	238 (-624 to 1108)

Data are mean (SE). CI=confidence interval, QALY=quality-adjusted life-years. Multiple imputation model consisted of age, education level, parity, body mass index, smoking habit, hypertensive disorder, diabetic status, gestational complications, gestational age, previous miscarriage or ectopic pregnancies, use of contraception, breastfeeding, and self-reported menstrual blood loss. *Cost and effect differences at nine months follow-up were estimated using seemingly unrelated regression analyses.[35] †Additional intervention costs to perform double-layer, excluding the average costs for performing a caesarean section (€5360,-). ‡The sum of intervention, primary care, secondary care and medication costs. §The sum of total healthcare costs, informal care costs, and lost productivity costs.

Primary care: costs of visits to general practitioners, health professionals, and complementary healthcare providers. Secondary care: costs of ambulatory hospital visits, visits to other healthcare organizations and hospital admissions. Medication costs: costs of medication use after discharge from the hospital. Informal care costs: costs of received care from family and/or friends due to health problems. Absenteeism costs at paid work: costs of sickness absenteeism from paid work. Absenteeism costs at unpaid work: costs of absenteeism from unpaid work activities (e.g., household tasks, childcare, voluntary work). Presenteeism costs: costs of working while suffering from health complaints.

For peer review only

For peer review only - http://bmjopen.bmj.com/site/about/guidelines.xhtml

Table 3: Results of the cost-effectiveness analysis

Effect outcome*	Cost difference, € (95% CI)	Effect difference (95% CI)	ICER €/ effect gained	Distribution of the cost-effectiveness plane			
				North-East	South-East	South-West	North-West
**Main analysis – Societal perspective**							
Spotting days	238 (-624 to 1108)	0.056 (-0.263 to 0.374)	4281	44%	20%	10%	26%
QALY	238 (-624 to 1108)	-0.005 (-0.015 to 0.005)	-49699	8%	9%	21%	62%
**Main analysis – Healthcare perspective**							
Spotting days	-17 (-283 to 146)	0.056 (-0.263 to 0.374)	-311	30%	34%	18%	18%
QALY	-17 (-283 to 146)	-0.005 (-0.015 to 0.005)	3614	6%	10%	42%	42%
**SA1 – including all women randomised - Societal perspective**							
QALY	150 (-764 to 944)	-0.006 (-0.014 to 0.002)	-25696	4%	6%	30%	60%
**SA1 – including all women randomised - Healthcare perspective**							
QALY	-235 (-1230 to 84)	-0.005 (-0.014 to 0.004)	46765	3%	10%	66%	21%
**SA2 – complete case analysis – Societal perspective**							
Spotting days	346 (-641 to 1394)	0.149 (-0.452 to 0.138)	2324	69%	18%	3%	10%
QALY	313 (-671 to 1382)	0.006 (-0.016 to 0.003)	50787	73%	17%	5%	5%
**SA2 - complete case analysis – Healthcare perspective**							
Spotting days	-9 (-251 to 174)	0.186 (-0.486 to 0.103)	-46	54%	37%	4%	5%
QALY	1 (-256 to 172)	0.007 (-0.016 to 0.002)	80	59%	34%	4%	3%
**SA3 – per protocol analysis - Societal perspective**							
Spotting days	43 (-820 to 903)	0.043 (-0.274 to 0.361)	1008	32%	29%	17%	22%
QALY	43 (-820 to 903)	0.004 (-0.006 to 0.013)	11909	47%	31%	15%	7%
**SA3 – per protocol analysis – Healthcare perspective**							
Spotting days	-39 (-301 to 126)	0.043 (-0.2748 to 0.361)	-909	23%	38%	23%	16%
QALY	-39 (-301 to 126)	0.004 (-0.006 to 0.013)	-10745	32%	45%	16%	7%
**SA4 – Main analysis adjusted – Societal Perspective**							
Spotting days	226 (-633 to 1092)	0.046 (-0.277 to 0.369)	4961	41%	19%	12%	28%
QALY	226 (-633 to 1092)	-0.004 (-0.014 to 0.006)	-58497	10%	11%	20%	59%
**SA4 – Main analysis adjusted – Healthcare perspective**							
Spotting days	-11 (-267 to 154)	0.046 (-0.277 to 0.369)	-248	29%	31%	19%	21%
QALY	-11 (-267 to 154)	-0.004 (-0.014 to 0.006)	2925	9%	13%	37%	41%

Data are mean (95% CI). *The effect outcome “Spotting days” was multiplied by -1 in the cost-effectiveness analysis to keep the CE-plane interpretable. CI=confidence interval, ICER=incremental cost-effectiveness ratio, QALY=quality-adjusted life-year, SA=sensitivity analysis.

Main analysis: CEA from a societal and a healthcare perspective for spotting days and QALY including only women without amenorrhoea (total=1620, control n=813, intervention n=807).

SA1: CEA from a societal and a healthcare perspective for QALY, including all women randomised in the study after multiple imputation (i.e., without excluding amenorrhoeic women, n=2292)[Supplementary Table S3].

SA2: CEA from a societal perspective using complete cases for spotting days and total societal costs (total=1065, control n=544, intervention n=521) including only women without amenorrhoea (n=1620).

SA2: CEA from a societal perspective using complete cases for QALY and total societal costs (total=1057, control n=541, intervention n=516) including only women without amenorrhoea (n=1620).

SA2: CEA from a healthcare perspective using complete cases for spotting days and total healthcare costs (total=1315, control n=662, intervention n=653) including only women without amenorrhoea (n=1620).

SA2: CEA from a healthcare perspective using complete cases for QALY and total healthcare costs (total=1310, control n=657, intervention n=653) including only women without amenorrhoea (n=1620).

SA3: per-protocol analysis for spotting days and QALY from a societal perspective (total=1620, control n=828, intervention n=792) including only women without amenorrhoea (n=1620).

SA3: per-protocol analysis for spotting days and QALY from a healthcare perspective (total=1620, control n=828, intervention n=792) including only women without amenorrhoea (n=1620).

SA4: main analysis adjusted for the use of contraception and breastfeeding during follow-up from a societal and a healthcare perspective (total=1620, control n=813, intervention n=807).

For peer review only - http://bmjopen.bmj.com/site/about/guidelines.xhtml

For peer review only

For peer review only - http://bmjopen.bmj.com/site/about/guidelines.xhtml

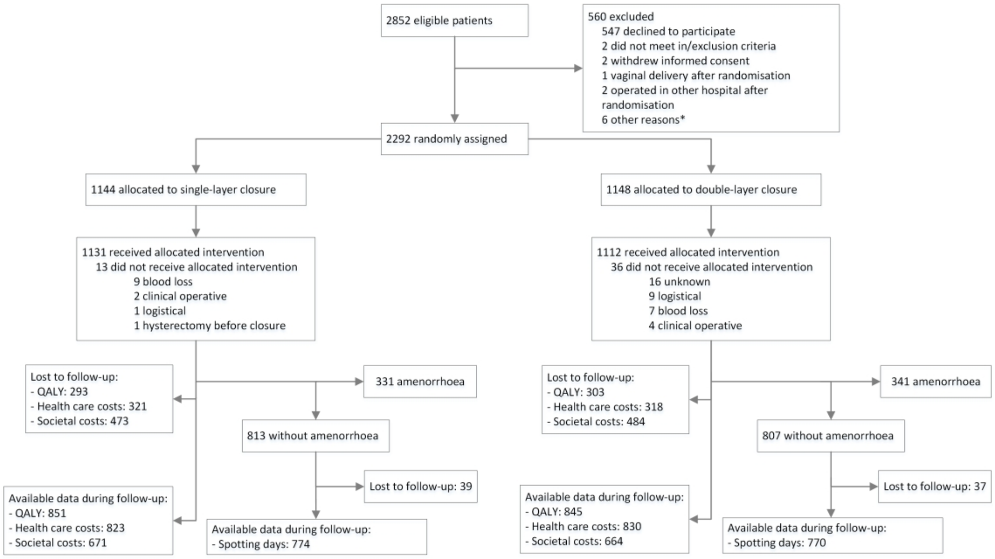

Figure 1: Trial profile

*Logistical reasons, computer randomisation issues, passing through the allocated method to operating gynaecologist, or participant not traceable after randomisation.

287x161mm (300 x 300 DPI)

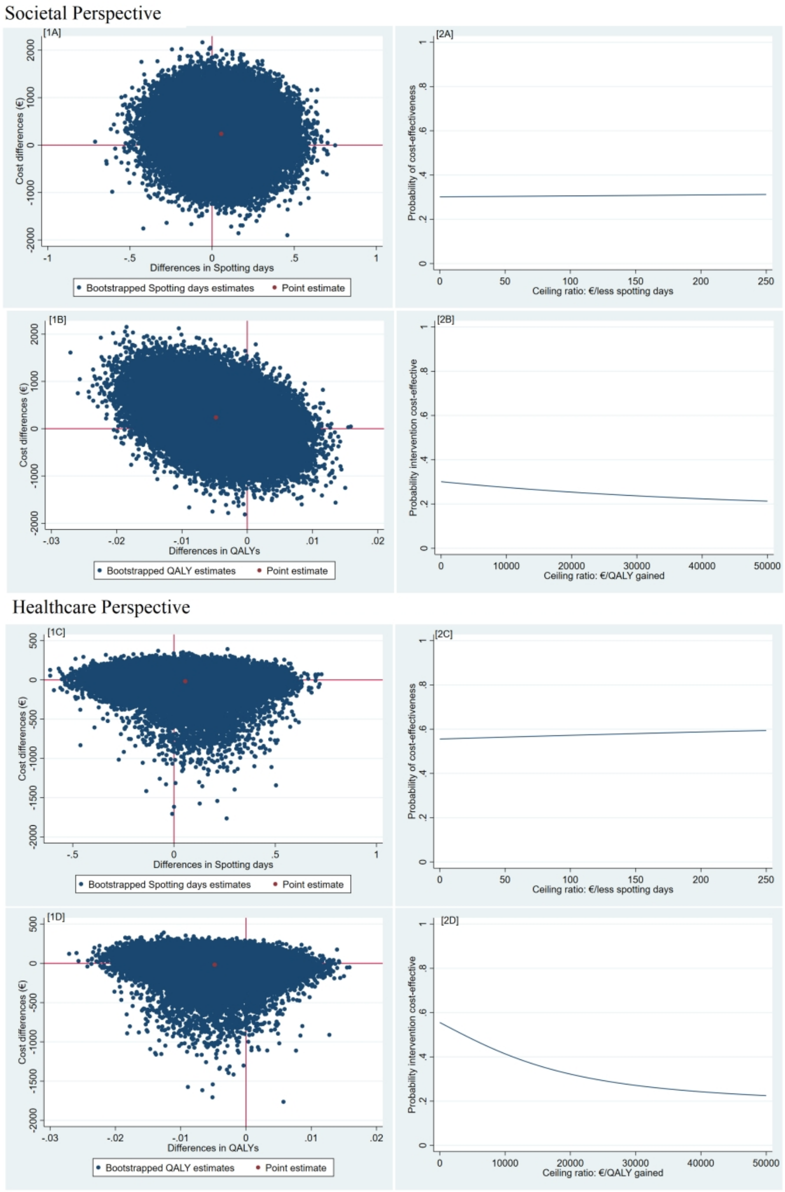

Figure 2: Cost-effectiveness planes and cost acceptability curves from a societal and healthcare perspective comparing double- to single-layer uterine closure

[1] Cost-effectiveness plane (CE-plane) showing the incremental cost-effectiveness ratio point estimate (ICER, red dot) and the distribution of the 5000 replications of the bootstrapped cost-effective pairs (blue dots). [2] Cost-acceptability curve (CEAC) indicating the probability of the double-layer uterine closure being cost-effective compared to the single layer (y-axis) for different willingness-to-pay (WTP) thresholds per unit of effect gained (x-axis).
[1A] CE-plane for spotting days from a societal perspective showing that most of bootstrapped cost-effect pairs were equally distributed across CE-plane quadrants representing high uncertainty around ICER.
[2A] CEAC for spotting days from a societal perspective indicating a steady 0.2 probability of the double-layer uterine closure being cost-effective compared to the single-layer for different WTP thresholds per fewer spotting days.
[1B] CE-plane for QALYs from a societal perspective showing that most of the bootstrapped cost-effect pairs

were in the north quadrants (i.e., higher costs) and western quadrants where the double-layer uterine closure was less effective compared to the single-layer.
[2B] CEAC for QALYs from a societal perspective indicating a probability of the double-layer uterine closure being cost-effective around 0.2 for different WTP thresholds per QALY gained.
[1C] CE-plane for spotting days from a healthcare perspective showing that most of the bootstrapped cost-effect pairs were in south quadrants, where the double-layer uterine closure was less costly compared to the single-layer, but they are equally distributed across the east and western quadrants representing high uncertainty around the effectiveness of the double-layer uterine closure compared to the single-closure.
[2C] CEAC for spotting days from a healthcare perspective indicating a steady 0.6 probability of the double-layer uterine closure being cost-effective compared to the single layer for different WTP thresholds per fewer spotting days.
[1D] CE-plane for QALYs from a healthcare perspective showing that most of the bootstrapped cost-effect pairs were in the south quadrants (i.e., lower costs) and western quadrants where the double-layer uterine closure was less effective compared to the single-layer.
[2D] CEAC for QALYs from a healthcare perspective indicating that the probability of the double-layer uterine closure being cost-effective compared to the single-layer decreased with an increasing of the different WTP thresholds per QALY gained because healthcare costs were on average lower in the intervention group while it is less effective compared to the usual practice.

Supplementary appendix

Appendix S1. List of collaborators.

Hospital	Site principal investigator
Amphia hospital, Breda	Dimitri NM Papatsonis
Amsterdam UMC, Univ of Amsterdam, Amsterdam	Eva Pajkrt
Amsterdam UMC, VU University, Amsterdam	Wouter JK Hehenkamp
Bernhoven hospital, Uden	Angèle LM Oei
Birth Centre Wilhelmina Children Hospital/University Medical Centre Utrecht, Utrecht	Mireille N Bekker
Canisius-Wilhelmina hospital, Nijmegen	Daniela H Schippers
Catharina hospital, Eindhoven	Huib AAM van Vliet
Deventer ziekenhuis, Deventer	Lucet van der Voet
Diakonessenhuis, Utrecht	Nico WE Schuitemaker
Dijklander hospital – location Hoorn	Majoie Hemelaar
Flevo hospital, Almere	WM (Marchien) van Baal
Gelre hospital – location Apeldoorn	Anjoke JM Huisjes
Gelre hospital – location Zutphen	Wouter J Meijer
Groene Hart hospital, Gouda	CAH (Ineke) Janssen
Haaglanden Medical Centre – Westeinde hospital, Den Haag	Wietske Hermes
Haga hospital, Den Haag	AH (Hanneke) Feitsma
Isala clinics, Zwolle	Hugo WF van Eijndhoven
Jeroen Bosch hospital, 's-Hertogenbosch	Robbert JP Rijnders
Leiden University Medical Centre, Leiden	Marieke Sueters
Maastricht University Medical Centre, Research school 'GROW', Maastricht	HCJ (Liesbeth) Scheepers
Máxima Medical Centre, Veldhoven	Judith OEH van Laar
Meander Medical Centre, Amersfoort	Elisabeth MA Boormans
OLVG-oost, Amsterdam	Paul JM van Kesteren
OLVG-west, Amsterdam	Celine M Radder
Radboud University Nijmegen Medical Centre, Nijmegen	Esther Hink
Reinier de Graaf hospital, Delft	Kitty Kapiteijn
Rijnstate hospital, Arnhem	Karin de Boer
Röpcke-Zweers hospital, Hardenberg	Mesrure Kaplan
Sint Antonius Hospital, Nieuwegein	Erik van Beek
Sint Franciscus Hospital, Rotterdam	LHM (Marloes) de Vleeschouwer
Tergooi hospital, Blaricum	Harry Visser
Zuyderland Medical Centre, Heerlen	Josje Langenveld

For peer review only

Supplementary tables

Table S1: Costs of the intervention per participant

Resources	Units	Unit price, €	Costs, €
Additional suture material	One piece	6.14	6.14
Additional operation time, academic hospital (3.9 minutes)	Hour	1379.27	89.65
Total costs per participant in academic hospital			95.79
Additional operation time, non-academic hospital (3.9 minutes)	Hour	1000	65.00
Total costs per participant in non-academic hospital			71.14

Table S2: Probability of the intervention being cost-effective at different willingness-to-pay thresholds for spotting days

	Main analyses – spotting days	
WTP €/ unit of effect gained	*Societal Perspective*	*Healthcare perspective*
0	0.30	0.55
31.6 (1 working hour)	0.30	0.56
126.4 (4 working hours)	0.31	0.58
252.8 (8 working hours)	0.31	0.59
	**Main analyses - QALYs**	
0	0.30	0.55
10000	0.27	0.41
20000	0.25	0.32
30000	0.24	0.27
40000	0.22	0.24
50000	0.21	0.22

WTP=willingness-to-pay; QALY=quality-adjusted life-years

Table S3: Description of follow-up complete cases and missing cases by effect and cost outcomes without excluding cases of amenorrhoea

	Complete cases			Missing cases (%)		
	Intervention	*Control*	*Total*	*Intervention*	*Control*	*Total*
Spotting days	936	940	1876	212 (23)	204 (22)	416 (22)
QALY	845	851	1696	303 (36)	293 (34)	596 (35)
Primary care costs	913	924	1837	235 (26)	220 (24)	455 (25)
Secondary care costs	838	833	1671	310 (37)	311 (37)	621 (37)
Medication costs	834	825	1659	314 (38)	319 (39)	633 (38)
Total healthcare costs	830	823	1653	318 (38)	321 (39)	639 (39)
Informal care costs	913	924	1837	235 (26)	220 (24)	455 (25)
Absenteeism costs at work	852	849	1701	296 (35)	295 (35)	591 (35)
Absenteeism costs at unpaid work	905	921	1826	243 (27)	223 (24)	466 (25)
Presenteeism costs	680	690	1370	468 (69)	454 (66)	922 (67)
Total lost productivity costs	677	689	1366	471 (69)	455 (66)	926 (68)
Total societal costs	664	671	1335	484 (73)	473 (70)	957 (72)

For peer review only

Stegwee et al. BMC Pregnancy and Childbirth (2019) 19:85
https://doi.org/10.1186/s12884-019-2221-y

BMC Pregnancy and Childbirth

STUDY PROTOCOL Open Access

Single- versus double-layer closure of the caesarean (uterine) scar in the prevention of gynaecological symptoms in relation to niche development – the 2Close study: a multicentre randomised controlled trial

S. I. Stegwee[1†], I. P. M. Jordans[1†], L. F. van der Voet[2], M. Y. Bongers[3,19], C. J. M. de Groot[1], C. B. Lambalk[1], R. A. de Leeuw[1], W. J. K. Hehenkamp[1], P. M. van de Ven[4], J. E. Bosmans[5], E. Pajkrt[6], E. A. Bakkum[7], C. M. Radder[8], M. Hemelaar[9], W. M. van Baal[10], H. Visser[11], J. O. E. H. van Laar[3], H. A. A. M. van Vliet[12], R. J. P. Rijnders[13], M. Sueters[14], C. A. H. Janssen[15], W. Hermes[16], A. H. Feitsma[17], K. Kapiteijn[18], H. C. J. Scheepers[19], J. Langenveld[20], K. de Boer[21], S. F. P. J. Coppus[22], D. H. Schippers[23], A. L. M. Oei[24], M. Kaplan[25], D. N. M. Papatsonis[26], L. H. M. de Vleeschouwer[27], E. van Beek[28], M. N. Bekker[29], A. J. M. Huisjes[30], W. J. Meijer[31], K. L. Deurloo[32], E. M. A. Boormans[33], H. W. F. van Eijndhoven[34] and J. A. F. Huirne[1*]

Abstract

Background: Double-layer compared to single-layer closure of the uterus after a caesarean section (CS) leads to a thicker myometrial layer at the site of the CS scar, also called residual myometrium thickness (RMT). It possibly decreases the development of a niche, which is an interruption of the myometrium at the site of the uterine scar. Thin RMT and a niche are associated with gynaecological symptoms, obstetric complications in a subsequent pregnancy and delivery and possibly with subfertility.

(Continued on next page)

* Correspondence: j.huirne@vumc.nl
†S. I. Stegwee and I. P. M. Jordans contributed equally to this work.
[1]Department of Obstetrics and Gynaecology, Research institutes 'Amsterdam Cardiovascular Sciences' and 'Amsterdam Reproduction and Development', Amsterdam UMC, Vrije Universiteit Amsterdam, De Boelelaan 1117, 1081 HV Amsterdam, Netherlands
Full list of author information is available at the end of the article

© The Author(s). 2019 **Open Access** This article is distributed under the terms of the Creative Commons Attribution 4.0 International License (http://creativecommons.org/licenses/by/4.0/), which permits unrestricted use, distribution, and reproduction in any medium, provided you give appropriate credit to the original author(s) and the source, provide a link to the Creative Commons license, and indicate if changes were made. The Creative Commons Public Domain Dedication waiver (http://creativecommons.org/publicdomain/zero/1.0/) applies to the data made available in this article, unless otherwise stated.

For peer review only - http://bmjopen.bmj.com/site/about/guidelines.xhtml

(Continued from previous page)

Methods: Women undergoing a first CS regardless of the gestational age will be asked to participate in this multicentre, double blinded randomised controlled trial (RCT). They will be randomised to single-layer closure or double-layer closure of the uterine incision. Single-layer closure (control group) is performed with a continuous running, unlocked suture, with or without endometrial saving technique. Double-layer closure (intervention group) is performed with the first layer in a continuous unlocked suture including the endometrial layer and the second layer is also continuous unlocked and imbricates the first. The primary outcome is the reported number of days with postmenstrual spotting during one menstrual cycle nine months after CS. Secondary outcomes include surgical data, ultrasound evaluation at three months, menstrual pattern, dysmenorrhea, quality of life, and sexual function at nine months. Structured transvaginal ultrasound (TVUS) evaluation is performed to assess the uterine scar and if necessary saline infusion sonohysterography (SIS) or gel instillation sonohysterography (GIS) will be added to the examination. Women and ultrasound examiners will be blinded for allocation. Reproductive outcomes at three years follow-up including fertility, mode of delivery and complications in subsequent deliveries will be studied as well. Analyses will be performed by intention to treat. 2290 women have to be randomised to show a reduction of 15% in the mean number of spotting days. Additionally, a cost-effectiveness analysis will be performed from a societal perspective.

Discussion: This RCT will provide insight in the outcomes of single- compared to double-layer closure technique after CS, including postmenstrual spotting and subfertility in relation to niche development measured by ultrasound.

Trial registration: Dutch Trial Register (NTR5480). Registered 29 October 2015.

Keywords: Caesarean section, Closure techniques, Long-term outcomes, Postmenstrual spotting, Niche, Quality of life, Fertility, Reproductive outcomes,

For peer review only

Background

Caesarean section (CS) rates have increased from 14.5 to 27.2% in the last two decades in the Western world. [1] In 2016, 26.664 CSs were performed in the Netherlands, being 16.0% of the total number of deliveries. [2] The increasing CS rate has stimulated an interest in the potential long-term morbidity of a CS scar, such as uterine rupture or malplacentation. [3–7] Other less severe, but more prevalent long-term symptoms are gynaecological symptoms and subfertility.

Only recently, gynaecological symptoms such as painful menstruations and postmenstrual spotting have been associated with CSs. [8–10] These symptoms are considered to be related to a niche, defined as *"an indentation at the site of the caesarean scar with a depth of at least 2 mm"*, visible on transvaginal ultrasound (TVUS). [11] Two cohort studies reported a strong association between postmenstrual spotting and a niche: odds ratio (OR) 3.1; 95% confidence interval (CI) 1.5–6.3 [8] and OR 5.5; 95% CI 1.1–26.5. [10] In these studies, a niche was observed in 50 to 60% of the women after a CS, using transvaginal ultrasound. [8, 10] Spotting was correlated to niche volume and inversely correlated to the residual myometrium thickness (RMT). [8, 10]

In addition to the gynaecological symptoms, a niche may influence fertility. A recent meta-analysis reported that a CS on average reduced the probability of subsequent pregnancy with 9% (relative risk (RR) 0.91; 95% CI 0.87–0.95) in comparison to a vaginal delivery. [12] None of the included studies in this meta-analysis evaluated the relation between subsequent fertility and the presence of a niche. One of the hypotheses is that intra-uterine fluid or cervical mucus or blood accumulation in the niche are expected to hamper the penetration of sperm cells or impair embryo implantation. [13] Long-term follow-up will facilitate the evaluation of the association between uterine closure, niche development, accumulation of intra-uterine fluid and subfertility.

In the last years, various therapies have been developed and implemented to treat niche related symptoms such as menstrual disorders. [14–18] Effectiveness of both hysteroscopic [19] and laparoscopic niche resection [15] have recently been published. Because both niche related symptoms and applied therapies lead to increases in medical consultations and costs, it seems to be more efficient to prevent niche development in the first place. Uterine closure technique of the CS scar has been proposed as an independent factor for niche development. [9] However, large randomised trials evaluating the effect of uterine closuring techniques on postmenstrual spotting or other gynaecological or reproductive outcomes in relation to niche development and thin residual myometrium are lacking, as well as cost-effectiveness evaluations.

In order to shorten surgery time and in the absence of significant differences in short-term outcomes [20, 21], most Dutch gynaecologists (92%) have replaced double-layer by single-layer closure after a CS, using

multifilament continuous unlocked sutures. Given the higher risk on myometrium loss and thus development of a thinner residual myometrium after single-layer closure [5, 22], we hypothesise that this method introduces a higher risk on postmenstrual spotting and possibly subfertility after a CS and that it can be prevented by applying double-layer unlocked closure.

Double-layer unlocked closure is considered safe, without a clinically relevant higher risk on short-term outcomes. [5, 23, 24]. Moreover, it results in a thicker residual myometrium, especially when unlocked sutures are applied. [5, 22, 24] Dysmenorrhea was reported more frequently after single-layer closure, but this was only studied in two RCTs and not always related to ultrasound findings such as myometrial thickness or niche presence. [24] Prevalence of uterine rupture seems to be similar after single- versus double-layer closure [5, 22, 24], but has neither been related to ultrasound findings and since it has a very low incidence, statistically significant differences are difficult to find. Since long-term outcomes such as gynaecological symptoms, fertility outcomes and results of subsequent pregnancies are studied infrequently, additional evidence is needed before a preference for either technique can be indicated.

Objective

Our primary objective is to determine the effectiveness of unlocked double-layer uterine closure compared to unlocked single-layer uterine closure in the prevention of niche related gynaecological symptoms nine months after a first CS. Secondary objectives are to assess niche prevalence measured by ultrasound at three months follow-up and to study both reproductive outcomes related to a subsequent pregnancy and gynaecological symptoms at three years follow-up. Additionally we aim to study the cost-effectiveness alongside the trial.

Methods/design

Design

This multicentre randomised controlled superiority trial will be performed in the Netherlands, in hospitals that collaborate within the Dutch Consortium for Healthcare Evaluation and Research in Obstetrics and Gynaecology (NVOG Consortium 2.0, www.zorgevaluatienederland.nl). Centres that participate are district, teaching or university hospitals in the Netherlands. A list of study sites is available in Additional file 1.

Participants and eligibility criteria

All women who undergo a first CS, planned or unplanned, will be asked to participate in the study. Other inclusion criteria are: sufficient command of the Dutch or English language, age ≥ 18 years and written informed consent. To prevent confounding effects on niche development during the study, we will exclude women with a previous CS. Other exclusion criteria are: inadequate possibility for counselling (e.g. indication for emergency CS without being informed about the study previously, women in severe pain without adequate therapy), previous major uterine surgery (e.g. laparoscopic or laparotomic fibroid resection, septum resection), women with known causes of menstrual disorders (e.g. cervical dysplasia, communicating hydrosalpinx, uterine anomaly or endocrine disorders disturbing ovulation), placenta increta or percreta during the current pregnancy or ≥ three foetuses during the current pregnancy.

Recruitment and randomisation

Eligible women will be asked by a gynaecologist, resident, clinical midwife or research nurse to participate in the trial when they undergo a planned CS. Eligible women who are planned to undergo a vaginal delivery will also be informed about this study during pregnancy in case they need an unplanned CS. Furthermore, women during induced labour and women receiving adequate therapy for pain during labour, will be asked to participate in case a CS is needed during labour for any indication.

When the decision of a CS is made and all selection criteria are met, women will be randomly allocated to single-layer (control group) or double-layer (intervention group) closure (1:1) (see Fig. 1). Randomisation will be performed using a web-based application ALEA 2.2 which displays a computer-generated random number, managed by the Clinical Research Unit of the Amsterdam UMC - location AMC. We will use a permuted block-design, stratified for recruiting centres and for planned or unplanned CS. All women that decline to participate will be registered anonymously in order to record the number and reason for refusal. Subjects who withdraw from this study will not be replaced.

Gynaecologists, residents, clinical midwives or research nurses enrol participants and assign them to the intervention. The CS will be performed by either a gynaecologist, a resident supervised by a gynaecologist or by a resident that is authorised to perform CSs without supervision. Participants and sonographers will be blinded for the closure technique. If operative reintervention after CS is needed and the gynaecologist that performs the reintervention needs to know the closure technique that the participant was assigned to, unblinding is possible through the logistic trial coordinator. We expect this situation to occur very infrequently.

Intervention (double-layer closure)

In both study arms, women will undergo a CS following a standard way with respect to mode of uterotomy,

correct approximations of the cutting edges and non-closure of the peritoneum. In the intervention arm, double-layer closure of the uterus will be performed using unlocked multifilament continuous running sutures for both layers and the endometrial layer will be included in the first layer (see Fig. 2). The second layer is a continuous running suture that imbricates the first layer. Since this is not the standard method for uterine closure in the Netherlands, a short online instruction film will be shown to all participating centres and surgeons prior to participation (see Additional file 2). Surgical outcomes will be registered after the procedure in the electronic case report form (eCRF).

Control group (single-layer closure)

The control group will receive usual closure technique of the uterus: a single-layer closure using unlocked continuous running multifilament sutures. The currently available evidence is inconclusive with respect to endometrial saving technique or not. Therefore, we decided that in our study surgeons are free to choose to close either full thickness (including the endometrium) or split

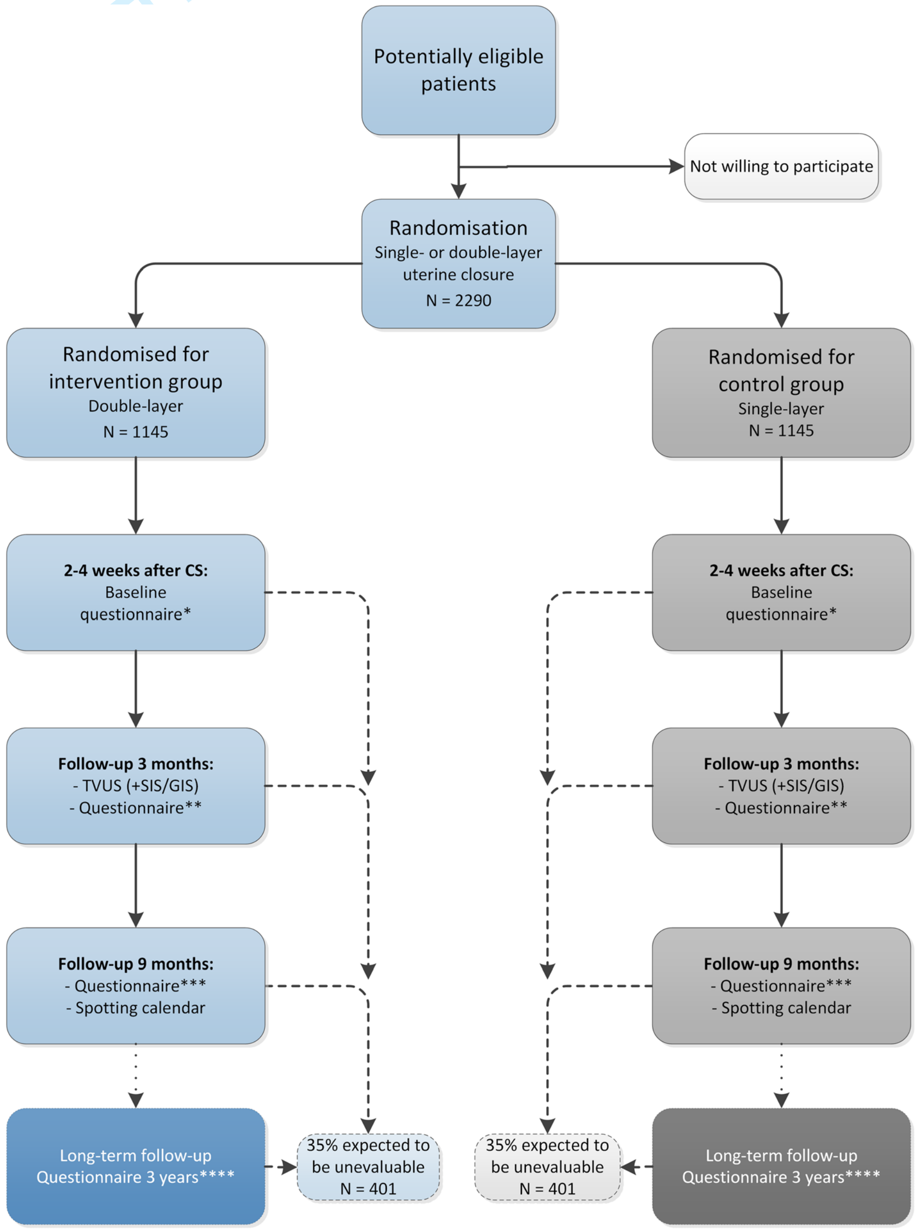

Fig. 1 Flowchart of the 2Close study. * = baseline questions, EQ-5D-5L. ** = symptom questionnaire, EQ-5D-5L, SF36, PROMIS SF8a, iMCQ, iPCQ. *** = symptom questionnaire, FSFI, EQ-5D-5L, SF36, iMCQ, iPCQ. **** = symptom questionnaire, fertility questionnaire, FSFI, EQ-5D-5L, SF36, PROMIS SF8a

thickness (excluding the endometrium) in the control group. The applied method, including endometrial saving technique or not, will be registered.

Niche evaluation

The care after CS will be according to the normal local protocol with the regular outpatient visit that is normally executed six weeks after the CS. This routine visit may be postponed to three months after the CS to enable an ultrasound evaluation to identify the existence of a niche, but participating centres may decide whether they want visits at six weeks (routine follow-up) and at three months (ultrasound follow-up) or only one visit after three months combining the regular control and the ultrasound follow-up. The ultrasound evaluation is standardised as proposed by Jordans et al. [11] (see Fig. 3). Based on this standardisation, we created an obligatory e-learning for all ultrasound performers to let all ultrasounds be performed in a uniform manner. To increase consistency and to improve the learning curve, we will evaluate a sample of ultrasounds in each centre based on recorded pictures and provide feedback to the examiners. Since it is known that a niche can be missed during TVUS only [8, 10, 25] we will additionally perform a saline infusion sonohysterography (SIS) or gel instillation sonography (GIS) in case no niche is observed during the normal TVUS or if the ultrasound is inconclusive. It would be optimal to have a contrast enhanced ultrasound in all women when the uterine cavity or niche are not naturally filled with fluid, but we have chosen for this approach to prevent unnecessary burden for the participants and to reduce costs.

Outcome measures

Primary outcome measure

The primary outcome is the number of days of postmenstrual spotting during one cycle at nine months after CS. We defined postmenstrual spotting as brownish discharge for more than two days at the end of the menstruation, with a total duration (menstruation and spotting) of more than seven days, or intermenstrual blood loss that starts after the end of the menstruation. [8] The number of days of postmenstrual spotting will be counted as follows: days with brownish discharge (> two days) when the total duration of menstruation and spotting exceeds seven days + number of days with

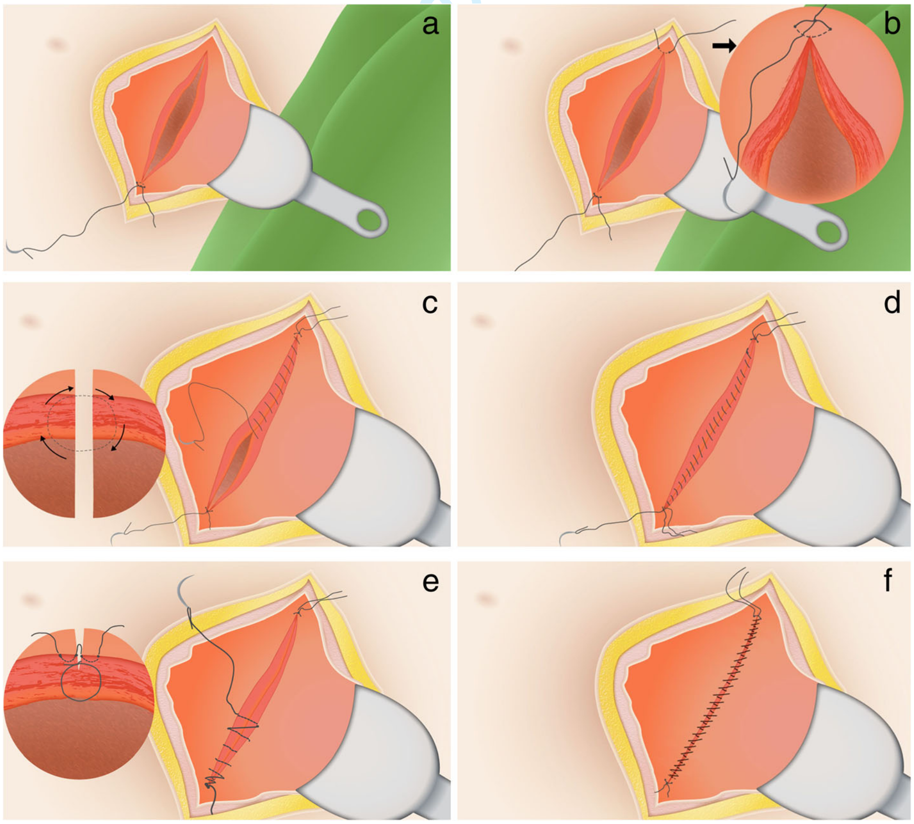

Fig. 2 Double-layer uterine closure technique. **a**. Step 1: lateral suture; **b**. Step 2: lateral suture on the other side; **c**. Step 3: First layer: full thickness, continuous, including large part of myometrium, including the endometrial layer; **d**. Step 4: End of this first layer; **e**. Step 5: Second layer: superficial continuous layer of serosal tissue, imbricating the first layer; **f**. Step 6: First and second layer should be closely connected

intermenstrual blood loss. Amenorrhoeic women, due to lactation, medication or other diseases, will not be evaluable for the primary outcome and will be left out of this analysis.

Secondary outcome measures at short-term

- Perioperative outcomes including blood loss, operative time, additional haemostatic sutures and complications.
- Menstruation characteristics, dysmenorrhea (visual analogue scale (VAS)), Quality of Life (QOL) using Short-Form-36 [26] and EQ-5D-5L [27, 28], societal reintegration (PROMIS Short-Form-8a [29]), sexual function using the Female Sexual Function Index (FSFI [30]), applied medical and/or surgical therapy because of gynaecological symptoms, all obtained through digital questionnaires, will be assessed at three and nine months follow-up.
- Ultrasound evaluation will be performed at three months follow-up using TVUS, in which RMT, adjacent myometrium thickness (AMT), presence of a niche (depth of ≥2mm), length, depth and width of the niche, presence of large niches (RMT < 50% of AMT, RMT <3mm) and niche volume will be measured.

Cost-effectiveness outcomes

Costs will be measured using adapted versions of the iMTA Productivity Cost Questionnaire (iPCQ [31]) and iMTA Medical Consumption Questionnaire (iMCQ [32]) from a health care and societal perspective at nine months of follow-up.

Secondary outcome measures at long-term

- Menstruation characteristics, pain, sexual functioning, QOL and social reintegration will be evaluated at three years follow-up.
- Reproductive outcomes at three years follow-up: % of women desiring to conceive, % of women that conceived including time to conceive, % of women with an ongoing pregnancy, the need for fertility treatment and pregnancy outcomes such as mode of delivery or complications will be determined.

Long-term outcomes will be presented in a separate article.

Data collection and data management

Intraoperative data

Immediately after the CS we will register relevant items regarding the delivery and CS in an eCRF, in which confidentiality and anonymity are ensured and audit trails are accessible. These items include: reason for planned or unplanned CS, emergency CS or not, whether women experienced contractions, dilatation, performed method for uterine closure, endometrial saving technique applied, used suturing material, extra haemostatic sutures, operative time, blood loss and complications.

Collection of baseline characteristics and patient reported outcomes

Baseline characteristics will be collected through a digital questionnaire at 2–4 weeks after caesarean section, sent to the e-mail address of participants. Since we will also include unplanned CSs, we decided that it is not possible for all participants to answer questions regarding baseline characteristics before the operation. Baseline parameters include maternal age, body mass index, social economic status, smoking habit, medical and obstetric history, gestational age and previous vaginal deliveries, all reported by the participant. We expect that the impact of niche related symptoms such as postmenstrual spotting on daily activities and sexual behaviour may be influenced by ethnic background and religion, therefore we will also register these characteristics. At three months, nine months and three years follow-up, again digital questionnaires will be sent to participants to assess the primary and secondary outcomes (see Fig. 1). At nine months, we ask participants record their exact menstrual and spotting pattern, if any, in an adjusted menstruation score chart. [33] Reminders for all questionnaires will be sent every two weeks, with a maximum of three times. When no response is given after the reminders, research nurses from participating centres will be asked to call the participant.

Data niche evaluation

Results of the TVUS and GIS or SIS, performed three months after CS, will be registered. Women will not receive information regarding the presence of a niche, since it has no clinical consequences so shortly after CS and this may influence the answers given in the questionnaires. Other important abnormalities visualised by ultrasound will be reported as usual.

Statistical issues

Sample size calculation

We use a superiority design since we expect double-layer closure to be favourable. Literature for making reliable estimations on postmenstrual spotting in relation to niches is scarce. We have used baseline data from the HysNiche [19] and LapNiche [15] study. We

For peer review only - http://bmjopen.bmj.com/site/about/guidelines.xhtml

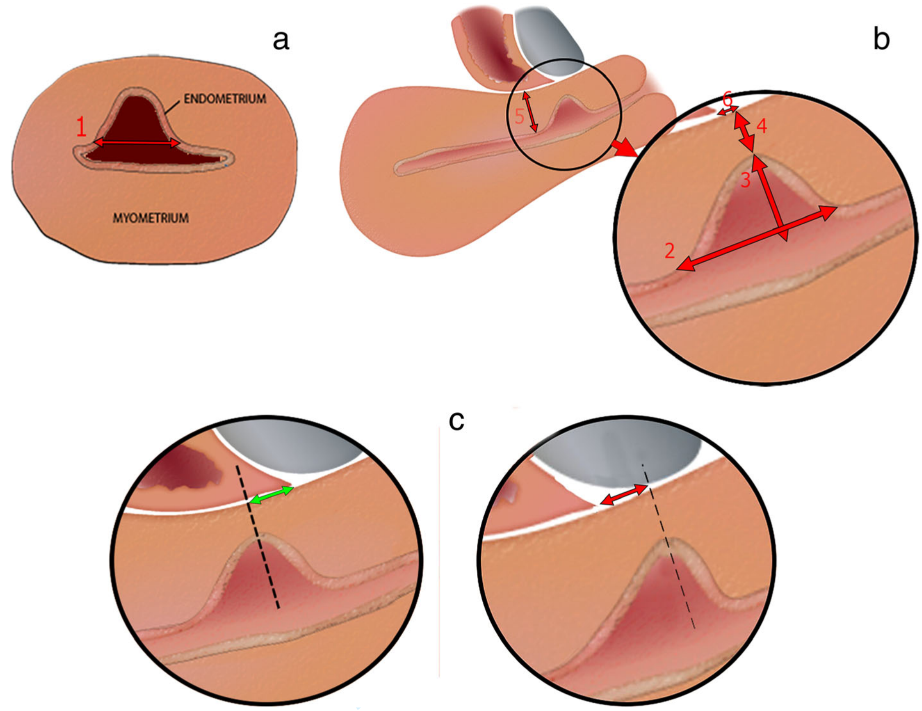

Fig. 3 Standardised transvaginal ultrasound evaluation of a niche in the non-pregnant uterus. **a**. Measured in the transversal plane: niche width (1); **b**. Measured in the sagittal plane: niche length (2), niche depth (from cervical canal until apex of the niche) (3), residual myometrium thickness (from deepest part of the niche until the serosa) (4), adjacent myometrium thickness (myometrium thickness close to base of the niche) (5), distance from apex of the niche to vesico-vaginal fold (see **c**) (6); **c**. Measuring the niche relatively to the vesico-vaginal fold in the sagittal plane: positive value (green arrow, in mm) or negative value (red arrow, in mm)

estimate the mean number of spotting days to be 3.5 days/month in the total group. We consider a 15% reduction in the mean number of spotting days clinically relevant, which is 0.5 day/month reduction. Assuming a standard deviation (SD) of 3.4 and a two-sided significance level of 5%, a total of 1488 women need to be included to achieve a power of 80%. Increasing the sample size to take into account 35% of women unevaluable (due to drop-out, non-response or amenorrhoea) for the primary outcome, 2290 women need to be included.

Data-analysis

Data-analysis will be performed according to the intention to treat principle and additional per protocol analyses will be performed. A test will be considered statistically significant when the two-sided test shows a p-value < 0.05. Baseline characteristics will be presented using percentages, means with SD and 95% CI or medians with interquartile ranges (IQR), where appropriate.

The primary outcome, number of days of postmenstrual spotting, will be presented for both groups as mean with SD or median with IQR, and presented in a Box-Whisker graph to show the distribution. Differences in primary outcome between the groups will be tested using the independent t-test in case of normal distribution (possible after transformation of the outcome) or Mann-Whitney U test. An adjusted analysis will be performed using linear regression analysis in which we adjust for factors on which randomisation was stratified and for baseline factors on which relevant differences are observed despite randomisation.

Dichotomous secondary outcomes will be presented as percentages and RR with corresponding 95% CI. P-values will be calculated using the chi-square test or, if the expected count for at least one cell is below 5, using the Fisher exact test. Normally distributed continuous variables will be presented as means with SD, and differences between the groups will be calculated with an independent t-test. Non-normally distributed continuous variables will be presented as medians with IQR and differences between the groups will be calculated with Mann-Whitney U test. The questionnaires will be analysed using the appropriate algorithms and usual presentation methods (FSFI, EQ-5D-5L, SF36, PROMIS SF8a, iMCQ, iPCQ).

Comparison of primary outcome between women receiving single- and double-layer closure will be done as secondary analyses within each of the following subgroups separately:

For peer review only - http://bmjopen.bmj.com/site/about/guidelines.xhtml

1. Planned (without labour) or unplanned (in labour) CS
2. Emergency CS or not
3. Preterm (< 37 weeks gestational age) or term (≥ 37 week gestational age) CS
4. Presence (> 3cm) or absence (≤ 3cm) of dilatation
5. Placenta praevia or not
6. Presence or absence of specific maternal morbidity (e.g. diabetes, pre-eclampsia, haemolysis/elevated liver enzymes/low platelet count (HELLP) syndrome, immunodeficient women)
7. Singleton versus twin pregnancy
8. Natural cycle or hormonally induced withdrawal bleeding

Within the single-layer group (control group) we will compare the primary outcome between women in whom endometrial saving technique (split thickness) was applied and women in whom an endometrial saving technique was not applied (full thickness).

Economic evaluation

The economic evaluation will be performed alongside the RCT from a societal perspective. Both a cost-effectiveness and cost-utility analysis will be performed with a time horizon of nine months to relate the difference in societal and healthcare costs between double-layer and single-layer unlocked uterine closure during a CS to the difference in clinical effects. Healthcare costs include costs of primary and secondary care, complementary care and home care. Costs in other sectors include presence and absence from paid and unpaid work. The friction cost approach will be used to estimate indirect costs. For the valuation of health care utilization standard prices published in the Dutch Costing guidelines will be used. [34] Medication use will be valued using prices of the Royal Dutch Society for Pharmacy.

Societal costs will be related to the following effect measures in the economic evaluation: days with postmenstrual spotting and quality-adjusted life-years (QALYs) based on the Dutch tariff for the EuroQol (EQ-5D-5L). [27, 28, 35]

We hypothesise that double-layer uterine closure will reduce postmenstrual spotting and related consultations for gynaecological or fertility related problems and applied therapies, and as a consequence that it will be cost-effective in comparison with single-layer uterine closure.

The analysis will be done according to the intention to treat principle. Missing costs and effect data will be imputed using multiple imputation. Incremental cost-effectiveness ratios (ICERs) will be calculated by dividing the difference in mean total costs between the treatment groups by the difference in mean effects. Bootstrapping with 5000 replications will be used to estimate 95% CI around cost differences and the uncertainty surrounding the ICERs. Uncertainty surrounding the ICERs will be graphically presented on cost-effectiveness planes. Cost-effectiveness acceptability curves showing the probability that double-layer uterine closure is cost-effective in comparison with single-layer uterine closure for a range of different ceiling ratios will also be estimated. Adjustment for confounders and effect modifiers will be done if necessary. [36]

Interim analysis and safety monitoring

Because of the type of intervention, the Medical Ethics Committee (MEC) determined that the risk for participation is negligible. Therefore, we do not have a Data Safety Monitoring Committee. No interim analysis is planned.

All serious adverse events (SAEs) will be reported to the MEC by line listing yearly. Life threatening SAEs or an event that leads to death will be reported to the MEC immediately. All SAEs will be followed until they have abated, until a stable situation has been reached or the patient was discharged. We do not expect to terminate the study prematurely given the low risk of adverse events.

Confidentiality and data security

All participating centres receive a login name and password to gain access to ALEA 2.2, the web-secured randomisation database. Randomisation is performed pseudo-anonymously with only the initials and year of birth of the participants. Linking personal data to the study number can only be performed in the local participating centres or by the trial coordinator (SS). Written informed consent forms are stored in every centre in a lockable room. All forms and data will be archived for 15 years in the participating centres.

Discussion

In the last years, studies examining complications of CSs are increasing, including the development of niches or thin residual myometrium at the site of the previous CS and related symptoms. Both RMT and the presence of a niche have been associated with postmenstrual spotting. [8, 10] Double-layer unlocked closure has been shown to result in a thicker residual myometrium and as a consequence can possibly lead to a decrease of niche development after a CS compared to single-layer closure. [5, 22, 24, 37] However, the long-term clinical outcomes in terms of postmenstrual spotting or subfertility have not been studied previously or have not been related to ultrasound findings. We hypothesise that niche related postmenstrual spotting and fertility problems will reduce together with decrease in niche prevalence, in which

For peer review only

identification of the best uterine closure technique regarding RMT and niche development will be of great significance.

Strengths and limitations

The design of this study is one of the strengths; this is the first large RCT that will evaluate the effectiveness of double-layer uterine closure compared to single-layer uterine closure after CS regarding niche related gynaecological symptoms and reproductive outcomes with a long-term follow-up. The study is adequately powered. Randomisation is performed by using a web based randomisation program. Furthermore, all participants and examiners are blinded which reduces the chance for bias regarding reported symptoms and ultrasound findings. An additional strength is the uniform manner in which we try to perform double-layer closure and ultrasound evaluation, instructed by mandatory online instruction film and e-learning, respectively. Moreover, the 2Close study will compare the cost-effectiveness of both techniques which has never been done before. As we expect that double-layer closure will reduce the incidence of niche development and as a consequence that it could possibly reduce the gynaecological symptoms including postmenstrual spotting after CS, we assume double-layer closure to be more cost-effective. Also, we expect that double-layer closure will improve the chances of conceiving after CS and lower costs in fertility treatment.

We also expect some limitations. Baseline characteristics will be collected through questionnaires that are filled in by women in the first month after CS, which might lead to recall bias regarding medical history, complications during pregnancy and labour, and other baseline measurements. We decided to lower the administrative load for participating hospitals by obtaining these characteristics through the participants. Furthermore, there is no validated questionnaire available yet for postmenstrual spotting; therefore, the questionnaires that are used in the 2Close study are not adjusted or validated for these symptoms. Moreover, the surgical techniques performed during the CS in this study are standardised in both study arms except for saving the endometrium in the control group. There is no conclusive evidence whether or not to save the endometrium in the suture according to its influence on niche development. Therefore, we chose to leave this decision with the surgeons. There may possibly be a difference in the incidence of niche development between the participants receiving single-layer split thickness or full thickness closure, also when compared to the incidence of niche development in the double-layer group. This will be further examined in a subgroup analysis.

To prevent bias regarding niche evaluation three months after CS, all ultrasonographic examiners are trained by an online learning program and a sample of ultrasounds will be evaluated. The learning module is based on the results of a Delphi procedure among international niche experts. [11] Although the niche examiners in the 2Close are trained by a standardised method, experience in measuring niches and as a consequence differences in niche measurement may occur among examiners.

Potential impact and implications

This study will gain insight in the most optimal uterine closure technique after CS which is relevant for women and gynaecologists, since we will focus on long-term gynaecological symptoms and reproductive outcomes in relation to changes of the lower uterine segment after CS and in particular niche development. Since many studies have already shown that RMT and niches are related to several symptoms and therapies for niche resection are being developed, we think it is necessary to provide evidence for the development of preventive strategies regarding niche related symptoms. It is important to realise that the best way to prevent a niche and its related symptoms, is to not perform a CS. But since it is often inevitable to perform a CS, care takers should perform it in the most optimal way.

After the results of this study become available, the most optimal and cost-effective technique can be implemented in order to reduce symptoms and problems in a subsequent pregnancy. This will not be difficult, since the technique is easy to learn and many gynaecologists and residents are familiar with it after the trial. Especially for a scheduled CS, women should be informed about the risk to develop a niche and the risk that it might cause symptoms or complications later in life.

Additional files

Additional file 1: Affiliations of all 32 participating hospitals in the Netherlands that granted approval. The board of the hospitals granted approval to participate and to start recruiting patients. (DOCX 15 kb)

Additional file 2: Text of the online standardised instruction film for double-layer closure of the uterotomy. The spoken text in the online instruction film, which shows a standardised way to perform double-layer closure of the uterotomy, has been translated into English. (DOCX 15 kb)

Abbreviations

AMT: Adjacent myometrium thickness; BROK: Basic course Regulations and Organisation for Clinical researchers; CI: Confidence interval; eCRF: electronic case report form; CS: Caesarean section; GCP: Good clinical practice; GIS: Gel instillation sonohysterography; ICER: Incremental cost-effectiveness ratio; iMCQ: iMTA Medical Consumption Questionnaire; iMTA: Institute for Medical Technology Assessment; iPCQ: iMTA Productivity Cost Questionnaire; IQR: Interquartile range; MEC: Medical ethics committee; OR: Odds ratio; QALY: Quality adjusted life years; QOL: Quality of life; RCT: Randomised controlled trial; RMT: Residual myometrium thickness; RR: Relative risk; SAE: Serious adverse event; SD: Standard deviation; SIS: Saline infusion sonohysterography; TVUS: Transvaginal ultrasound; VAS: Visual analogue scale

For peer review only

Acknowledgements
None.

Funding
This study group received a grant from The Netherlands Organisation for Health Research and Development (ZonMw, project number: 843002605, grant number: 80–84300–98-62021), a Dutch organization that supports research in health and health innovation. The funder peer-reviewed the study design and will receive an annual progress report of the trial, but has no further role in the collection, analysis and interpretation of data, writing of the manuscript or decision to submit the report for publication.

Availability of data and materials
Data entry and secure storage is performed with data management system OpenClinica (OpenClinica LLC and collaborators, facilitated by TraIT (Translational Research IT infrastructure) and hosted by Vancis, which meets the ISO 27001 standard for information security management, version: 3.6). Data control during the trial is possible. The trial will be monitored on-site by a quality advisor of the Dutch Consortium for Studies in Women's Health and Reproduction. The quality advisor will also perform data verification at the end of the trial. The NVOG Consortium stores all data from clinical trials centrally so data will not become publicly available but will be available upon reasonable request to the corresponding author and with permission of the NVOG Consortium. Trial data will be accessible for verification or other research purposes, after an embargo period of 12 months in order to enable publication of the results by the project group.

Authors' contributions
JH, IJ, LvdV, MB, CdG, CL, RdL and WH made substantial contributions to the conception and design of the study. SS is responsible for the overall logistical aspects of the trial and general acquisition of data. SS and IJ drafted the manuscript. PV and JB provided statistical support regarding future analyses. EP, EB1, CR, MH, WvB, HV, JvL, HvV, RR, MS, CJ, WH, AF, KK, HS, JL, KdB, SC, DS, AO, MK, DP, LdV, EvB, MB, AH, WM, KD, EB2 and HvE made substantial contributions to acquisition of data. All authors critically revised the manuscript, approved the final version of the manuscript and agreed to be accountable for all aspects of the manuscript ensuring that questions related to the accuracy or integrity of the work are appropriately investigated and resolved.

Ethics approval and consent to participate
This study is approved by the ethics committee of Amsterdam UMC - location VUmc (reference number: 2015.462) and by the boards of all participating hospitals (Additional file 1). Protocol amendments will be communicated to participating hospitals, after approval of the central MEC of Amsterdam UMC – location VUmc. This trial is registered in the Dutch Trial Register (NTR5480, www.trialregister.nl). Counselling of eligible women will be done according to the 'good clinical practice' (GCP) guidelines or doctors certified according to Basic course Regulations and Organisation for Clinical researchers (BROK). After reading the patient information form and counselling by GCP- or BROK-certified personnel, women will be asked for written consent. This informed consent form will be obtained before participation in the clinical trial.
The research team declares that the SPIRIT guidelines were followed. All recommended items of the SPIRIT 2013 checklist were addressed in this clinical trial protocol.
The trial results will be communicated after the final inclusion and follow-up through publication in a peer-reviewed international journal and in an international congress. Participants will be informed through e-mail and the study website.

Consent for publication
Not applicable.

Competing interests
All authors declare that they have no competing interests.

Publisher's Note
Springer Nature remains neutral with regard to jurisdictional claims in published maps and institutional affiliations.

Author details
[1]Department of Obstetrics and Gynaecology, Research institutes 'Amsterdam Cardiovascular Sciences' and 'Amsterdam Reproduction and Development', Amsterdam UMC, Vrije Universiteit Amsterdam, De Boelelaan 1117, 1081 HV Amsterdam, Netherlands. [2]Department of Obstetrics and Gynaecology, Deventer Hospital, Nico Bolkesteinlaan 75, 7416 SE Deventer, the Netherlands. [3]Department of Obstetrics and Gynaecology, Máxima Medical Centre, De Run 4600, 5504 DB Veldhoven, the Netherlands. [4]Department of Epidemiology and Biostatistics, Vrije Universiteit Amsterdam, De Boelelaan 1105, 1081 HV Amsterdam, the Netherlands. [5]Department of Health sciences, Faculty of Science, Research institute 'Amsterdam Public Health', Vrije Universiteit Amsterdam, De Boelelaan 1085, 1081 HV Amsterdam, the Netherlands. [6]Department of Obstetrics and Gynaecology, Amsterdam UMC, University of Amsterdam, Meibergdreef 9, 1105 AZ Amsterdam, the Netherlands. [7]Department of Obstetrics and Gynaecology, OLVG-oost, Oosterpark 9, 1091 AC Amsterdam, the Netherlands. [8]Department of Obstetrics and Gynaecology, OLVG-west, Jan Tooropstraat 164, 1061 AE Amsterdam, the Netherlands. [9]Department of Obstetrics and Gynaecology, Westfriesgasthuis, Maelsonstraat 3, 1624 NP Hoorn, the Netherlands. [10]Department of Obstetrics and Gynaecology, Flevo hospital, Hospitaalweg 1, 1315 RA Almere, the Netherlands. [11]Department of Obstetrics and Gynaecology, Tergooi hospital, Rijksstraatweg 1, 1261 AN Blaricum, the Netherlands. [12]Department of Obstetrics and Gynaecology, Catharina hospital, Michelangelolaan 2, 5623 EJ Eindhoven, the Netherlands. [13]Department of Obstetrics and Gynaecology, Jeroen Bosch hospital, Henri Dunantstraat 1, 5223 GZ 's-Hertogenbosch, the Netherlands. [14]Department of Obstetrics and Gynaecology, Leiden University Medical Centre, Albinusdreef 2, 2333 ZA Leiden, the Netherlands. [15]Department of Obstetrics and Gynaecology, Groene Hart hospital, Bleulandweg 10, 2803 HH Gouda, the Netherlands. [16]Department of Obstetrics and Gynaecology, Haaglanden Medical Centre – Westeinde hospital, Lijnbaan 32, 2512 VA Den Haag, the Netherlands. [17]Department of Obstetrics and Gynaecology, Haga hospital, Els-Borst-Eilersplein 275, 2545 AA Den Haag, the Netherlands. [18]Department of Obstetrics and Gynaecology, Reinier de Graaf hospital, Reinier de Graafweg 5, 2625 AD Delft, the Netherlands. [19]Department of Obstetrics and Gynaecology, Research school 'GROW', Maastricht University Medical Centre, P. Debyelaan 25, 6229 HX Maastricht, the Netherlands. [20]Department of Obstetrics and Gynaecology, Zuyderland Medical Centre, Henri Dunantstraat 5, 6419 PC Heerlen, the Netherlands. [21]Department of Obstetrics and Gynaecology, Rijnstate hospital, Wagnerlaan 55, 6815 AD Arnhem, the Netherlands. [22]Department of Obstetrics and Gynaecology, Radboud University Nijmegen Medical Centre, Geert Grooteplein Zuid 10, 6525 GA Nijmegen, the Netherlands. [23]Department of Obstetrics and Gynaecology, Canisius-Wilhelmina hospital, Weg door Jonkerbos 100, 6532 SZ Nijmegen, the Netherlands. [24]Department of Obstetrics and Gynaecology, Bernhoven hospital, Nistelrodeseweg 10, 5406 PT Uden, the Netherlands. [25]Department of Obstetrics and Gynaecology, Röpcke-Zweers hospital, Jan Weitkamplaan 4a, 7772 SE Hardenberg, the Netherlands. [26]Department of Obstetrics and Gynaecology, Amphia hospital, Langendijk 75, 4819 EV Breda, the Netherlands. [27]Department of Obstetrics and Gynaecology, Sint Franciscus hospital, Kleiweg 500, 3045 PM Rotterdam, the Netherlands. [28]Department of Obstetrics and Gynaecology, Sint Antonius hospital, Koekoekslaan 1, 3435 CM Nieuwegein, the Netherlands. [29]Department of Obstetrics and Gynaecology, Birth Centre Wilhelmina Children hospital/ University Medical Centre Utrecht, Lundlaan 6, 3584 EA Utrecht, the Netherlands. [30]Department of Obstetrics and Gynaecology, Gelre hospital – location Apeldoorn, Albert Schweitzerlaan 31, 7334 DZ Apeldoorn, the Netherlands. [31]Department of Obstetrics and Gynaecology, Gelre hospital – location Zutphen, Den Elterweg 77, 7207 AE Zutphen, the Netherlands. [32]Department of Obstetrics and Gynaecology, Diakonessenhuis, Bosboomstraat 1, 3582 KE Utrecht, the Netherlands. [33]Department of Obstetrics and Gynaecology, Meander Medical Centre, Maatweg 3, 3813 TZ Amersfoort, the Netherlands. [34]Department of Obstetrics and Gynaecology, Isala clinics, Dokter van Heesweg 2, 8025 AB Zwolle, the Netherlands.

Received: 20 February 2018 Accepted: 12 February 2019
Published online: 04 March 2019

References
1. World Health Organization Human Reproduction Programme. WHO statement on caesarean section rates. Sex Reprod Health. 2015;02:1–8 (WHO/RHR/15.02).
2. Perined: Perinatale Zorg in Nederland 2016. In Utrecht: Perined; 2018.

For peer review only

3. Naji O, Wynants L, Smith A, Abdallah Y, Saso S, Stalder C, Van Huffel S, Ghaem-Maghami S, Van Calster B, Timmerman D, et al. Does the presence of a caesarean section scar affect implantation site and early pregnancy outcome in women attending an early pregnancy assessment unit? Hum Reprod. 2013;28(6):1489–96.
4. Naji O, Wynants L, Smith A, Abdallah Y, Stalder C, Sayasneh A, McIndoe A, Ghaem-Maghami S, Van Huffel S, Van Calster B, et al. Predicting successful vaginal birth after cesarean section using a model based on cesarean scar features examined by transvaginal sonography. Ultrasound Obstet Gynecol. 2013;41(6):672–8.
5. Roberge S, Demers S, Berghella V, Chaillet N, Moore L, Bujold E. Impact of single- vs double-layer closure on adverse outcomes and uterine scar defect: a systematic review and metaanalysis. Am J Obstet Gynecol. 2014; 211(5):453–60.
6. Hesselman S, Hogberg U, Ekholm-Selling K, Rassjo EB, Jonsson M. The risk of uterine rupture is not increased with single- compared with double-layer closure: a Swedish cohort study. BJOG. 2015;122(11):1535–41.
7. Clark EA, Silver RM. Long-term maternal morbidity associated with repeat cesarean delivery. Am J Obstet Gynecol. 2011;205(6 Suppl):S2–10.
8. Bij de Vaate AJ, Brolmann HA, van der Voet LF, van der Slikke JW, Veersema S, Huirne JA. Ultrasound evaluation of the cesarean scar: relation between a niche and postmenstrual spotting. Ultrasound Obstet Gynecol. 2011;37(1):93–9.
9. Bij de Vaate AJ, van der Voet LF, Naji O, Witmer M, Veersema S, Brolmann HA, Bourne T, Huirne JA. Prevalence, potential risk factors for development and symptoms related to the presence of uterine niches following cesarean section: systematic review. Ultrasound Obstet Gynecol. 2014;43(4):372–82.
10. van der Voet LF, Bij de Vaate AM, Veersema S, Brolmann HA, Huirne JA. Long-term complications of caesarean section. The niche in the scar: a prospective cohort study on niche prevalence and its relation to abnormal uterine bleeding. BJOG. 2014;121(2):236–44.
11. Jordans IPM, de Leeuw RA, Stegwee SI, Amso NN, Barri-Soldevila PN, van den Bosch T, Bourne T, Brolmann HAM, Donnez O, Dueholm M, et al. Sonographic examination of uterine niche in non-pregnant women: a modified Delphi procedure. Ultrasound Obstet Gynecol. 2019;53(1):107–15.
12. Gurol-Urganci I, Bou-Antoun S, Lim CP, Cromwell DA, Mahmood TA, Templeton A, van der Meulen JH. Impact of caesarean section on subsequent fertility: a systematic review and meta-analysis. Hum Reprod. 2013;28(7):1943–52.
13. Vervoort AJ, Uittenbogaard LB, Hehenkamp WJ, Brolmann HA, Mol BW, Huirne JA. Why do niches develop in caesarean uterine scars? Hypotheses on the aetiology of niche development. Hum Reprod. 2015;30(12):2695–702.
14. van der Voet LF, Vervoort AJ, Veersema S, Bij de Vaate AJ, Brolmann HA, Huirne JA. Minimally invasive therapy for gynaecological symptoms related to a niche in the caesarean scar: a systematic review. BJOG. 2014;121(2): 145–56.
15. Vervoort A, Vissers J, Hehenkamp WJK, Brolmann HAM, Huirne JAF. The effect of laparoscopic resection of large niches in the uterine caesarean scar on symptoms, ultrasound findings and quality of life: a prospective cohort study. BJOG. 2018;125(3):317–25.
16. Vervoort AJ, Van der Voet LF, Witmer M, Thurkow AL, Radder CM, van Kesteren PJ, Quartero HW, Kuchenbecker WK, Bongers MY, Geomini PM, et al. The HysNiche trial: hysteroscopic resection of uterine caesarean scar defect (niche) in patients with abnormal bleeding, a randomised controlled trial. BMC Womens Health. 2015;15:103.
17. Nikkels C, Vervoort A, Mol BW, Hehenkamp WJK, Huirne JAF, Brolmann HAM. IDEAL framework in surgical innovation applied on laparoscopic niche repair. Eur J Obstet Gynecol Reprod Biol. 2017;215:247–53.
18. Huirne JAF, Vervoort A, Leeuw R, Brolmann HAM, Hehenkamp WJK. Technical aspects of the laparoscopic niche resection, a step-by-step tutorial. Eur J Obstet Gynecol Reprod Biol. 2017;219:106–12.
19. Vervoort A, van der Voet LF, Hehenkamp W, Thurkow AL, van Kesteren P, Quartero H, Kuchenbecker W, Bongers M, Geomini P, de Vleeschouwer L, et al. Hysteroscopic resection of a uterine caesarean scar defect (niche) in women with postmenstrual spotting: a randomised controlled trial. BJOG. 2018;125(3): 326–34.
20. Abalos E, Addo V, Brocklehurst P, El Sheikh M, Farrell B, Gray S, Hardy P, Juszczak E, Mathews JE, Masood SN, et al. Caesarean section surgical techniques (CORONIS): a fractional, factorial, unmasked, randomised controlled trial. Lancet. 2013;382(9888):234–48.
21. The CAESAR study collaborative group. Caesarean section surgical techniques: a randomised factorial trial (CAESAR). BJOG. 2010;117(11):1366–76.
22. Di Spiezio Sardo A, Saccone G, McCurdy R, Bujold E, Bifulco G, Berghella V. Risk of cesarean scar defect in single- versus double-layer uterine closure: a systematic review and meta-analysis of randomized controlled trials. Ultrasound Obstet Gynecol. 2017;50:578–83.
23. Dodd JM, Anderson ER, Gates S, Grivell RM. Surgical techniques for uterine incision and uterine closure at the time of caesarean section. Cochrane Database Syst Rev. 2014;7:CD004732.
24. Stegwee SI, Jordans I, van der Voet LF, van de Ven PM, Ket J, Lambalk CB, de Groot C, Hehenkamp W, Huirne J. Uterine caesarean closure techniques affect ultrasound findings and maternal outcomes: a systematic review and meta-analysis. BJOG. 2018;125(9):1097–108.
25. Osser OV, Jokubkiene L, Valentin L. Cesarean section scar defects: agreement between transvaginal sonographic findings with and without saline contrast enhancement. Ultrasound Obstet Gynecol. 2010;35(1):75–83.
26. Aaronson NK, Muller M, Cohen PD, Essink-Bot ML, Fekkes M, Sanderman R, Sprangers MA, te Velde A, Verrips E. Translation, validation, and norming of the Dutch language version of the SF-36 health survey in community and chronic disease populations. J Clin Epidemiol. 1998;51(11):1055–68.
27. Dolan P. Modeling valuations for EuroQol health states. Med Care. 1997; 35(11):1095–108.
28. EuroQol--a new facility for the measurement of health-related quality of life. Health Policy. 1990;16(3):199–208.
29. Ability to Participate in Social Roles and Activities, Short Form 8a [http://www.healthmeasures.net/explore-measurement-systems/promis/intro-to-promis/list-of-adult-measures].
30. Rosen R, Brown C, Heiman J, Leiblum S, Meston C, Shabsigh R, Ferguson D, D'Agostino R Jr. The female sexual function index (FSFI): a multidimensional self-report instrument for the assessment of female sexual function. J Sex Marital Ther. 2000;26(2):191–208.
31. Handleiding iMTA Productivity Cost Questionnaire (iPCQ). [http://www.imta.nl].
32. Handleiding iMTA Medical Cost Questionnaire (iMCQ). [http://www.imta.nl].
33. Maarse M, Bij de Vaate AJM, Huirne JAF, Brölmann HAM. De maandkalender, een hulpmiddel voor een efficiënte menstruatieanamnese. Ned Tijdschr Obstet Gynaecol. 2011;124:231–5.
34. Hakkaart-van Roijen L, van der Linden N, Bouwmans CAM, Kanters TA, Tan SS. Kostenhandleiding: Methodologie van kostenonderzoek en referentieprijzen voor economische evaluaties in de gezondheidszorg. Institute for Medical Technology Assessment; Erasmus Universiteit Rotterdam. Zorginstituut Nederland; 2016.
35. Lamers LM, Stalmeier PF, McDonnell J, Krabbe PF, van Busschbach JJ. Measuring the quality of life in economic evaluations: the Dutch EQ-5D tariff. Ned Tijdschr Geneeskd. 2005;149(28):1574–8.
36. Fenwick E, O'Brien BJ, Briggs A. Cost-effectiveness acceptability curves--facts, fallacies and frequently asked questions. Health Econ. 2004;13(5):405–15.
37. Glavind J, Madsen LD, Uldbjerg N, Dueholm M. Ultrasound evaluation of cesarean scar after single- and double-layer uterotomy closure: a cohort study. Ultrasound Obstet Gynecol. 2013;42(2):207–12.

Ready to submit your research? Choose BMC and benefit from:

- fast, convenient online submission
- thorough peer review by experienced researchers in your field
- rapid publication on acceptance
- support for research data, including large and complex data types
- gold Open Access which fosters wider collaboration and increased citations
- maximum visibility for your research: over 100M website views per year

At BMC, research is always in progress.

Learn more biomedcentral.com/submissions

BMC

For peer review only - http://bmjopen.bmj.com/site/about/guidelines.xhtml

CHEERS Checklist
Items to include when reporting economic evaluations of health interventions

The **ISPOR CHEERS Task Force Report**, *Consolidated Health Economic Evaluation Reporting Standards (CHEERS)—Explanation and Elaboration: A Report of the ISPOR Health Economic Evaluations Publication Guidelines Good Reporting Practices Task Force,* provides examples and further discussion of the 24-item CHEERS Checklist and the CHEERS Statement. It may be accessed via the *Value in Health* or via the ISPOR Health Economic Evaluation Publication Guidelines – CHEERS: Good Reporting Practices webpage: http://www.ispor.org/TaskForces/EconomicPubGuidelines.asp

Section/item	Item No	Recommendation	Reported on page No/ line No
**Title and abstract**			
Title	1	Identify the study as an economic evaluation or use more specific terms such as "cost-effectiveness analysis", and describe the interventions compared.	1
Abstract	2	Provide a structured summary of objectives, perspective, setting, methods (including study design and inputs), results (including base case and uncertainty analyses), and conclusions.	2
**Introduction**			
Background and objectives	3	Provide an explicit statement of the broader context for the study. Present the study question and its relevance for health policy or practice decisions.	4
**Methods**			
Target population and subgroups	4	Describe characteristics of the base case population and subgroups analysed, including why they were chosen.	6
Setting and location	5	State relevant aspects of the system(s) in which the decision(s) need(s) to be made.	7, 8
Study perspective	6	Describe the perspective of the study and relate this to the costs being evaluated.	7
Comparators	7	Describe the interventions or strategies being compared and state why they were chosen.	8
Time horizon	8	State the time horizon(s) over which costs and consequences are being evaluated and say why appropriate.	7
Discount rate	9	Report the choice of discount rate(s) used for costs and outcomes and say why appropriate.	7
Choice of health outcomes	10	Describe what outcomes were used as the measure(s) of benefit in the evaluation and their relevance for the type of analysis performed.	8-10
Measurement of effectiveness	11a	*Single study-based estimates:* Describe fully the design features of the single effectiveness study and why the single study was a sufficient source of clinical effectiveness data.	8, 9

	11b	*Synthesis-based estimates:* Describe fully the methods used for identification of included studies and synthesis of clinical effectiveness data.	NA
Measurement and valuation of preference based outcomes	12	If applicable, describe the population and methods used to elicit preferences for outcomes.	8 - 10
Estimating resources and costs	13a	*Single study-based economic evaluation:* Describe approaches used to estimate resource use associated with the alternative interventions. Describe primary or secondary research methods for valuing each resource item in terms of its unit cost. Describe any adjustments made to approximate to opportunity costs.	9, 10
	13b	*Model-based economic evaluation:* Describe approaches and data sources used to estimate resource use associated with model health states. Describe primary or secondary research methods for valuing each resource item in terms of its unit cost. Describe any adjustments made to approximate to opportunity costs.	NA
Currency, price date, and conversion	14	Report the dates of the estimated resource quantities and unit costs. Describe methods for adjusting estimated unit costs to the year of reported costs if necessary. Describe methods for converting costs into a common currency base and the exchange rate.	9, 10 table S1
Choice of model	15	Describe and give reasons for the specific type of decision-analytical model used. Providing a figure to show model structure is strongly recommended.	NA
Assumptions	16	Describe all structural or other assumptions underpinning the decision-analytical model.	NA
Analytical methods	17	Describe all analytical methods supporting the evaluation. This could include methods for dealing with skewed, missing, or censored data; extrapolation methods; methods for pooling data; approaches to validate or make adjustments (such as half cycle corrections) to a model; and methods for handling population heterogeneity and uncertainty.	10 - 12
**Results**			
Study parameters	18	Report the values, ranges, references, and, if used, probability distributions for all parameters. Report reasons or sources for distributions used to represent uncertainty where appropriate. Providing a table to show the input values is strongly recommended.	NA
Incremental costs and outcomes	19	For each intervention, report mean values for the main categories of estimated costs and outcomes of interest, as well as mean differences between the comparator groups. If applicable, report incremental cost-effectiveness ratios.	13 - 15 Table 2+3
Characterising uncertainty	20a	*Single study-based economic evaluation:* Describe the effects of sampling uncertainty for the estimated incremental cost and incremental effectiveness parameters, together with the impact	11

		of methodological assumptions (such as discount rate, study perspective).	
	20b	*Model-based economic evaluation:* Describe the effects on the results of uncertainty for all input parameters, and uncertainty related to the structure of the model and assumptions.	NA
Characterising heterogeneity	21	If applicable, report differences in costs, outcomes, or cost-effectiveness that can be explained by variations between subgroups of patients with different baseline characteristics or other observed variability in effects that are not reducible by more information.	12 ; 15
**Discussion**			
Study findings, limitations, generalisability, and current knowledge	22	Summarise key study findings and describe how they support the conclusions reached. Discuss limitations and the generalisability of the findings and how the findings fit with current knowledge.	16-18
**Other**			
Source of funding	23	Describe how the study was funded and the role of the funder in the identification, design, conduct, and reporting of the analysis. Describe other non-monetary sources of support.	19
Conflicts of interest	24	Describe any potential for conflict of interest of study contributors in accordance with journal policy. In the absence of a journal policy, we recommend authors comply with International Committee of Medical Journal Editors recommendations.	19, 20

For consistency, the CHEERS Statement checklist format is based on the format of the CONSORT statement checklist

The **ISPOR CHEERS Task Force Report** provides examples and further discussion of the 24-item CHEERS Checklist and the CHEERS Statement. It may be accessed via the *Value in Health* link or via the ISPOR Health Economic Evaluation Publication Guidelines – CHEERS: Good Reporting Practices webpage: http://www.ispor.org/TaskForces/EconomicPubGuidelines.asp

The citation for the CHEERS Task Force Report is:
Husereau D, Drummond M, Petrou S, et al. Consolidated health economic evaluation reporting standards (CHEERS)—Explanation and elaboration: A report of the ISPOR health economic evaluations publication guidelines good reporting practices task force. Value Health 2013;16:231-50.
